# Causal association between adiposity and hemorrhoids: a Mendelian randomization study

**DOI:** 10.3389/fmed.2023.1229925

**Published:** 2023-10-06

**Authors:** Jian Huang, Ying Gui, Hongping Qin, Yubo Xie

**Affiliations:** ^1^Clinical Laboratory Center, The First Affiliated Hospital of Guangxi Medical University, Nanning, China; ^2^Department of Anesthesiology, The First Affiliated Hospital of Guangxi Medical University, Nanning, China; ^3^Guangxi Key Laboratory of Enhanced Recovery After Surgery for Gastrointestinal Cancer, The First Affiliated Hospital of Guangxi Medical University, Nanning, China

**Keywords:** adiposity, hemorrhoids, Mendelian randomization, risk, body mass index

## Abstract

**Background:**

Hemorrhoids are a very common anorectal disorder affecting a large number of individuals throughout the world. This study aimed to evaluate the causal effects of four adiposity traits including body mass index (BMI), body fat percentage, waist circumference, and waist-to-hip ratio on hemorrhoids by Mendelian randomization (MR).

**Methods:**

We used summary statistics of BMI (*N* = 461,460), body fat percentage (*N* = 454,633), waist circumference (*N* = 462,166), waist-to-hip ratio (*N* = 212,244), and hemorrhoids (*N* = 337,199) from large-scale genome wide association studies of European ancestry. Univariable and multivariable MR were carried out to infer causality. The MR Steiger directionality test was used to test the causal direction.

**Results:**

The primary MR analysis using the inverse variance weighted (IVW) method showed that there were positive effects of genetically determined BMI [odds ratio (OR) = 1.005, 95% confidence interval (CI): 1.003–1.008, per standard deviation (SD), *p* = 7.801 × 10^−5^], body fat percentage (OR = 1.005, 95% CI: 1.001–1.008, per SD, *p* = 0.008), waist circumference (OR = 1.008, 95% CI: 1.005–1.011, per SD, *p* = 1.051 × 10^−6^), and waist-to-hip ratio (OR = 1.010, 95% CI: 1.003–1.017, per SD, *p* = 0.003) on hemorrhoids. These findings were robust in multivariable MR adjusting for physical activity. The Steiger directionality test showed evidence against reverse causation.

**Conclusion:**

Our MR study supports a causal role of adiposity in the development of hemorrhoids. Adiposity prevention may be an important strategy for reducing hemorrhoids risk.

## Introduction

Hemorrhoids are one of the most common anorectal disorders encountered in colorectal practice. According to epidemiological data, as many as half of the population suffers from hemorrhoids before reaching the age of 50 (1). Both males and females are affected equally (2). In hemorrhoids, the most common symptom is painless rectal bleeding during defection (3). Depending on their location, hemorrhoids are generally divided into external, internal, and mixed type (3). Hemorrhoids treatment may include a variety of non-operative and surgical options. Approximately 10% of cases require surgical procedures (4). In the United Kingdom (UK), more than 20,000 hemorrhoidal procedures are performed annually (5). Given that hemorrhoids are associated with a heavy healthcare and economic burden, increasing attention has been given to hemorrhoids prevention in recent years (5).

The cause of hemorrhoids remains unclear. Epidemiological studies have suggested several risk factors for the development of hemorrhoids including adiposity (6). However, evidence concerning the relationship between adiposity and hemorrhoids is scarce and inconclusive (7–11), and it is not yet established whether the association is causal. The practical inability to perform randomized controlled trials (RCTs) for evaluating the causality between adiposity and hemorrhoids makes it necessary to conduct a Mendelian randomization (MR) analysis. MR is a form of causal inference method that utilizes single nucleotide polymorphisms (SNPs) as instrumental variables to explore the potential causal relationship between an exposure and a disorder (12). In MR, the biases including residual confounding and reverse causation are significantly reduced (13). Because identifying modifiable risk factors can offer potential for hemorrhoids prevention, it is of importance to infer causation between adiposity and hemorrhoids. The aim of this study is to apply the MR causal framework to evaluate whether four adiposity traits including body mass index (BMI), body fat percentage, waist circumference, and waist-to-hip ratio have causal effects on hemorrhoids development.

## Methods

### Study design

This was a two-sample MR study utilizing publicly available data for all analyses. Our paper was constructed in accordance with the suggestions provided in the Strengthening the Reporting of Observational Studies in Epidemiology-Mendelian randomization (STROBE-MR) guidelines. Supplementary Table S1 shows our STROBE-MR checklist.

### Data sources for exposures

The summary-level data for BMI (*N* = 461,460), body fat percentage (*N* = 454,633) and waist circumference (*N* = 462,166) were obtained from the Medical Research Council-Integrative Epidemiology Unit (MRC-IEU) consortium. According to the consortium’s instructions, body composition measures were taken manually.^1^ For BMI, units of measurement were Kg/m^2^. Regarding body fat percentage, units of measurement were percent. For waist circumference, units of measurement were cm. Waist-to-hip ratio data were derived from a genome wide association study (GWAS) meta-analysis of the Genetic Investigation of Anthropometric Traits (GIANT) consortium, which was based on 212,244 participants of European descent (14). Table 1 shows the details on the GWAS summary statistics for the exposures.

**Table 1:** The GWAS datasets included in this MR study.

Trait	Sample size	Population	Unit	Sex	GWAS-ID	Consortium	Year
Body mass index	461,460	European	SD	Males and females	ukb-b-19953	MRC-IEU	2018
Body fat percentage	454,633	European	SD	Males and females	ukb-b-8909	MRC-IEU	2018
Waist circumference	462,166	European	SD	Males and females	ukb-b-9405	MRC-IEU	2018
Waist-to-hip ratio	212,244	European	SD	Males and females	ieu-a-73	GIANT	2015
Hemorrhoids	8,190 cases and 329,009 controls	European	NA	Males and females	ukb-a-539	Neale Lab	2017
Physical activity	24,264	European	NA	Males and females	ieu-b-4859	Within family GWAS consortium	2022

GWAS, genome-wide association studies; GIANT, Genetic Investigation of Anthropometric Traits; MRC-IEU, Medical Research Council-Integrative Epidemiology Unit; SD, standard deviation.

### Outcome data

For hemorrhoids, we used summary-level data computed by the Neale Lab, which included 8,190 cases and 329,009 controls. Diagnostic code applied for identifying hemorrhoids cases was I84 in international Classification of diseases (ICD)-10.

### Instrument construction

For constructing genetic instruments for each exposure, we selected SNPs strongly associated with the exposure at genome-wide significance (*p* < 5 × 10^−8^). Selected SNPs were then taken forward to linkage disequilibrium clumping to remove SNPs that were correlated (*r*^2^ ≥ 0.001). A European reference panel of the 1,000 Genomes Project was used as reference population (15). We harmonized SNP-exposure and SNP-hemorrhoids associations to align the effect sizes and to exclude palindromic SNPs. In the hemorrhoids GWAS dataset, we used proxy SNPs (r^2^ > 0.8) when particular SNPs were absent. The SNP extraction from GWAS summary-level data, clumping, and harmonization were performed using the TwoSample MR package in R version 4.1.0 (16, 17). The F-statistic was used to quantify instrument strength. It was calculated using the equation: F = (R2/K)/[(1−R2)(N−K−1)] (18). Where R2 is the variance explained by the instruments, K is the number of instruments, and N is the sample size.

### Statistical analyses

Using two-sample MR, we generated estimates of the causal effect of the adiposity measures on hemorrhoids (OR per SD unit increase). We used the inverse variance weighted (IVW) method as the primary MR analysis (19); it assumes that the genetic instruments as a whole meets the key MR assumptions. To further evaluate the causal assessments identified in the primary MR analysis, additional sensitivity analyses using MR-Egger, maximum likelihood, weighted median, and MR Pleiotropy RESidual Sum and Outlier (MR-PRESSO) methods were undertaken. Certain MR assumptions were relaxed using these methods; they are more robust to potential pleiotropic effects (20, 21). For investigating horizontal pleiotropy, the intercept term from MR-Egger regression was used (22). The intercept *p* > 0.05 indicates the absence of pleiotropy. Furthermore, we used the MR-PRESSO as an additional method for detecting pleiotropy; this method can exclude outlier genetic instruments having pleiotropic effects and carry out causal effect estimates after excluding outlying genetic instruments (23). We investigated the causal direction between the adiposity measures and hemorrhoids using the MR Steiger directionality test (16, 24). For accounting for physical activity’s effects on our MR assessments, multivariable MR analyses were undertaken using the TwoSampleMR package. Summary-level data for physical activity were obtained from the Within family GWAS consortium involving 24,264 participants of European descent (Table 1).

We did not pre-register the study protocol. All tests were two-sided and conducted using the TwoSampleMR and MR-PRESSO packages in R version 4.1.0. Statistical significance was set at *p <* 0.05. Ethical approval and informed consent were obtained in all original GWASs. Since we only analyzed publicly available data, we did not seek ethical approval from the local committee.

## Results

**Figure 1:**
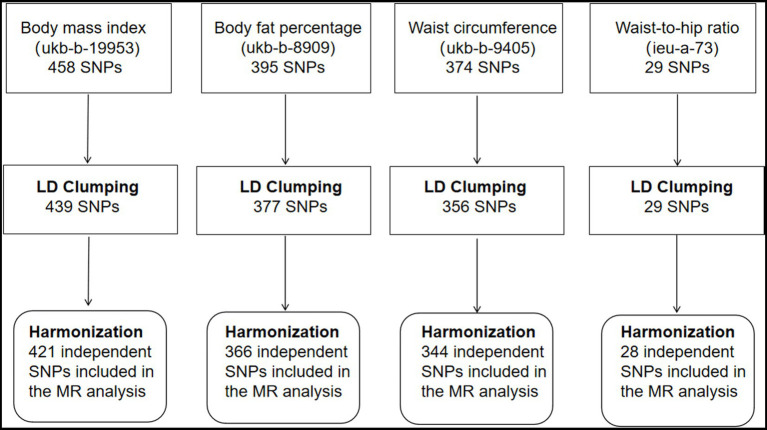
Selection of instrumental single nucleotide polymorphisms for adiposity traits. SNP, single nucleotide polymorphism; LD, linkage disequilibrium; MR, Mendelian randomization.

A summary of the instrument SNP selection process is shown in Figure 1. Of the 458 SNPs that predicted BMI, 19 SNPs were removed after linkage disequilibrium clumping. Harmonization with the corresponding hemorrhoids summary-level data resulted in 421 independent SNPs for the MR analysis (F-statistic = 61.13). Of the 395 SNPs predicting body fat percentage, linkage disequilibrium clumping removed 18 SNPs. Three hundred and sixty-six SNPs were obtained for the MR analysis after harmonizing the body fat percentage dataset with the hemorrhoids dataset (F-statistic = 32.86). Of the 374 SNPs that predicted waist circumference, 18 correlated SNPs were removed after linkage disequilibrium clumping. Harmonization with the corresponding hemorrhoids summary statistics produced 344 SNPs for the MR analysis (F-statistic = 45.00). The 29 SNPs predicting waist-to-hip ratio were uncorrelated (*r*^2^ < 0.001). Harmonization with the corresponding hemorrhoids summary statistics obtained 28 SNPs for the MR analysis (F-statistic = 76.56). The SNPs for each exposure had enough strength; the F-statistics were greater than 10. Supplementary Tables S2–S5 show the details on the instrumental genetic variants for each exposure.

**Figure 2:**
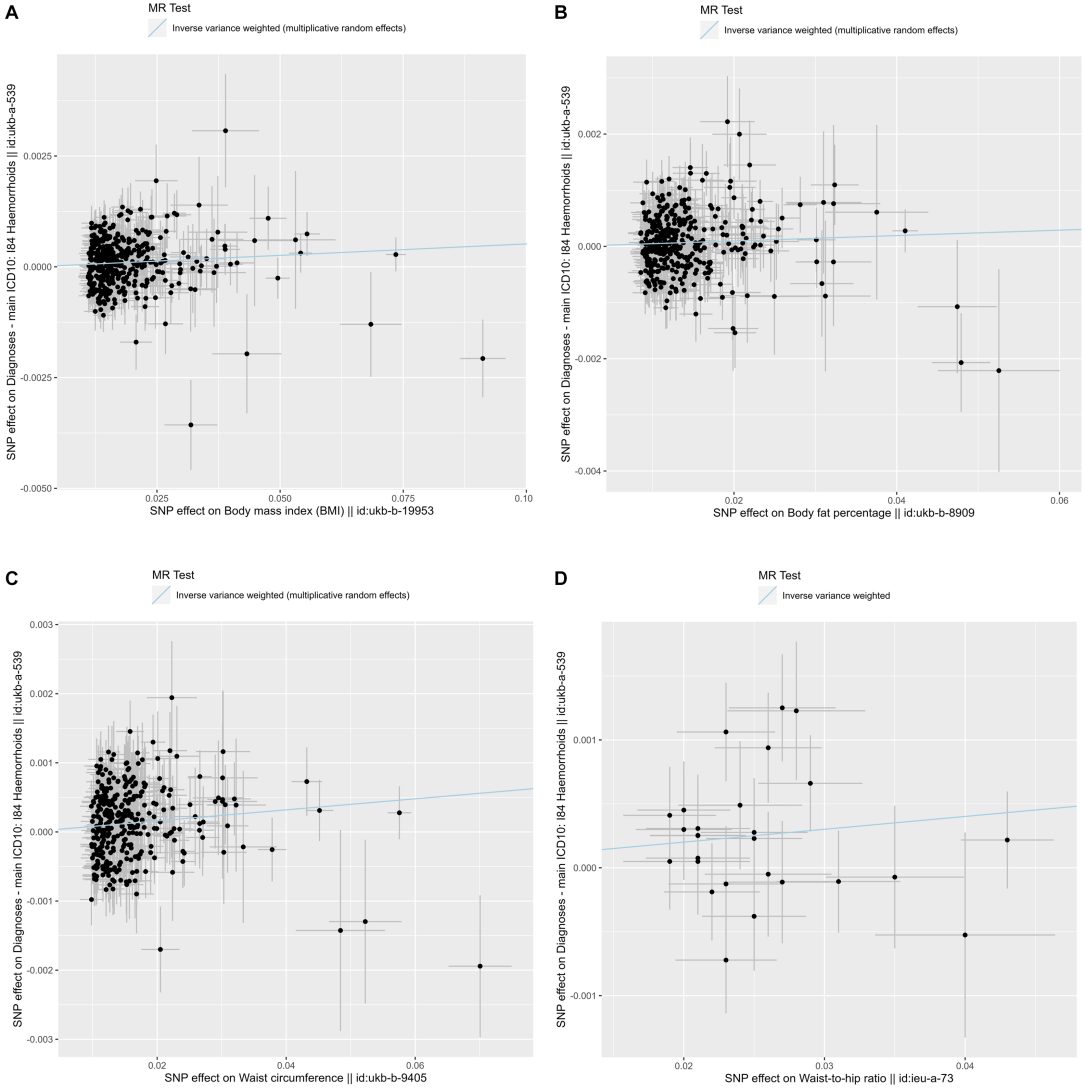
Scatter plots of the primary Mendelian randomization analysis assessing the causal effect of four adiposity traits on hemorrhoids. **(A)** Scatter plot for the causal effect of body mass index on hemorrhoids. **(B)** Scatter plot for the causal effect of body fat percentage on hemorrhoids. **(C)** Scatter plot for the causal effect of waist circumference on hemorrhoids. **(D)** Scatter plot for the causal effect of waist-to-hip ratio on hemorrhoids. The genetic association with exposure is represented by the x-axis, while the genetic association with hemorrhoids risk is represented by the y-axis. Results were obtained using inverse variance weighted Mendelian randomization. SNP, single nucleotide polymorphism; MR, Mendelian randomization.

**Figure 3:**
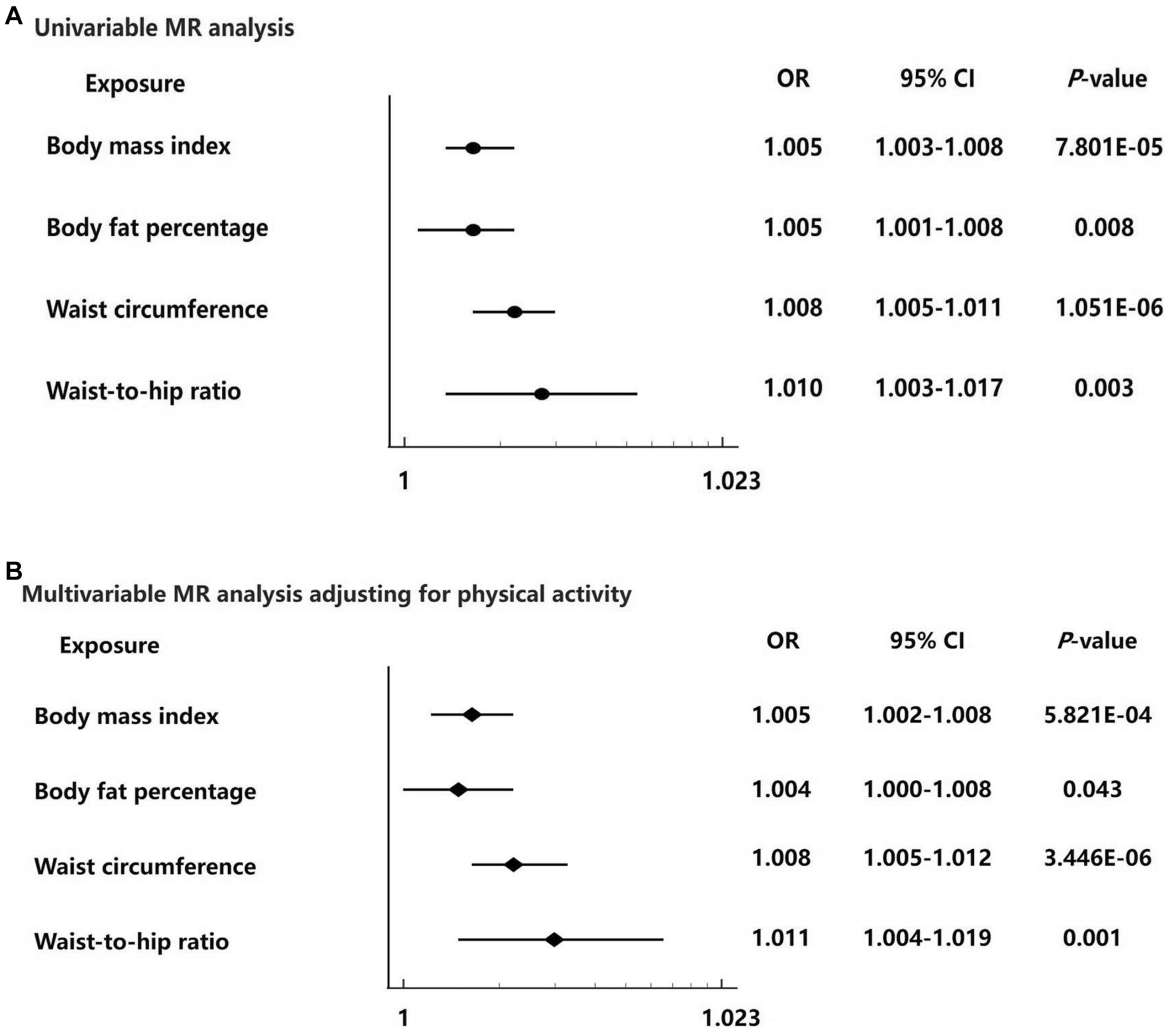
Forest plots for the univariable and multivariable Mendelian randomization analysis on the causal effect of adiposity on hemorrhoids risk. **(A)** Univariable Mendelian randomization analysis from the inverse variance weighted method of four adiposity traits including body mass index, body fat percentage, waist circumference, and waist-to-hip ratio with hemorrhoids risk. **(B)** Multivariable Mendelian randomization analysis of four adiposity traits including body mass index, body fat percentage, waist circumference, and waist-to-hip ratio with hemorrhoids risk adjusting for physical activity. Data are displayed as odds ratio (OR) and 95% confidence interval (CI).

For each 1-SD kg/m^2^ increase in genetically determined BMI, the IVW MR analysis indicated enhanced risk of hemorrhoids [odds ratio (OR) = 1.005, 95% confidence interval (CI): 1.003–1.008, *p* = 7.801 × 10^−5^] (Figures 2A, 3A). This finding was consistent across sensitivity analyses applying the maximum likelihood (OR = 1.004, 95% CI: 1.002–1.006, *p* = 0.001) and MR-PRESSO (OR = 1.004, 95% CI: 1.002–1.006, *p* = 9.470 × 10^−5^) models. We observed heterogeneity across the SNPs (Cochran’s Q *p* = 9.470 × 10^−5^). The MR-Egger intercept was centered around zero (intercept = 2.845 × 10^−5^, *p* = 0.658), which was suggestive of the absence of a directional pleiotropic effect. MR-PRESSO detected one outlying SNP, but the MR estimate was unchanged when removing it (OR = 1.005, 95% CI: 1.003–1.008, *p* = 4.707 × 10^−5^). Results of the leave-one-out sensitivity analysis supported the observed causal association (Supplementary Table S6).

The IVW MR analysis showed that the OR per 1-SD increase in genetically determined body fat percentage for the risk of hemorrhoids was 1.005 (95% CI: 1.001–1.008, *p* = 0.008) (Figures 2B, 3A). The causal estimates were also significant by sensitivity analyses using the maximum likelihood (OR = 1.005, 95% CI: 1.002–1.008, *p* = 0.004) and MR-PRESSO (OR = 1.005, 95% CI: 1.001–1.008, *p* = 0.008) methods. Heterogeneity was noted among studies (*p* = 0.005). However, this was likely not due to horizontal pleiotropy, because the MR-Egger regression intercept analysis yielded a large *p*-value (intercept = 7.297 × 10^−7^, *p* = 0.992). No outliers were identified using the MR-PRESSO method. The leave-one-out sensitivity analysis did not detect any individual SNPs that significantly affected the causal estimates (Supplementary Table S7).

In the primary MR analysis using the IVW method, the OR per 1-SD increase in genetically determined waist circumference was 1.008 (95% CI: 1.005–1.011, *p* = 1.051 × 10^−6^) for hemorrhoids (Figures 2C, 3A). The sensitivity analyses based on the maximum likelihood (OR = 1.008, 95% CI: 1.005–1.011, *p* = 6.891 × 10^−8^), weight median (OR = 1.006, 95% CI: 1.001–1.011, *p* = 0.028), and MR-PRESSO (OR = 1.008, 95% CI: 1.005–1.011, *p* = 1.614 × 10^−6^) methods yielded similar results. Although the Cochran’s Q test showed evidence of heterogeneity (*p* = 0.002), it was not likely because of horizontal pleiotropy (MR-Egger intercept = 2.687 × 10^−6^, *p* = 0.970). MR-PRESSO did not identify potential outliers. The leave-one-out sensitivity analysis found no individual SNPs significantly affecting the causal estimates (Supplementary Table S8).

For each 1-SD increase in genetically determined waist-to-hip ratio, increased risk of hemorrhoids was identified in the IVW estimate (OR = 1.010, 95% CI: 1.003–1.017, *p* = 0.003) (Figures 2D, 3A). The maximum likelihood (OR = 1.010, 95% CI: 1.004–1.017, *p* = 0.001) and MR-PRESSO (OR = 1.010, 95% CI: 1.003–1.017, *p* = 0.007) analyses supported this association with consistent effect sizes. There was no evidence for heterogeneity (*p* = 0.203). The MR-Egger intercept indicated absence of horizontal pleiotropy (intercept = 2.670 × 10^−4^, *p* = 0.512). No significant outliers were detected using the MR-PRESSO method. The leave-one-out sensitivity analysis showed that no single SNPs could dramatically drive the observed causal association (Supplementary Table S9).

**Figure 4:**
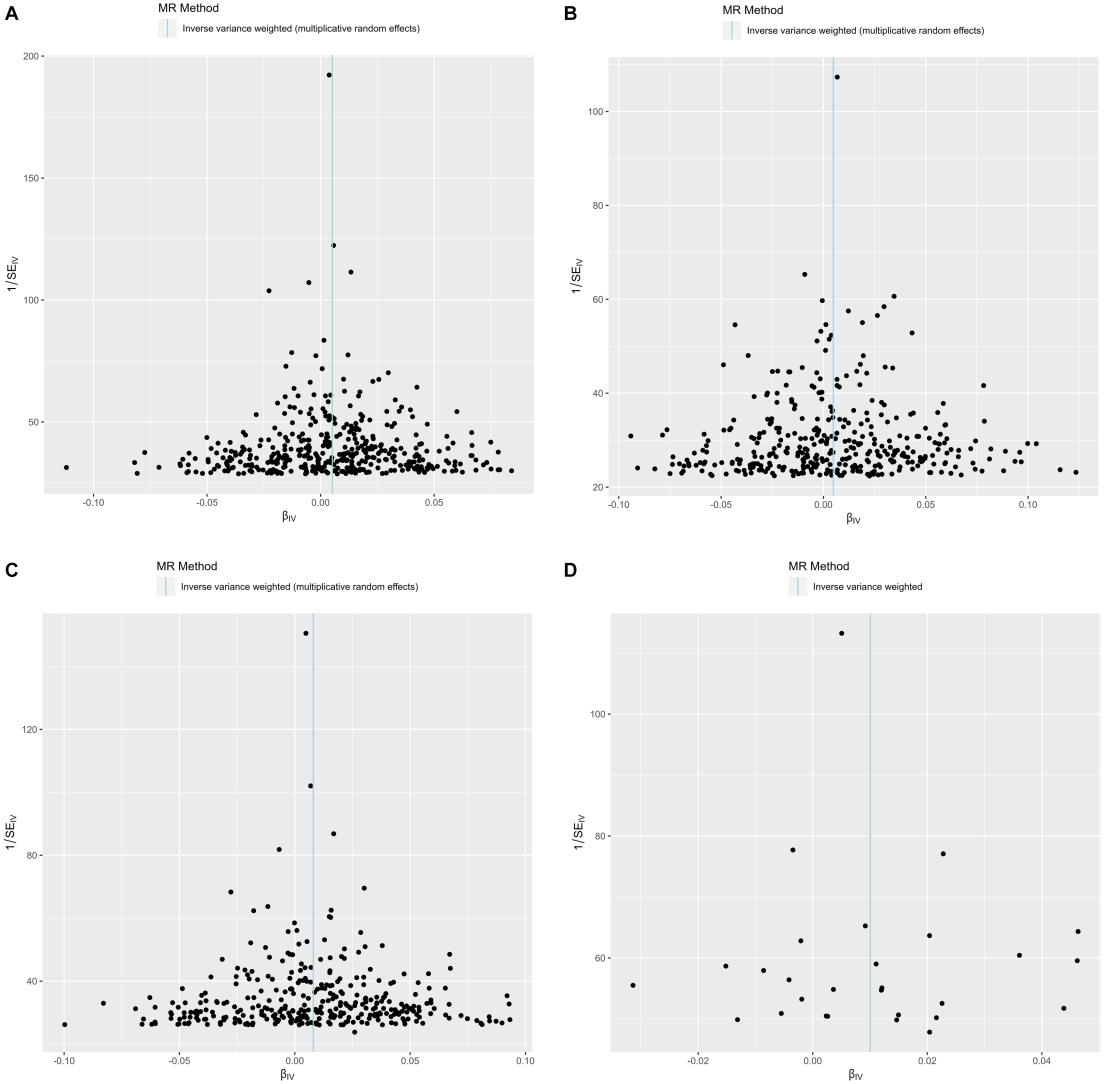
Funnel plots of precision (1/SE) versus causal estimate β for hemorrhoids. **(A)** Exposure: body mass index. **(B)** Exposure: body fat percentage. **(C)** Exposure: waist circumference. **(D)** Exposure: waist-to-hip ratio.

The funnel plots showed symmetry, supporting the reliability of the MR analyses (Figure 4). The MR Steiger directionality test was used to test the causal direction between the adiposity measures and hemorrhoids. Table 2 shows the results. The variance in the outcome (snp_r2.outcome) was less than each exposure (snp_r2.exposure), confirming the causal direction.

**Table 2:** MR Steiger directionality test for evaluating the causal direction.

Exposure	Outcome	snp_r2.exposure	snp_r2.outcome	Causal direction
Body mass index	Hemorrhoids	0.06	1.71 × 10^−3^	True
Body fat percentage	Hemorrhoids	0.05	1.35 × 10^−3^	True
Waist circumference	Hemorrhoids	0.04	1.39 × 10^−3^	True
Waist-to-hip ratio	Hemorrhoids	6.74 × 10^−3^	1.38 × 10^−4^	True

Finally, we performed multivariable MR analysis to adjust for physical activity. The results showed statistically significant causal associations of genetically determined BMI (OR = 1.005, 95% CI: 1.002–1.008, *p* = 5.821 × 10^−4^), body fat percentage (OR = 1.004, 95% CI: 1.000–1.008, *p* = 0.043), waist circumference (OR = 1.008, 95% CI: 1.005–1.012, *p* = 3.446 × 10^−6^), and waist-to-hip ratio (OR = 1.011, 95% CI: 1.004–1.019, *p* = 0.001) with hemorrhoids (Figure 3B).

## Discussion

In order to evaluate the effects of adiposity traits including BMI, body fat percentage, waist circumference, and waist-to-hip ratio on hemorrhoids risk, we used large-scale GWAS summary-level data within the MR framework. Our MR analyses showed that genetically determined BMI, body fat percentage, waist circumference, and waist-to-hip ratio were causally associated with increased risk of hemorrhoids. These findings indicated an important role of adiposity in hemorrhoids development. As far as we know, this is the first MR study to establish a causal relationship between adiposity and hemorrhoids.

Our results were in line with most previously published observational studies evaluating the relationship between obesity and hemorrhoids. These studies were carried out among Europeans (7, 8), Asians (9, 10), and Americans (25). Using data from the Dutch Health Interview Surveys, Seidell and colleagues reported a positive association between severe overweight (body mass index [BMI] 30.0–40.0 kg/m^2^) and hemorrhoids in women (7). Similarly, in a large Italian survey study of 72,284 participants (34,787 men and 37,497 women) aged 15 years and above, Negri et al. (8) found that the prevalence of hemorrhoids or varices was significantly associated with body weight (relative risk = 1.2 for obese men, 1.5 for women). In addition to these European studies, a Korean National Health and Nutrition Examination Survey (KNHANES) study including a total of 17,228 individuals aged ≥19 years identified a link between obesity and enhanced hemorrhoids risk [odds ratio (OR) = 1.13, 95% CI: 1.01–1.26] (9). This finding was supported by another Korean study of 194,620 adults (10). However, not all observational studies reported a positive relationship between adiposity and hemorrhoids. In a cross sectional study involving 2,813 participants who underwent a colonoscopy, Peery and colleagues did not find any associations of overweight (OR = 0.89, 95% CI: 0.72–1.09) and obese (OR = 0.86, 95% CI: 0.70–1.06) with hemorrhoids risk (11). Conventional statistical approaches including the Mantel–Haenszel procedure (26) and regression models were the main methods used in these observational studies for evaluating the relationship. Large sample sizes were applied in several studies such as the study of Negri et al. (8) and Hong et al. (10), which increased the statistical power for discovering a potential relationship. However, due to the limitations of study design and conventional statistical methods, these observational studies were vulnerable to biases including confounding and reverse causation. It was unclear if their observed association between adiposity and hemorrhoids was causal.

In our study, the causal effects of adiposity on hemorrhoids were estimated in univariable and multivariable MR analyses. The MR framework effectively minimized the risk of biases that were major concerns in the previously published observational studies. Unlike these studies that mainly used BMI for evaluation, we used a range of adiposity measures including BMI, body fat percentage, waist circumference, and waist-to-hip ratio. We took into account not only total adiposity (BMI) but also specific adiposity indicators. For instance, waist circumference and waist-to-hip ratio are indicators of central adiposity, while body fat percentage is a proxy for total body fatness. The inclusion of these adiposity indicators could provide more valuable information regarding the association between adiposity and hemorrhoids. Our univariable IVW analyses revealed significant causal effects of the four adiposity indicators on hemorrhoids risk, which was supported by several sensitivity analyses and leave-one-out analyses. The causal direction was confirmed by the Steiger directionality test. Further multivariable MR suggested that the effects of BMI, body fat percentage, waist circumference, and waist-to-hip ratio on hemorrhoids were independent of physical activity. Our results encouraged future clinical studies to focus on body weight and fat reduction in hemorrhoids prevention.

The association between adiposity and hemorrhoids may be attributed to a variety of potential biological mechanisms. Firstly, adiposity was found to be associated with increased intrabdominal pressure (18, 27), which could contribute to hemorrhoids development. In individuals with excess adiposity, enhanced intrabdominal pressure may lead to impaired venous return and engorgement of the venous structure in the distal rectum. These changes could promote hemorrhoids development. Secondly, adiposity has a link with chronic inflammation and oxidative stress (28, 29). Adiposity-associated inflammation and oxidative stress triggered the synthesis of oxygen radicals, reactive lipids, and proinflammatory cytokines including interleukin (IL)-6 and tumor necrosis factor (TNF)-ɑ, activated tissue remodeling proteins such as matrix metalloproteinases, induced C-reactive protein production, and promoted mitochondrial dysfunction and DNA damage (30–32). Complex interactions between adiposity, chronic inflammation, and oxidative stress may have harmful effects on the supporting tissues of the anal cushions, causing their impairments. Thirdly, low fiber intake might play a role. According to a number of epidemiological studies, obese individuals usually have less intake of fiber than the general population (33–35). There was evidence showing that a low-fiber diet might be implicated in hemorrhoids development, although the reported results were conflicting (36, 37).

Our MR study has certain drawbacks that need to be acknowledged. Firstly, given that our findings are based on GWAS data derived from individuals of European ancestry, it is necessary to exercise caution when attempting to extrapolate our results and conclusions to other ethnic populations. Secondly, since we only used summary statistics for estimation, we did not evaluate the causal association in males and females separately. In a cross-sectional study of healthy young and middle-aged adults, Hong et al. (10) found a positive association of overweight with hemorrhoidal disease only in women but not in men. However, Lee and colleagues observed that high BMI (≥25) was associated with self-reported hemorrhoids in both males and females (9). The study of Negri et al. (8) also revealed a link between high BMI (≥30) and hemorrhoids or varices in both men and women. Thirdly, we mainly evaluated the effects of adiposity during adulthood on hemorrhoids in this MR study. This was consistent with most observational studies. It would be valuable to assess the impact of excess childhood adiposity at hemorrhoids in future analyses. Fourthly, we were unable to investigate the effect of adiposity on the severity of hemorrhoids.

In summary, our comprehensive MR study provided evidence for causal effects of genetically determined BMI, body fat percentage, waist circumference, and waist-to-hip ratio on hemorrhoids. These findings encouraged future clinical investigations to focus on body weight and fat reduction in the prevention of hemorrhoids.

## Data availability statement

The analysis in this research relied on publicly available datasets, which can be accessed through the IEU Open GWAS Project (https://gwas.mrcieu.ac.uk/).

## Ethics statement

Due to the exclusive analysis of publicly available GWAS summary-level data, ethical approval was not applicable to this Mendelian randomization study.

## Author contributions

JH contributed to study concept and design, acquisition and interpretation of data, statistical analyses, and manuscript writing. YG and HQ assisted in data interpretation and reviewing the manuscript. YX supervised the study, contributed to funding acquisition, and assisted in reviewing the manuscript. All authors read and approved the final manuscript.

## Footnotes

^1^https://biobank.ctsu.ox.ac.uk/crystal/label.cgi?id=100010

## References

[1] Agarwal N Singh K Sheikh P Mittal K Mathai V Kumar A. Executive summary - the Association of Colon and rectal surgeons of India (ACRSI) practice guidelines for the management of hemorrhoids-2016. Indian J Surg. (2017) 79:58–61. doi: 10.1007/s12262-016-1578-7, PMID: 28331268 PMC5346092

[2] Kumar M Roy V Prasad S Jaiswal P Arun N Gopal K. Outcomes of rubber band ligation in hemorrhoids among outdoor patients. Cureus. (2022) 14:e29767. doi: 10.7759/cureus.29767 36324345 PMC9618009

[3] Lohsiriwat V. Approach to hemorrhoids. Curr Gastroenterol Rep. (2013) 15:332. doi: 10.1007/s11894-013-0332-6 23715885

[4] De Robles MS Young CJ. Surgical technique is the main predictor of recurrence in the management of hemorrhoids. ANZ J Surg. (2021) 91:1854–8. doi: 10.1111/ans.16738 33724701

[5] Brown SR. Hemorrhoids: an update on management. Ther Adv Chronic Dis. (2017) 8:141–7. doi: 10.1177/2040622317713957 28989595 PMC5624348

[6] Lohsiriwat V. Treatment of hemorrhoids: a coloproctologist’s view. World J Gastroenterol. (2015) 21:9245–52. doi: 10.3748/wjg.v21.i31.9245 26309351 PMC4541377

[7] Seidell JC de Groot LC van Sonsbeek JL Deurenberg P Hautvast JG. Associations of moderate and severe overweight with self-reported illness and medical care in Dutch adults. Am J Public Health. (1986) 76:264–9. doi: 10.2105/ajph.76.3.264 3946713 PMC1646554

[8] Negri E Pagano R Decarli A La Vecchia C. Body weight and the prevalence of chronic diseases. J Epidemiol Community Health. (1988) 42:24–9. doi: 10.1136/jech.42.1.24 3418282 PMC1052676

[9] Lee JH Kim HE Kang JH Shin JY Song YM. Factors associated with hemorrhoids in Korean adults: Korean national health and nutrition examination survey. Korean J Fam Med. (2014) 35:227–36. doi: 10.4082/kjfm.2014.35.5.227 25309703 PMC4192796

[10] Hong YS Jung KU Rampal S Zhao D Guallar E Ryu S. Risk factors for hemorrhoidal disease among healthy young and middle-aged Korean adults. Sci Rep. (2022) 12:129. doi: 10.1038/s41598-021-03838-z 34996957 PMC8741788

[11] Peery AF Sandler RS Galanko JA Bresalier RS Figueiredo JC Ahnen DJ. Risk factors for Hemorrhoids on screening colonoscopy. PLoS One. (2015) 10:e0139100. doi: 10.1371/journal.pone.0139100 26406337 PMC4583402

[12] Bennett DA Du H. An overview of methods and exemplars of the use of mendelian randomisation in nutritional research. Nutrients. (2022) 14:3408. doi: 10.3390/nu14163408 36014914 PMC9412324

[13] Boehm FJ Zhou X. Statistical methods for Mendelian randomization in genome-wide association studies: a review. Comput Struct Biotechnol J. (2022) 20:2338–51. doi: 10.1016/j.csbj.2022.05.015 35615025 PMC9123217

[14] Shungin D Winkler TW Croteau-Chonka DC Ferreira T Locke AE Mägi R. New genetic loci link adipose and insulin biology to body fat distribution. Nature. (2015) 518:187–96. doi: 10.1038/nature14132 25673412 PMC4338562

[15] 1000 Genomes Project Consortium Abecasis GR Auton A Brooks LD DePristo MA Durbin RM. An integrated map of genetic variation from 1,092 human genomes. Nature. (2012) 491:56–65. doi: 10.1038/nature11632 23128226 PMC3498066

[16] Hemani G Tilling K Davey SG. Orienting the causal relationship between imprecisely measured traits using GWAS summary data. PLoS Genet. (2017) 13:e1007081. doi: 10.1371/journal.pgen.1007081 29149188 PMC5711033

[17] Hemani G Zheng J Elsworth B Wade KH Haberland V Baird D. The MR-base platform supports systematic causal inference across the human phenome. Elife. (2018) 7:e34408. doi: 10.7554/eLife.34408 29846171 PMC5976434

[18] Zhao SS Holmes MV Zheng J Sanderson E Carter AR. The impact of education inequality on rheumatoid arthritis risk is mediated by smoking and body mass index: Mendelian randomization study. Rheumatology. (2022) 61:2167–75. doi: 10.1093/rheumatology/keab654 34436562 PMC9071527

[19] Burgess S Butterworth A Thompson SG. Mendelian randomization analysis with multiple genetic variants using summarized data. Genet Epidemiol. (2013) 37:658–65. doi: 10.1002/gepi.21758 24114802 PMC4377079

[20] Slob EAW Burgess S. A comparison of robust Mendelian randomization methods using summary data. Genet Epidemiol. (2020) 44:313–29. doi: 10.1002/gepi.22295 32249995 PMC7317850

[21] Soremekun O Karhunen V He Y Rajasundaram S Liu B Gkatzionis A. Lipid traits and type 2 diabetes risk in African ancestry individuals: a Mendelian randomization study. EBioMedicine. (2022) 78:103953. doi: 10.1016/j.ebiom.2022.103953 35325778 PMC8941323

[22] Bowden J Davey Smith G Burgess S. Mendelian randomization with invalid instruments: effect estimation and bias detection through egger regression. Int J Epidemiol. (2015) 44:512–25. doi: 10.1093/ije/dyv080 26050253 PMC4469799

[23] Verbanck M Chen CY Neale B Do R. Detection of widespread horizontal pleiotropy in causal relationships inferred from Mendelian randomization between complex traits and diseases. Nat Genet. (2018) 50:693–8. doi: 10.1038/s41588-018-0099-7 29686387 PMC6083837

[24] Kjaergaard AD Teumer A Marouli E Deloukas P Kuś A Sterenborg R. Thyroid function, pernicious anemia and erythropoiesis: a two-sample Mendelian randomization study. Hum Mol Genet. (2022) 31:2548–59. doi: 10.1093/hmg/ddac052 35225327

[25] Johanson JF Sonnenberg A. Constipation is not a risk factor for hemorrhoids: a case-control study of potential etiological agents. Am J Gastroenterol. (1994) 89:1981–6. 7942722

[26] Mantel N Haenszel W. Statistical aspects of the analysis of data from retrospective studies of disease. J Natl Cancer Inst. (1959) 22:719–48. 13655060

[27] Frezza EE Shebani KO Robertson J Wachtel MS. Morbid obesity causes chronic increase of intraabdominal pressure. Dig Dis Sci. (2007) 52:1038–41. doi: 10.1007/s10620-006-9203-4 17342401

[28] Hildebrandt X Ibrahim M Peltzer N. Cell death and inflammation during obesity: “know my methods, WAT(son)”. Cell Death Differ. (2023) 30:279–92. doi: 10.1038/s41418-022-01062-4 36175539 PMC9520110

[29] Świątkiewicz I Wróblewski M Nuszkiewicz J Sutkowy P Wróblewska J Woźniak A. The role of oxidative stress enhanced by adiposity in cardiometabolic diseases. Int J Mol Sci. (2023) 24:6382. doi: 10.3390/ijms24076382 37047352 PMC10094567

[30] Catalán V Gómez-Ambrosi J Rodríguez A Frühbeck G. Role of extracellular matrix remodelling in adipose tissue pathophysiology: relevance in the development of obesity. Histol Histopathol. (2012) 27:1515–28. doi: 10.14670/HH-27.1515 23059882

[31] Karaskova E Velganova-Veghova M Geryk M Foltenova H Kucerova V Karasek D. Role of adipose tissue in inflammatory bowel disease. Int J Mol Sci. (2021) 22:4226. doi: 10.3390/ijms22084226 33921758 PMC8073530

[32] Kessler C. Pathophysiology of obesity. Nurs Clin North Am. (2021) 56:465–78. doi: 10.1016/j.cnur.2021.08.001 34749888

[33] Araujo MC Estima CCP Yokoo EM Lopes TS Pereira RA Sichieri R. Are there differences in nutrient intake of Brazilian adults according to weight status? Cien Saude Colet. (2019) 24:2411–8. doi: 10.1590/1413-81232018247 31340260

[34] Song S Song Y. Dietary Fiber and its source are associated with cardiovascular risk factors in Korean adults. Nutrients. (2021) 13:160. doi: 10.3390/nu13010160 33419070 PMC7825439

[35] Waksmanska W Bobinski R Wos H Ilczak T. Amount of fibre in the diet with regard to excessive weight and obesity among children and adolescents in rural communities. J Nutr Sci Vitaminol. (2021) 67:189–95. doi: 10.3177/jnsv.67.189 34193678

[36] Brisinda G. How to treat hemorrhoids. Prevention is best; hemorrhoidectomy needs skilled operators. BMJ. (2000) 321:582–3. doi: 10.1136/bmj.321.7261.582 10977817 PMC1118483

[37] Lohsiriwat V. Hemorrhoids: from basic pathophysiology to clinical management. World J Gastroenterol. (2012) 18:2009–17. doi: 10.3748/wjg.v18.i17.2009 22563187 PMC3342598

