# Supplementary material for: Causal association between adiposity and hemorrhoids: a Mendelian randomization study
Source: Front Med (Lausanne). 2023 Oct 6;10:1229925. doi: 10.3389/fmed.2023.1229925 (PMC10587414; doi:10.3389/fmed.2023.1229925)
Supplement: Supplementary file 2 [file Table_2.docx]

Supplementary Table 2 Instrumental genetic variants for body mass index.

| rsID of SNP | Position | Chr | Allele | | Effect allele frequency | Association with body mass index | | |
| --- | --- | --- | --- | --- | --- | --- | --- | --- |
|  |  |  | EA | OA |  | Effect size (beta) | Standard error of beta | *P*-value |
| rs10063055 | 140990108 | 5 | T | C | 0.253 | 140990108 | 0.002 | 1.70E-09 |
| rs10099330 | 143383694 | 8 | G | A | 0.453 | 143383694 | 0.002 | 4.20E-10 |
| rs10160769 | 76474827 | 11 | C | G | 0.218 | 76474827 | 0.002 | 1.20E-10 |
| rs10169594 | 41637688 | 2 | C | T | 0.363 | 41637688 | 0.002 | 3.00E-09 |
| rs10182416 | 104242992 | 2 | G | A | 0.512 | 104242992 | 0.002 | 3.70E-11 |
| rs10423928 | 46182304 | 19 | A | T | 0.194 | 46182304 | 0.002 | 3.40E-42 |
| rs10505836 | 19288508 | 12 | C | A | 0.860 | 19288508 | 0.003 | 1.20E-10 |
| rs10510025 | 118650996 | 10 | T | C | 0.247 | 118650996 | 0.002 | 2.20E-14 |
| rs1064213 | 198950240 | 2 | A | G | 0.478 | 198950240 | 0.002 | 3.70E-14 |
| rs10742752 | 45438374 | 11 | C | T | 0.612 | 45438374 | 0.002 | 6.10E-09 |
| rs10756714 | 15885041 | 9 | G | A | 0.444 | 15885041 | 0.002 | 1.90E-25 |
| rs10756792 | 16726119 | 9 | T | C | 0.743 | 16726119 | 0.002 | 4.90E-17 |
| rs10760277 | 126093999 | 9 | T | C | 0.385 | 126093999 | 0.002 | 1.00E-11 |
| rs10780248 | 81370555 | 9 | A | G | 0.559 | 81370555 | 0.002 | 1.20E-09 |
| rs1078141 | 142619393 | 8 | T | C | 0.384 | 142619393 | 0.002 | 4.90E-12 |
| rs10799778 | 23313353 | 1 | G | T | 0.834 | 23313353 | 0.003 | 6.00E-12 |
| rs10809621 | 11859607 | 9 | G | C | 0.350 | 11859607 | 0.002 | 1.40E-09 |
| rs10824211 | 76363107 | 10 | T | C | 0.139 | 76363107 | 0.003 | 4.30E-13 |
| rs10832778 | 17394073 | 11 | G | C | 0.623 | 17394073 | 0.002 | 1.40E-08 |
| rs10927006 | 243557659 | 1 | C | T | 0.144 | 243557659 | 0.003 | 2.30E-09 |
| rs10965698 | 23203619 | 9 | T | C | 0.370 | 23203619 | 0.002 | 3.90E-08 |
| rs10989067 | 103119634 | 9 | A | G | 0.316 | 103119634 | 0.002 | 1.40E-15 |
| rs11001963 | 78760959 | 10 | T | C | 0.581 | 78760959 | 0.002 | 7.30E-09 |
| rs11009685 | 34511990 | 10 | T | C | 0.244 | 34511990 | 0.002 | 1.40E-08 |
| rs11012732 | 21830104 | 10 | G | A | 0.332 | 21830104 | 0.002 | 7.10E-25 |
| rs11079849 | 47090785 | 17 | T | C | 0.329 | 47090785 | 0.002 | 1.80E-21 |
| rs11099020 | 130724902 | 4 | T | C | 0.641 | 130724902 | 0.002 | 5.60E-12 |
| rs11115160 | 82424100 | 12 | A | G | 0.238 | 82424100 | 0.002 | 2.20E-08 |
| rs11122450 | 230301811 | 1 | G | T | 0.612 | 230301811 | 0.002 | 9.10E-09 |
| rs11134679 | 170623391 | 5 | G | A | 0.685 | 170623391 | 0.002 | 1.20E-17 |
| rs11150745 | 78757626 | 17 | G | A | 0.318 | 78757626 | 0.002 | 2.90E-23 |
| rs111598585 | 171635471 | 4 | T | C | 0.209 | 171635471 | 0.002 | 5.40E-09 |
| rs11165643 | 96924097 | 1 | T | C | 0.590 | 96924097 | 0.002 | 4.90E-22 |
| rs111689389 | 27175962 | 5 | C | G | 0.283 | 27175962 | 0.002 | 4.70E-10 |
| rs11218510 | 121922587 | 11 | A | G | 0.400 | 121922587 | 0.002 | 8.40E-13 |
| rs1126930 | 49399132 | 12 | C | G | 0.035 | 49399132 | 0.005 | 1.70E-09 |
| rs113079574 | 147354089 | 4 | T | C | 0.193 | 147354089 | 0.003 | 5.80E-10 |
| rs113603865 | 39564930 | 1 | T | C | 0.212 | 39564930 | 0.002 | 1.80E-14 |
| rs113624107 | 88326386 | 14 | A | G | 0.226 | 88326386 | 0.002 | 2.10E-10 |
| rs11525873 | 138817193 | 7 | C | T | 0.098 | 138817193 | 0.003 | 6.60E-13 |
| rs11607476 | 115037061 | 11 | C | A | 0.487 | 115037061 | 0.002 | 3.10E-15 |
| rs11610621 | 121671133 | 12 | A | T | 0.148 | 121671133 | 0.003 | 3.10E-09 |
| rs11630647 | 99240947 | 15 | A | G | 0.252 | 99240947 | 0.002 | 2.70E-08 |
| rs116374395 | 50723410 | 5 | A | G | 0.035 | 50723410 | 0.005 | 2.80E-09 |
| rs11642090 | 81730582 | 16 | C | T | 0.374 | 81730582 | 0.002 | 2.90E-08 |
| rs11656076 | 31464270 | 17 | A | G | 0.225 | 31464270 | 0.002 | 7.60E-11 |
| rs1167311 | 49996959 | 1 | A | G | 0.681 | 49996959 | 0.002 | 1.60E-19 |
| rs11675464 | 204053742 | 2 | G | A | 0.563 | 204053742 | 0.002 | 1.60E-09 |
| rs11691869 | 100805996 | 2 | A | C | 0.362 | 100805996 | 0.002 | 5.60E-21 |
| rs11699828 | 62157198 | 20 | A | G | 0.036 | 62157198 | 0.006 | 8.60E-09 |
| rs11709402 | 131551027 | 3 | G | A | 0.279 | 131551027 | 0.002 | 4.90E-25 |
| rs117118217 | 131783328 | 10 | C | G | 0.018 | 131783328 | 0.008 | 1.30E-08 |
| rs117342986 | 54267868 | 16 | T | C | 0.026 | 54267868 | 0.006 | 1.60E-08 |
| rs11757278 | 13180454 | 6 | C | T | 0.304 | 13180454 | 0.002 | 9.90E-12 |
| rs11778219 | 87762607 | 8 | G | A | 0.163 | 87762607 | 0.003 | 4.20E-09 |
| rs118136827 | 2168104 | 17 | T | G | 0.281 | 2168104 | 0.002 | 1.70E-09 |
| rs11919665 | 48085349 | 3 | T | A | 0.680 | 48085349 | 0.002 | 1.40E-09 |
| rs12001437 | 34074476 | 9 | C | T | 0.368 | 34074476 | 0.002 | 2.80E-09 |
| rs12072739 | 98315893 | 1 | G | A | 0.224 | 98315893 | 0.002 | 3.30E-11 |
| rs12088284 | 80798635 | 1 | T | C | 0.301 | 80798635 | 0.002 | 8.90E-11 |
| rs12089815 | 91189933 | 1 | A | G | 0.549 | 91189933 | 0.002 | 5.70E-10 |
| rs12140153 | 62579891 | 1 | T | G | 0.094 | 62579891 | 0.003 | 1.20E-21 |
| rs12149660 | 70309237 | 16 | A | G | 0.115 | 70309237 | 0.003 | 3.00E-13 |
| rs12259464 | 53680099 | 10 | A | G | 0.484 | 53680099 | 0.002 | 4.50E-11 |
| rs12273545 | 116911012 | 11 | T | C | 0.057 | 116911012 | 0.004 | 6.80E-09 |
| rs1229984 | 100239319 | 4 | C | T | 0.973 | 100239319 | 0.006 | 4.60E-10 |
| rs12364470 | 134601012 | 11 | G | T | 0.165 | 134601012 | 0.003 | 4.90E-13 |
| rs12440603 | 46585722 | 15 | T | C | 0.434 | 46585722 | 0.002 | 3.40E-12 |
| rs12459368 | 18459377 | 19 | G | A | 0.268 | 18459377 | 0.002 | 2.50E-14 |
| rs12462975 | 30272202 | 19 | A | G | 0.330 | 30272202 | 0.002 | 2.60E-20 |
| rs12541408 | 95585807 | 8 | C | T | 0.317 | 95585807 | 0.002 | 1.60E-11 |
| rs1266874 | 51779638 | 6 | G | A | 0.350 | 51779638 | 0.002 | 1.00E-11 |
| rs12681792 | 62054463 | 8 | A | C | 0.193 | 62054463 | 0.003 | 3.50E-09 |
| rs12692596 | 161265910 | 2 | T | C | 0.372 | 161265910 | 0.002 | 1.30E-10 |
| rs12696039 | 156304750 | 3 | G | A | 0.149 | 156304750 | 0.003 | 3.50E-08 |
| rs1286058 | 91458523 | 14 | A | T | 0.704 | 91458523 | 0.002 | 6.20E-12 |
| rs12881629 | 101146413 | 14 | G | A | 0.083 | 101146413 | 0.004 | 7.70E-10 |
| rs12921986 | 72312727 | 16 | G | A | 0.078 | 72312727 | 0.004 | 3.80E-08 |
| rs12937411 | 34950239 | 17 | T | C | 0.408 | 34950239 | 0.002 | 1.40E-17 |
| rs1296328 | 137083193 | 4 | C | A | 0.559 | 137083193 | 0.002 | 3.90E-21 |
| rs12974458 | 1866115 | 19 | T | C | 0.543 | 1866115 | 0.002 | 2.30E-14 |
| rs13012070 | 35447243 | 2 | A | G | 0.228 | 35447243 | 0.002 | 6.10E-09 |
| rs13033310 | 133523605 | 2 | A | G | 0.253 | 133523605 | 0.002 | 3.30E-08 |
| rs13097918 | 35676330 | 3 | A | T | 0.212 | 35676330 | 0.002 | 1.70E-09 |
| rs13107325 | 103188709 | 4 | T | C | 0.075 | 103188709 | 0.004 | 8.50E-37 |
| rs13176429 | 43152216 | 5 | C | T | 0.688 | 43152216 | 0.002 | 3.10E-11 |
| rs1320251 | 21264396 | 17 | T | C | 0.455 | 21264396 | 0.002 | 1.50E-19 |
| rs13218383 | 120173501 | 6 | G | C | 0.335 | 120173501 | 0.002 | 5.80E-12 |
| rs1322842 | 20488897 | 6 | G | A | 0.609 | 20488897 | 0.002 | 1.10E-10 |
| rs13248187 | 14336834 | 8 | C | T | 0.269 | 14336834 | 0.002 | 2.00E-12 |
| rs1327259 | 51177811 | 6 | G | A | 0.388 | 51177811 | 0.002 | 2.80E-13 |
| rs13291723 | 80510077 | 9 | A | G | 0.571 | 80510077 | 0.002 | 3.40E-08 |
| rs1330199 | 27760946 | 9 | T | G | 0.483 | 27760946 | 0.002 | 3.30E-09 |
| rs13420048 | 50751414 | 2 | A | C | 0.365 | 50751414 | 0.002 | 4.60E-14 |
| rs13427822 | 213414265 | 2 | G | A | 0.271 | 213414265 | 0.002 | 5.60E-16 |
| rs1346841 | 65651730 | 4 | A | G | 0.405 | 65651730 | 0.002 | 9.70E-11 |
| rs1360201 | 73796450 | 9 | T | C | 0.482 | 73796450 | 0.002 | 4.80E-11 |
| rs13642 | 30432220 | 11 | T | A | 0.361 | 30432220 | 0.002 | 4.30E-15 |
| rs140159717 | 73765586 | 15 | T | C | 0.082 | 73765586 | 0.004 | 2.80E-11 |
| rs1438945 | 152510937 | 5 | A | T | 0.715 | 152510937 | 0.002 | 1.10E-09 |
| rs1441264 | 79580919 | 13 | A | G | 0.594 | 79580919 | 0.002 | 3.40E-18 |
| rs1451963 | 41350367 | 14 | T | G | 0.082 | 41350367 | 0.004 | 7.40E-10 |
| rs1458156 | 41887940 | 12 | T | C | 0.488 | 41887940 | 0.002 | 1.20E-12 |
| rs145981104 | 74714869 | 8 | G | A | 0.064 | 74714869 | 0.004 | 2.00E-08 |
| rs146569428 | 2199686 | 11 | A | G | 0.201 | 2199686 | 0.002 | 2.00E-08 |
| rs1471093 | 108031094 | 3 | A | G | 0.617 | 108031094 | 0.002 | 4.10E-11 |
| rs1471740 | 136328270 | 3 | C | T | 0.741 | 136328270 | 0.002 | 8.90E-18 |
| rs147568678 | 93061851 | 10 | C | T | 0.238 | 93061851 | 0.002 | 1.10E-08 |
| rs1477290 | 87988934 | 5 | C | T | 0.137 | 87988934 | 0.003 | 2.20E-31 |
| rs147730268 | 123024476 | 12 | T | G | 0.087 | 123024476 | 0.004 | 1.30E-22 |
| rs1503526 | 63020706 | 5 | C | T | 0.480 | 63020706 | 0.002 | 5.90E-15 |
| rs156201 | 104847441 | 6 | C | G | 0.753 | 104847441 | 0.002 | 8.30E-09 |
| rs156914 | 16848652 | 1 | A | G | 0.492 | 16848652 | 0.002 | 1.60E-08 |
| rs1582931 | 122657199 | 5 | A | G | 0.473 | 122657199 | 0.002 | 2.30E-11 |
| rs1608113 | 157815217 | 3 | T | A | 0.365 | 157815217 | 0.002 | 9.30E-09 |
| rs1609010 | 77227464 | 8 | G | A | 0.566 | 77227464 | 0.002 | 7.90E-26 |
| rs16916303 | 30823761 | 9 | G | A | 0.120 | 30823761 | 0.003 | 4.20E-10 |
| rs17056301 | 158271680 | 5 | C | T | 0.256 | 158271680 | 0.002 | 2.20E-09 |
| rs17132130 | 2108036 | 7 | C | G | 0.221 | 2108036 | 0.002 | 7.50E-14 |
| rs17149254 | 76634463 | 7 | C | T | 0.805 | 76634463 | 0.003 | 7.00E-17 |
| rs17289010 | 140774684 | 4 | G | A | 0.328 | 140774684 | 0.002 | 1.60E-10 |
| rs17399739 | 87490850 | 10 | G | A | 0.069 | 87490850 | 0.004 | 4.40E-12 |
| rs17446299 | 40762556 | 13 | G | C | 0.166 | 40762556 | 0.003 | 8.90E-09 |
| rs17544384 | 115295160 | 1 | C | T | 0.211 | 115295160 | 0.002 | 5.30E-09 |
| rs17668356 | 61208619 | 3 | G | C | 0.146 | 61208619 | 0.003 | 1.50E-16 |
| rs17770336 | 28414625 | 9 | T | C | 0.322 | 28414625 | 0.002 | 1.30E-30 |
| rs1778830 | 156489974 | 1 | A | G | 0.362 | 156489974 | 0.002 | 7.50E-12 |
| rs1788808 | 21090023 | 18 | G | A | 0.495 | 21090023 | 0.002 | 7.70E-25 |
| rs1793636 | 131934926 | 11 | C | G | 0.309 | 131934926 | 0.002 | 4.70E-10 |
| rs1805123 | 150645534 | 7 | G | T | 0.245 | 150645534 | 0.002 | 3.00E-13 |
| rs1834144 | 40744790 | 18 | A | C | 0.373 | 40744790 | 0.002 | 8.60E-12 |
| rs1861410 | 58933591 | 2 | T | C | 0.555 | 58933591 | 0.002 | 1.20E-26 |
| rs1884897 | 6612832 | 20 | G | A | 0.627 | 6612832 | 0.002 | 2.30E-22 |
| rs1919243 | 88778861 | 5 | C | T | 0.487 | 88778861 | 0.002 | 5.50E-09 |
| rs1967772 | 28036062 | 13 | A | G | 0.285 | 28036062 | 0.002 | 1.00E-14 |
| rs2035936 | 141298124 | 3 | T | G | 0.056 | 141298124 | 0.004 | 1.70E-17 |
| rs2051559 | 3298800 | 4 | C | T | 0.133 | 3298800 | 0.003 | 2.80E-12 |
| rs2075466 | 4872970 | 16 | C | G | 0.267 | 4872970 | 0.002 | 2.80E-09 |
| rs2102278 | 52818664 | 4 | G | A | 0.322 | 52818664 | 0.002 | 2.00E-08 |
| rs2133561 | 139086651 | 5 | T | A | 0.611 | 139086651 | 0.002 | 5.80E-12 |
| rs213518 | 26941065 | 7 | C | T | 0.146 | 26941065 | 0.003 | 1.80E-08 |
| rs2153740 | 2126089 | 20 | G | A | 0.480 | 2126089 | 0.002 | 1.60E-08 |
| rs215634 | 32369148 | 7 | G | A | 0.612 | 32369148 | 0.002 | 2.40E-14 |
| rs2172131 | 133978962 | 10 | C | T | 0.579 | 133978962 | 0.002 | 9.00E-14 |
| rs217672 | 62361021 | 14 | C | A | 0.272 | 62361021 | 0.002 | 2.40E-14 |
| rs2192158 | 55505360 | 4 | G | A | 0.553 | 55505360 | 0.002 | 3.70E-14 |
| rs2216931 | 181599070 | 2 | A | C | 0.662 | 181599070 | 0.002 | 5.20E-16 |
| rs2234458 | 65639374 | 11 | T | C | 0.640 | 65639374 | 0.002 | 3.60E-23 |
| rs2248551 | 131924689 | 6 | A | G | 0.165 | 131924689 | 0.003 | 3.80E-08 |
| rs2253310 | 108888593 | 6 | G | C | 0.626 | 108888593 | 0.002 | 2.10E-17 |
| rs2271189 | 56494991 | 12 | A | G | 0.403 | 56494991 | 0.002 | 6.50E-16 |
| rs2289379 | 44804225 | 7 | T | C | 0.396 | 44804225 | 0.002 | 5.20E-14 |
| rs2307111 | 75003678 | 5 | C | T | 0.395 | 75003678 | 0.002 | 1.30E-43 |
| rs2342892 | 24540806 | 16 | G | T | 0.516 | 24540806 | 0.002 | 1.30E-10 |
| rs2381404 | 144035442 | 2 | C | T | 0.244 | 144035442 | 0.002 | 1.30E-09 |
| rs2383377 | 33257914 | 14 | A | G | 0.131 | 33257914 | 0.003 | 3.90E-08 |
| rs2398861 | 96430747 | 9 | G | A | 0.259 | 96430747 | 0.002 | 2.10E-15 |
| rs2425816 | 44895075 | 20 | A | G | 0.415 | 44895075 | 0.002 | 1.20E-09 |
| rs2433733 | 230816703 | 2 | A | G | 0.678 | 230816703 | 0.002 | 3.80E-16 |
| rs2439823 | 99778226 | 10 | G | A | 0.546 | 99778226 | 0.002 | 5.30E-22 |
| rs2482356 | 94178371 | 9 | C | T | 0.429 | 94178371 | 0.002 | 1.30E-08 |
| rs2512892 | 131451862 | 11 | C | T | 0.566 | 131451862 | 0.002 | 9.20E-11 |
| rs252761 | 77380723 | 5 | T | G | 0.588 | 77380723 | 0.002 | 1.20E-08 |
| rs2568958 | 72765116 | 1 | A | G | 0.604 | 72765116 | 0.002 | 1.60E-28 |
| rs2569993 | 12926096 | 3 | C | T | 0.320 | 12926096 | 0.002 | 2.40E-09 |
| rs2606228 | 183537759 | 3 | C | A | 0.646 | 183537759 | 0.002 | 2.70E-11 |
| rs2616143 | 20632022 | 8 | A | G | 0.320 | 20632022 | 0.002 | 6.70E-11 |
| rs2618039 | 112324111 | 1 | T | A | 0.381 | 112324111 | 0.002 | 1.40E-12 |
| rs2678204 | 201800511 | 1 | G | T | 0.340 | 201800511 | 0.002 | 3.80E-31 |
| rs2725371 | 30854033 | 8 | G | A | 0.696 | 30854033 | 0.002 | 1.10E-13 |
| rs2791643 | 11207269 | 1 | T | C | 0.762 | 11207269 | 0.002 | 7.20E-09 |
| rs28350 | 42418446 | 3 | G | A | 0.821 | 42418446 | 0.003 | 2.90E-12 |
| rs28366156 | 31671498 | 6 | C | T | 0.131 | 31671498 | 0.003 | 1.60E-19 |
| rs2837996 | 42626706 | 21 | C | T | 0.651 | 42626706 | 0.002 | 1.10E-09 |
| rs28404639 | 80874229 | 5 | T | C | 0.366 | 80874229 | 0.002 | 1.20E-08 |
| rs28489620 | 41804716 | 22 | A | G | 0.290 | 41804716 | 0.002 | 2.80E-12 |
| rs28568418 | 53462969 | 15 | A | G | 0.108 | 53462969 | 0.003 | 1.20E-08 |
| rs2861685 | 67837553 | 2 | C | T | 0.412 | 67837553 | 0.002 | 1.00E-17 |
| rs28670671 | 140363045 | 9 | C | T | 0.286 | 140363045 | 0.002 | 3.70E-08 |
| rs2870111 | 79403585 | 15 | T | C | 0.412 | 79403585 | 0.002 | 7.10E-15 |
| rs2875762 | 124925032 | 6 | C | G | 0.243 | 124925032 | 0.002 | 3.30E-11 |
| rs2899644 | 59470366 | 15 | T | C | 0.230 | 59470366 | 0.002 | 2.30E-10 |
| rs2920503 | 12324230 | 3 | T | C | 0.285 | 12324230 | 0.002 | 1.70E-10 |
| rs2962334 | 86879056 | 5 | T | G | 0.020 | 86879056 | 0.007 | 7.80E-10 |
| rs317656 | 69681101 | 12 | A | T | 0.724 | 69681101 | 0.002 | 6.10E-11 |
| rs3213943 | 136389840 | 2 | A | C | 0.132 | 136389840 | 0.003 | 4.80E-10 |
| rs32421 | 167362416 | 5 | T | A | 0.224 | 167362416 | 0.002 | 2.60E-08 |
| rs329118 | 133861663 | 5 | T | C | 0.419 | 133861663 | 0.002 | 1.30E-16 |
| rs329651 | 133767622 | 11 | T | G | 0.804 | 133767622 | 0.003 | 3.30E-10 |
| rs34045288 | 40369081 | 6 | T | C | 0.334 | 40369081 | 0.002 | 3.50E-29 |
| rs34153025 | 41339697 | 15 | C | T | 0.022 | 41339697 | 0.007 | 9.60E-09 |
| rs34234296 | 175166636 | 2 | A | G | 0.392 | 175166636 | 0.002 | 2.40E-13 |
| rs34481751 | 47501038 | 20 | A | C | 0.165 | 47501038 | 0.003 | 7.80E-12 |
| rs34517439 | 78450517 | 1 | A | C | 0.122 | 78450517 | 0.003 | 3.60E-37 |
| rs34696181 | 93096635 | 7 | C | T | 0.476 | 93096635 | 0.002 | 8.10E-09 |
| rs34811474 | 25408838 | 4 | A | G | 0.231 | 25408838 | 0.002 | 4.10E-34 |
| rs349071 | 84776849 | 11 | A | G | 0.500 | 84776849 | 0.002 | 1.90E-11 |
| rs35154326 | 24862414 | 16 | G | A | 0.274 | 24862414 | 0.002 | 5.30E-09 |
| rs35364449 | 74278126 | 15 | T | C | 0.110 | 74278126 | 0.003 | 9.20E-12 |
| rs355777 | 154034950 | 3 | C | G | 0.408 | 154034950 | 0.002 | 3.30E-14 |
| rs35697587 | 47298505 | 14 | A | G | 0.508 | 47298505 | 0.002 | 9.20E-17 |
| rs35697691 | 52353498 | 15 | G | C | 0.089 | 52353498 | 0.004 | 6.20E-11 |
| rs35809007 | 47019521 | 2 | A | G | 0.363 | 47019521 | 0.002 | 9.10E-17 |
| rs35957544 | 73440371 | 8 | T | G | 0.574 | 73440371 | 0.002 | 1.10E-22 |
| rs36007635 | 163009335 | 6 | A | G | 0.138 | 163009335 | 0.003 | 2.20E-13 |
| rs36061954 | 38329650 | 8 | T | C | 0.399 | 38329650 | 0.002 | 2.00E-10 |
| rs3764625 | 49649051 | 19 | G | T | 0.588 | 49649051 | 0.002 | 5.20E-09 |
| rs3784710 | 68072458 | 15 | C | T | 0.227 | 68072458 | 0.002 | 2.50E-36 |
| rs3803286 | 103246470 | 14 | G | A | 0.667 | 103246470 | 0.002 | 6.40E-19 |
| rs3807566 | 50564204 | 7 | T | G | 0.438 | 50564204 | 0.002 | 1.50E-09 |
| rs3814883 | 29994922 | 16 | T | C | 0.482 | 29994922 | 0.002 | 1.00E-33 |
| rs3845344 | 75001480 | 1 | T | C | 0.391 | 75001480 | 0.002 | 5.40E-16 |
| rs3851998 | 131876605 | 3 | G | C | 0.743 | 131876605 | 0.002 | 2.00E-09 |
| rs3866805 | 6657424 | 1 | A | C | 0.356 | 6657424 | 0.002 | 1.20E-08 |
| rs3897102 | 123492112 | 12 | T | C | 0.411 | 123492112 | 0.002 | 2.50E-09 |
| rs3901286 | 99107727 | 7 | A | C | 0.152 | 99107727 | 0.003 | 2.70E-16 |
| rs3902951 | 69789755 | 14 | G | T | 0.237 | 69789755 | 0.002 | 2.00E-09 |
| rs3935190 | 79084367 | 17 | A | G | 0.537 | 79084367 | 0.002 | 4.00E-13 |
| rs394608 | 46581798 | 21 | C | T | 0.538 | 46581798 | 0.002 | 9.50E-21 |
| rs40071 | 107496102 | 5 | C | T | 0.180 | 107496102 | 0.003 | 3.80E-24 |
| rs4017425 | 44028764 | 3 | T | C | 0.470 | 44028764 | 0.002 | 2.10E-10 |
| rs4055791 | 59266053 | 13 | T | C | 0.417 | 59266053 | 0.002 | 7.40E-19 |
| rs406388 | 18226997 | 22 | G | C | 0.177 | 18226997 | 0.003 | 8.30E-10 |
| rs41279738 | 110082551 | 1 | G | T | 0.026 | 110082551 | 0.006 | 4.00E-28 |
| rs4148155 | 89054667 | 4 | G | A | 0.113 | 89054667 | 0.003 | 1.40E-13 |
| rs4261944 | 31003636 | 4 | G | T | 0.365 | 31003636 | 0.002 | 1.60E-11 |
| rs4267103 | 60966740 | 12 | C | T | 0.186 | 60966740 | 0.003 | 1.40E-09 |
| rs4284600 | 31843528 | 15 | C | T | 0.467 | 31843528 | 0.002 | 2.00E-09 |
| rs429343 | 147903382 | 2 | G | A | 0.577 | 147903382 | 0.002 | 3.30E-18 |
| rs429358 | 45411941 | 19 | C | T | 0.154 | 45411941 | 0.003 | 2.40E-22 |
| rs4307239 | 24354300 | 7 | G | A | 0.459 | 24354300 | 0.002 | 1.00E-09 |
| rs4419475 | 96150044 | 4 | T | A | 0.407 | 96150044 | 0.002 | 1.10E-08 |
| rs4444317 | 92573234 | 15 | G | A | 0.216 | 92573234 | 0.002 | 2.50E-11 |
| rs4456769 | 25190777 | 20 | T | C | 0.333 | 25190777 | 0.002 | 3.60E-12 |
| rs4477562 | 54104968 | 13 | T | C | 0.129 | 54104968 | 0.003 | 2.90E-23 |
| rs4482463 | 205375909 | 2 | A | C | 0.923 | 205375909 | 0.004 | 3.00E-17 |
| rs45486197 | 2244849 | 19 | A | G | 0.066 | 2244849 | 0.004 | 1.70E-10 |
| rs4605363 | 229010960 | 2 | C | A | 0.342 | 229010960 | 0.002 | 3.30E-15 |
| rs4648450 | 2723214 | 1 | A | C | 0.467 | 2723214 | 0.002 | 8.40E-14 |
| rs4658403 | 243832560 | 1 | T | C | 0.834 | 243832560 | 0.003 | 1.00E-12 |
| rs4672338 | 60217457 | 2 | T | C | 0.336 | 60217457 | 0.002 | 8.00E-11 |
| rs4722398 | 3125220 | 7 | T | C | 0.136 | 3125220 | 0.003 | 7.90E-11 |
| rs4764949 | 103658096 | 12 | G | A | 0.326 | 103658096 | 0.002 | 3.10E-18 |
| rs4790292 | 1824305 | 17 | A | C | 0.154 | 1824305 | 0.003 | 2.60E-20 |
| rs4820410 | 40690385 | 22 | G | A | 0.345 | 40690385 | 0.002 | 1.70E-17 |
| rs4832298 | 86764004 | 2 | T | C | 0.686 | 86764004 | 0.002 | 5.40E-14 |
| rs4858940 | 88254820 | 3 | C | T | 0.886 | 88254820 | 0.003 | 1.40E-13 |
| rs4876611 | 116671848 | 8 | G | A | 0.720 | 116671848 | 0.002 | 3.30E-19 |
| rs4929923 | 8639200 | 11 | C | T | 0.645 | 8639200 | 0.002 | 4.50E-20 |
| rs5011579 | 69187318 | 16 | G | C | 0.715 | 69187318 | 0.002 | 1.60E-10 |
| rs512121 | 7548501 | 18 | C | T | 0.192 | 7548501 | 0.003 | 2.60E-10 |
| rs529200 | 173114305 | 3 | G | A | 0.528 | 173114305 | 0.002 | 1.30E-17 |
| rs539515 | 177889025 | 1 | C | A | 0.205 | 177889025 | 0.002 | 2.00E-91 |
| rs55707359 | 46159333 | 11 | G | T | 0.015 | 46159333 | 0.008 | 6.20E-11 |
| rs55714539 | 18207397 | 19 | C | A | 0.344 | 18207397 | 0.002 | 6.00E-17 |
| rs55726687 | 991306 | 12 | A | G | 0.210 | 991306 | 0.002 | 1.40E-24 |
| rs55769038 | 13331808 | 11 | A | G | 0.590 | 13331808 | 0.002 | 1.20E-15 |
| rs558887 | 28712741 | 11 | G | A | 0.308 | 28712741 | 0.002 | 1.50E-09 |
| rs559231 | 39644247 | 18 | T | G | 0.393 | 39644247 | 0.002 | 3.40E-11 |
| rs56038322 | 69925128 | 3 | A | G | 0.311 | 69925128 | 0.002 | 9.00E-11 |
| rs56094641 | 53806453 | 16 | G | A | 0.405 | 53806453 | 0.002 | 1.00E-200 |
| rs56133507 | 172818467 | 2 | G | T | 0.197 | 172818467 | 0.002 | 2.70E-08 |
| rs56143236 | 157020444 | 3 | T | C | 0.256 | 157020444 | 0.002 | 2.30E-08 |
| rs56161855 | 46288649 | 17 | T | A | 0.133 | 46288649 | 0.003 | 1.40E-14 |
| rs56203622 | 131040874 | 9 | C | T | 0.146 | 131040874 | 0.003 | 1.40E-10 |
| rs56352336 | 19352155 | 19 | C | T | 0.155 | 19352155 | 0.003 | 2.90E-09 |
| rs56399737 | 33381721 | 13 | T | C | 0.449 | 33381721 | 0.002 | 6.40E-16 |
| rs56858768 | 86511730 | 13 | A | G | 0.297 | 86511730 | 0.002 | 2.40E-13 |
| rs56893062 | 25662655 | 8 | G | T | 0.303 | 25662655 | 0.002 | 6.20E-09 |
| rs56930105 | 10982487 | 2 | T | C | 0.139 | 10982487 | 0.003 | 3.70E-08 |
| rs57636386 | 58048295 | 18 | C | T | 0.084 | 58048295 | 0.004 | 1.10E-30 |
| rs57989773 | 100629078 | 6 | C | T | 0.245 | 100629078 | 0.002 | 1.60E-08 |
| rs58862095 | 75081418 | 7 | T | C | 0.419 | 75081418 | 0.002 | 2.40E-30 |
| rs59068084 | 113256737 | 4 | T | G | 0.410 | 113256737 | 0.002 | 3.80E-08 |
| rs59227842 | 43692423 | 11 | G | A | 0.311 | 43692423 | 0.002 | 1.50E-26 |
| rs594024 | 69443822 | 11 | C | T | 0.554 | 69443822 | 0.002 | 1.70E-13 |
| rs6023655 | 53479658 | 20 | G | A | 0.766 | 53479658 | 0.002 | 3.60E-10 |
| rs60764613 | 1839911 | 18 | T | G | 0.145 | 1839911 | 0.003 | 1.10E-13 |
| rs61740466 | 19934900 | 1 | A | G | 0.237 | 19934900 | 0.002 | 5.60E-09 |
| rs61813324 | 156049877 | 1 | T | C | 0.136 | 156049877 | 0.003 | 2.80E-23 |
| rs61828641 | 174321997 | 1 | A | G | 0.109 | 174321997 | 0.003 | 1.20E-12 |
| rs61871615 | 102487140 | 10 | T | C | 0.092 | 102487140 | 0.004 | 1.00E-13 |
| rs61903695 | 89922417 | 11 | G | A | 0.255 | 89922417 | 0.002 | 2.50E-13 |
| rs61992671 | 101531854 | 14 | G | A | 0.492 | 101531854 | 0.002 | 5.10E-15 |
| rs62007782 | 78029797 | 15 | A | G | 0.265 | 78029797 | 0.002 | 9.40E-14 |
| rs62020775 | 89960286 | 15 | A | T | 0.142 | 89960286 | 0.003 | 7.20E-09 |
| rs62072006 | 52938468 | 17 | C | A | 0.145 | 52938468 | 0.003 | 3.00E-08 |
| rs62107261 | 422144 | 2 | C | T | 0.048 | 422144 | 0.005 | 4.60E-87 |
| rs62176243 | 166190881 | 2 | T | A | 0.245 | 166190881 | 0.002 | 6.10E-11 |
| rs62190049 | 182566998 | 2 | C | G | 0.390 | 182566998 | 0.002 | 4.00E-08 |
| rs62241847 | 20466465 | 3 | G | A | 0.314 | 20466465 | 0.002 | 5.30E-09 |
| rs62246311 | 9498143 | 3 | A | G | 0.102 | 9498143 | 0.003 | 1.80E-10 |
| rs62379271 | 105870033 | 5 | G | T | 0.579 | 105870033 | 0.002 | 5.00E-09 |
| rs62407562 | 33530346 | 6 | A | T | 0.269 | 33530346 | 0.002 | 8.50E-11 |
| rs6265 | 27679916 | 11 | T | C | 0.188 | 27679916 | 0.003 | 3.30E-56 |
| rs6430068 | 145627927 | 2 | A | G | 0.109 | 145627927 | 0.003 | 5.30E-09 |
| rs6444950 | 170602073 | 3 | A | G | 0.237 | 170602073 | 0.002 | 8.30E-12 |
| rs6545714 | 59307725 | 2 | A | G | 0.601 | 59307725 | 0.002 | 2.40E-24 |
| rs6560906 | 133414054 | 12 | C | T | 0.692 | 133414054 | 0.002 | 1.20E-08 |
| rs6561937 | 58257667 | 13 | A | T | 0.754 | 58257667 | 0.002 | 4.60E-12 |
| rs6567160 | 57829135 | 18 | C | T | 0.233 | 57829135 | 0.002 | 2.30E-118 |
| rs6575340 | 94023972 | 14 | A | G | 0.636 | 94023972 | 0.002 | 8.70E-24 |
| rs66679256 | 18351898 | 4 | T | C | 0.446 | 18351898 | 0.002 | 6.90E-14 |
| rs6669341 | 47678458 | 1 | G | A | 0.583 | 47678458 | 0.002 | 1.60E-17 |
| rs6682438 | 33784146 | 1 | C | T | 0.673 | 33784146 | 0.002 | 3.70E-10 |
| rs6705567 | 55320173 | 2 | C | T | 0.376 | 55320173 | 0.002 | 9.80E-13 |
| rs6707827 | 100123030 | 2 | G | A | 0.704 | 100123030 | 0.002 | 3.90E-08 |
| rs6710091 | 239597 | 2 | G | C | 0.348 | 239597 | 0.002 | 1.60E-08 |
| rs6713781 | 40291940 | 2 | C | G | 0.402 | 40291940 | 0.002 | 2.20E-11 |
| rs6725931 | 220205146 | 2 | T | C | 0.848 | 220205146 | 0.003 | 3.30E-12 |
| rs6744646 | 628504 | 2 | G | A | 0.828 | 628504 | 0.003 | 4.50E-100 |
| rs6752979 | 81741750 | 2 | A | G | 0.317 | 81741750 | 0.002 | 3.30E-09 |
| rs67609008 | 126640936 | 10 | C | T | 0.284 | 126640936 | 0.002 | 8.60E-15 |
| rs6769617 | 62687746 | 3 | T | A | 0.664 | 62687746 | 0.002 | 8.10E-11 |
| rs6774894 | 196116393 | 3 | A | T | 0.358 | 196116393 | 0.002 | 9.70E-11 |
| rs6777784 | 62376645 | 3 | T | G | 0.617 | 62376645 | 0.002 | 1.00E-08 |
| rs6831088 | 20257769 | 4 | A | G | 0.640 | 20257769 | 0.002 | 2.20E-08 |
| rs6843852 | 162132758 | 4 | T | C | 0.508 | 162132758 | 0.002 | 3.50E-11 |
| rs6909685 | 97753952 | 6 | T | C | 0.327 | 97753952 | 0.002 | 4.70E-12 |
| rs6922607 | 142703483 | 6 | G | A | 0.190 | 142703483 | 0.003 | 3.20E-09 |
| rs6938973 | 98421721 | 6 | C | T | 0.601 | 98421721 | 0.002 | 1.80E-19 |
| rs6950388 | 1270699 | 7 | A | G | 0.795 | 1270699 | 0.002 | 2.30E-10 |
| rs6962980 | 113452183 | 7 | C | A | 0.556 | 113452183 | 0.002 | 1.00E-15 |
| rs698147 | 3513485 | 5 | G | A | 0.544 | 3513485 | 0.002 | 9.60E-11 |
| rs7024334 | 109072075 | 9 | G | T | 0.779 | 109072075 | 0.002 | 6.70E-09 |
| rs7027304 | 129408290 | 9 | T | C | 0.653 | 129408290 | 0.002 | 3.10E-12 |
| rs7034554 | 37081301 | 9 | G | A | 0.374 | 37081301 | 0.002 | 2.10E-10 |
| rs7038943 | 120377178 | 9 | C | T | 0.339 | 120377178 | 0.002 | 1.80E-11 |
| rs704061 | 89771903 | 12 | C | T | 0.455 | 89771903 | 0.002 | 1.70E-13 |
| rs7070670 | 61842645 | 10 | T | C | 0.328 | 61842645 | 0.002 | 6.00E-09 |
| rs7081254 | 132955696 | 10 | C | T | 0.206 | 132955696 | 0.002 | 6.20E-09 |
| rs7124681 | 47529947 | 11 | A | C | 0.408 | 47529947 | 0.002 | 1.50E-37 |
| rs7132908 | 50263148 | 12 | A | G | 0.384 | 50263148 | 0.002 | 1.40E-48 |
| rs71495038 | 33971383 | 10 | A | G | 0.077 | 33971383 | 0.004 | 6.70E-14 |
| rs7201895 | 407723 | 16 | A | G | 0.354 | 407723 | 0.002 | 6.00E-13 |
| rs7206608 | 82872628 | 16 | G | C | 0.322 | 82872628 | 0.002 | 1.80E-10 |
| rs7218014 | 65832016 | 17 | C | T | 0.197 | 65832016 | 0.002 | 2.90E-14 |
| rs7232171 | 31251221 | 18 | T | G | 0.583 | 31251221 | 0.002 | 8.00E-10 |
| rs723672 | 2161561 | 12 | T | C | 0.432 | 2161561 | 0.002 | 3.00E-08 |
| rs7250833 | 33937277 | 19 | T | C | 0.289 | 33937277 | 0.002 | 6.70E-10 |
| rs7259070 | 47562509 | 19 | C | T | 0.596 | 47562509 | 0.002 | 6.60E-27 |
| rs72634826 | 1601052 | 1 | A | G | 0.260 | 1601052 | 0.002 | 1.00E-20 |
| rs72649373 | 80609966 | 4 | C | T | 0.143 | 80609966 | 0.003 | 6.10E-10 |
| rs72673947 | 118884379 | 8 | G | A | 0.107 | 118884379 | 0.003 | 9.90E-12 |
| rs72892910 | 50816887 | 6 | T | G | 0.172 | 50816887 | 0.003 | 1.50E-49 |
| rs72976986 | 4050424 | 19 | A | G | 0.190 | 4050424 | 0.003 | 7.60E-20 |
| rs73026725 | 31017686 | 19 | A | C | 0.154 | 31017686 | 0.003 | 4.90E-16 |
| rs73052033 | 185828465 | 3 | C | T | 0.185 | 185828465 | 0.003 | 7.70E-33 |
| rs7306534 | 68107914 | 12 | A | G | 0.622 | 68107914 | 0.002 | 4.10E-08 |
| rs73124396 | 71579606 | 7 | C | T | 0.205 | 71579606 | 0.002 | 3.10E-10 |
| rs73142879 | 51195932 | 20 | T | C | 0.192 | 51195932 | 0.003 | 3.60E-26 |
| rs73193736 | 108294381 | 12 | G | A | 0.244 | 108294381 | 0.002 | 2.20E-14 |
| rs73213484 | 28489339 | 4 | T | A | 0.141 | 28489339 | 0.003 | 1.70E-15 |
| rs7331420 | 99236471 | 13 | A | G | 0.285 | 99236471 | 0.002 | 6.60E-11 |
| rs7357754 | 92207308 | 9 | G | A | 0.500 | 92207308 | 0.002 | 1.10E-12 |
| rs73601548 | 18549889 | 10 | T | C | 0.115 | 18549889 | 0.003 | 1.30E-08 |
| rs73985439 | 212299249 | 2 | C | A | 0.307 | 212299249 | 0.002 | 1.70E-10 |
| rs7442885 | 87682877 | 5 | G | C | 0.214 | 87682877 | 0.002 | 3.20E-21 |
| rs745249 | 105460333 | 2 | T | C | 0.282 | 105460333 | 0.002 | 1.10E-15 |
| rs74750282 | 114744463 | 7 | C | T | 0.087 | 114744463 | 0.004 | 2.50E-08 |
| rs7498665 | 28883241 | 16 | G | A | 0.400 | 28883241 | 0.002 | 2.30E-40 |
| rs7516554 | 210301331 | 1 | T | C | 0.400 | 210301331 | 0.002 | 2.60E-09 |
| rs7519259 | 66434743 | 1 | A | G | 0.528 | 66434743 | 0.002 | 1.70E-12 |
| rs754635 | 42305131 | 3 | G | C | 0.887 | 42305131 | 0.003 | 1.50E-12 |
| rs75499503 | 26145217 | 6 | T | C | 0.220 | 26145217 | 0.002 | 8.90E-14 |
| rs7571496 | 6169351 | 2 | G | A | 0.261 | 6169351 | 0.002 | 1.80E-12 |
| rs76183894 | 114371939 | 3 | C | T | 0.081 | 114371939 | 0.004 | 1.70E-09 |
| rs7619139 | 25110415 | 3 | A | T | 0.589 | 25110415 | 0.002 | 2.20E-11 |
| rs76702514 | 195148296 | 1 | G | C | 0.211 | 195148296 | 0.002 | 1.20E-11 |
| rs7683836 | 180167906 | 4 | A | G | 0.557 | 180167906 | 0.002 | 8.20E-10 |
| rs7708584 | 153543466 | 5 | G | A | 0.572 | 153543466 | 0.002 | 1.40E-15 |
| rs7761673 | 70357368 | 6 | A | T | 0.220 | 70357368 | 0.002 | 1.30E-08 |
| rs7762794 | 153380228 | 6 | G | A | 0.285 | 153380228 | 0.002 | 9.10E-12 |
| rs7774 | 4801163 | 17 | A | C | 0.310 | 4801163 | 0.002 | 3.30E-12 |
| rs7776021 | 73742152 | 6 | A | G | 0.288 | 73742152 | 0.002 | 1.40E-08 |
| rs7802342 | 137435925 | 7 | G | T | 0.289 | 137435925 | 0.002 | 1.80E-08 |
| rs7805441 | 78121458 | 7 | T | C | 0.502 | 78121458 | 0.002 | 1.80E-11 |
| rs78086698 | 24024639 | 12 | C | T | 0.040 | 24024639 | 0.005 | 3.60E-10 |
| rs784257 | 53397199 | 18 | C | T | 0.813 | 53397199 | 0.003 | 2.00E-12 |
| rs78605811 | 83631491 | 3 | C | A | 0.054 | 83631491 | 0.004 | 1.80E-13 |
| rs7893571 | 16750129 | 10 | T | G | 0.666 | 16750129 | 0.002 | 2.30E-11 |
| rs7924036 | 65191645 | 10 | T | G | 0.503 | 65191645 | 0.002 | 5.10E-13 |
| rs7925100 | 118941596 | 11 | A | G | 0.396 | 118941596 | 0.002 | 3.30E-13 |
| rs7944782 | 130795698 | 11 | G | T | 0.510 | 130795698 | 0.002 | 2.20E-15 |
| rs7947143 | 64090422 | 11 | A | G | 0.163 | 64090422 | 0.003 | 8.30E-12 |
| rs79780963 | 104952499 | 10 | T | C | 0.077 | 104952499 | 0.004 | 1.50E-10 |
| rs7996639 | 97019090 | 13 | A | G | 0.449 | 97019090 | 0.002 | 3.60E-13 |
| rs80135274 | 5286277 | 17 | T | A | 0.070 | 5286277 | 0.004 | 3.80E-08 |
| rs8015400 | 25930988 | 14 | A | C | 0.677 | 25930988 | 0.002 | 6.70E-24 |
| rs8020365 | 79937216 | 14 | A | T | 0.220 | 79937216 | 0.002 | 1.00E-25 |
| rs8024137 | 35837297 | 15 | T | A | 0.848 | 35837297 | 0.003 | 1.60E-08 |
| rs8025516 | 95271872 | 15 | G | T | 0.646 | 95271872 | 0.002 | 1.70E-12 |
| rs8076669 | 15888448 | 17 | C | T | 0.562 | 15888448 | 0.002 | 1.80E-12 |
| rs8089514 | 69224478 | 18 | A | T | 0.369 | 69224478 | 0.002 | 3.90E-10 |
| rs8112818 | 18812785 | 19 | G | A | 0.400 | 18812785 | 0.002 | 1.80E-24 |
| rs8132491 | 40288577 | 21 | A | G | 0.313 | 40288577 | 0.002 | 2.40E-12 |
| rs815163 | 190294726 | 1 | C | T | 0.563 | 190294726 | 0.002 | 1.00E-16 |
| rs852042 | 17091233 | 20 | G | A | 0.759 | 17091233 | 0.002 | 1.40E-08 |
| rs862320 | 69651866 | 16 | T | C | 0.410 | 69651866 | 0.002 | 1.20E-30 |
| rs879620 | 4015729 | 16 | T | C | 0.613 | 4015729 | 0.002 | 2.30E-32 |
| rs909892 | 41982698 | 20 | A | G | 0.135 | 41982698 | 0.003 | 2.60E-10 |
| rs923994 | 67802992 | 4 | G | A | 0.783 | 67802992 | 0.002 | 1.30E-09 |
| rs9267671 | 31880480 | 6 | A | G | 0.061 | 31880480 | 0.004 | 2.60E-10 |
| rs9291822 | 64076515 | 5 | T | C | 0.515 | 64076515 | 0.002 | 8.60E-13 |
| rs9294260 | 83433228 | 6 | A | G | 0.477 | 83433228 | 0.002 | 1.00E-13 |
| rs9349235 | 42516718 | 6 | T | C | 0.411 | 42516718 | 0.002 | 2.60E-08 |
| rs935166 | 26949366 | 2 | A | G | 0.507 | 26949366 | 0.002 | 3.20E-16 |
| rs9463175 | 9510030 | 6 | T | C | 0.339 | 9510030 | 0.002 | 4.40E-08 |
| rs9478496 | 154333183 | 6 | C | T | 0.164 | 154333183 | 0.003 | 1.10E-11 |
| rs9515446 | 112217108 | 13 | G | A | 0.448 | 112217108 | 0.002 | 3.20E-14 |
| rs9522180 | 111970212 | 13 | T | C | 0.553 | 111970212 | 0.002 | 1.50E-12 |
| rs9571687 | 67472713 | 13 | A | C | 0.329 | 67472713 | 0.002 | 1.40E-10 |
| rs9638713 | 14645949 | 7 | G | A | 0.975 | 14645949 | 0.006 | 1.20E-08 |
| rs9673839 | 76895693 | 16 | G | A | 0.491 | 76895693 | 0.002 | 5.50E-11 |
| rs9830592 | 104631603 | 3 | A | C | 0.582 | 104631603 | 0.002 | 1.00E-14 |
| rs9839081 | 123051230 | 3 | A | G | 0.325 | 123051230 | 0.002 | 4.60E-08 |
| rs9843653 | 49920571 | 3 | C | T | 0.512 | 49920571 | 0.002 | 2.90E-50 |
| rs9876664 | 85806313 | 3 | T | G | 0.375 | 85806313 | 0.002 | 9.60E-19 |
| rs9888533 | 107854612 | 13 | T | C | 0.538 | 107854612 | 0.002 | 2.70E-09 |
| rs9926784 | 19941968 | 16 | C | T | 0.185 | 19941968 | 0.003 | 8.60E-21 |
| rs9951619 | 56882326 | 18 | G | T | 0.767 | 56882326 | 0.002 | 9.50E-10 |

EA, effect allele; OA, other allele; SNP, single nucleotide polymorphism.
