# Supplementary material for: Causal association between adiposity and hemorrhoids: a Mendelian randomization study
Source: Front Med (Lausanne). 2023 Oct 6;10:1229925. doi: 10.3389/fmed.2023.1229925 (PMC10587414; doi:10.3389/fmed.2023.1229925)
Supplement: Supplementary file 3 [file Table_3.docx]

Supplementary Table 3 Instrumental genetic variants for body fat percentage.

| rsID of SNP | Position | Chr | Allele | | Effect allele frequency | Association with body fat percentage | | |
| --- | --- | --- | --- | --- | --- | --- | --- | --- |
|  |  |  | EA | OA |  | Effect size (beta) | Standard error of beta | *P*-value |
| rs10050620 | 63927239 | 5 | T | C | 0.325 | -0.010 | 0.002 | 3.80E-10 |
| rs10100245 | 77226919 | 8 | A | G | 0.564 | 0.015 | 0.002 | 1.40E-22 |
| rs10116857 | 16595649 | 9 | A | C | 0.062 | -0.022 | 0.003 | 7.90E-12 |
| rs1013293 | 62570321 | 1 | A | G | 0.430 | -0.014 | 0.002 | 4.10E-19 |
| rs10146997 | 79945162 | 14 | G | A | 0.222 | 0.019 | 0.002 | 6.30E-24 |
| rs10175266 | 36784987 | 2 | G | A | 0.369 | 0.013 | 0.002 | 3.60E-15 |
| rs10187101 | 50742227 | 2 | T | C | 0.364 | -0.010 | 0.002 | 1.40E-10 |
| rs10209821 | 228980358 | 2 | T | C | 0.343 | 0.012 | 0.002 | 2.20E-14 |
| rs10245306 | 158029340 | 7 | C | G | 0.686 | 0.009 | 0.002 | 4.30E-08 |
| rs10259620 | 27202289 | 7 | G | A | 0.787 | -0.014 | 0.002 | 1.60E-13 |
| rs1038088 | 28074563 | 17 | G | T | 0.519 | 0.013 | 0.002 | 4.00E-16 |
| rs10423928 | 46182304 | 19 | A | T | 0.194 | -0.023 | 0.002 | 7.40E-33 |
| rs10496731 | 135597628 | 2 | G | T | 0.375 | -0.012 | 0.002 | 3.70E-13 |
| rs10505836 | 19288508 | 12 | C | A | 0.860 | 0.013 | 0.002 | 9.60E-09 |
| rs10510025 | 118650996 | 10 | T | C | 0.247 | 0.013 | 0.002 | 5.60E-13 |
| rs10513935 | 1660497 | 18 | A | G | 0.302 | -0.009 | 0.002 | 2.20E-08 |
| rs1056441 | 62370349 | 20 | C | T | 0.675 | 0.012 | 0.002 | 2.50E-13 |
| rs10756798 | 16739763 | 9 | T | C | 0.642 | -0.014 | 0.002 | 2.80E-18 |
| rs10799778 | 23313353 | 1 | G | T | 0.834 | -0.013 | 0.002 | 2.50E-10 |
| rs10854853 | 48874412 | 22 | T | G | 0.457 | 0.010 | 0.002 | 1.60E-11 |
| rs1086103 | 145920099 | 4 | A | C | 0.046 | -0.021 | 0.004 | 6.70E-09 |
| rs10867315 | 81574729 | 9 | A | G | 0.191 | -0.011 | 0.002 | 1.50E-08 |
| rs10896012 | 65278461 | 11 | C | T | 0.217 | 0.012 | 0.002 | 7.80E-10 |
| rs10938397 | 45182527 | 4 | G | A | 0.434 | 0.020 | 0.002 | 2.00E-38 |
| rs10959841 | 11469190 | 9 | C | T | 0.387 | -0.009 | 0.002 | 9.20E-09 |
| rs10999460 | 72428283 | 10 | T | C | 0.266 | 0.017 | 0.002 | 6.10E-22 |
| rs11012732 | 21830104 | 10 | G | A | 0.332 | 0.017 | 0.002 | 3.80E-24 |
| rs11022718 | 13268144 | 11 | T | C | 0.203 | -0.011 | 0.002 | 2.50E-08 |
| rs11030016 | 27487992 | 11 | T | C | 0.740 | 0.013 | 0.002 | 5.20E-14 |
| rs11030108 | 27695464 | 11 | G | A | 0.680 | -0.019 | 0.002 | 3.80E-29 |
| rs11062595 | 3367760 | 12 | G | C | 0.092 | -0.015 | 0.003 | 9.50E-09 |
| rs11079849 | 47090785 | 17 | T | C | 0.329 | -0.013 | 0.002 | 2.30E-15 |
| rs11105842 | 91243910 | 12 | A | G | 0.368 | -0.010 | 0.002 | 2.50E-10 |
| rs11122450 | 230301811 | 1 | G | T | 0.612 | -0.010 | 0.002 | 1.30E-10 |
| rs11129660 | 35695961 | 3 | T | C | 0.210 | 0.011 | 0.002 | 5.80E-09 |
| rs11150745 | 78757626 | 17 | G | A | 0.318 | -0.013 | 0.002 | 2.20E-14 |
| rs11165643 | 96924097 | 1 | T | C | 0.590 | 0.015 | 0.002 | 5.90E-21 |
| rs11205303 | 149906413 | 1 | C | T | 0.407 | 0.018 | 0.002 | 1.30E-31 |
| rs11222371 | 130745719 | 11 | T | C | 0.409 | 0.012 | 0.002 | 5.00E-14 |
| rs11245344 | 126418782 | 10 | T | C | 0.571 | 0.009 | 0.002 | 1.90E-09 |
| rs112710809 | 73550401 | 4 | T | C | 0.064 | 0.021 | 0.003 | 4.50E-11 |
| rs112852122 | 47498117 | 20 | A | G | 0.158 | -0.016 | 0.002 | 2.10E-14 |
| rs11343 | 19279464 | 16 | G | T | 0.561 | 0.009 | 0.002 | 4.40E-08 |
| rs113503736 | 19204890 | 4 | G | T | 0.223 | -0.010 | 0.002 | 2.90E-08 |
| rs113941571 | 52268996 | 15 | T | C | 0.079 | 0.021 | 0.003 | 1.70E-12 |
| rs114295766 | 99356361 | 3 | T | A | 0.070 | -0.020 | 0.003 | 1.10E-09 |
| rs11538 | 18220831 | 22 | G | A | 0.172 | 0.012 | 0.002 | 2.80E-09 |
| rs11619393 | 20262266 | 13 | C | T | 0.145 | 0.015 | 0.002 | 2.50E-11 |
| rs11619722 | 27998600 | 13 | C | T | 0.301 | -0.011 | 0.002 | 1.30E-10 |
| rs11664106 | 2846812 | 18 | T | A | 0.374 | 0.011 | 0.002 | 1.80E-11 |
| rs11664848 | 42575661 | 18 | G | C | 0.660 | 0.010 | 0.002 | 3.60E-09 |
| rs11666808 | 18383506 | 19 | C | T | 0.625 | -0.018 | 0.002 | 1.90E-28 |
| rs117176448 | 27261138 | 8 | G | C | 0.096 | 0.017 | 0.003 | 3.60E-11 |
| rs11782074 | 142617096 | 8 | T | G | 0.384 | 0.011 | 0.002 | 3.60E-11 |
| rs11786089 | 21975521 | 8 | G | A | 0.460 | 0.011 | 0.002 | 3.10E-13 |
| rs11852419 | 41456374 | 15 | T | A | 0.262 | 0.011 | 0.002 | 1.30E-09 |
| rs11855853 | 78012618 | 15 | T | C | 0.269 | -0.010 | 0.002 | 2.70E-09 |
| rs11866219 | 69549749 | 16 | C | A | 0.584 | -0.016 | 0.002 | 1.30E-23 |
| rs12042959 | 243533273 | 1 | G | A | 0.144 | -0.015 | 0.002 | 3.70E-11 |
| rs12053559 | 138416391 | 2 | G | T | 0.471 | 0.009 | 0.002 | 2.90E-08 |
| rs12072739 | 98315893 | 1 | G | A | 0.225 | 0.013 | 0.002 | 3.70E-13 |
| rs12103006 | 24726237 | 16 | G | A | 0.569 | 0.010 | 0.002 | 1.80E-11 |
| rs1229984 | 100239319 | 4 | C | T | 0.973 | 0.031 | 0.005 | 3.30E-11 |
| rs12375196 | 103416541 | 7 | A | C | 0.424 | 0.010 | 0.002 | 5.20E-11 |
| rs12376870 | 117890567 | 9 | A | G | 0.238 | -0.011 | 0.002 | 1.30E-09 |
| rs12402939 | 150615094 | 1 | C | A | 0.392 | -0.010 | 0.002 | 1.10E-10 |
| rs12419272 | 117015516 | 11 | G | C | 0.057 | 0.021 | 0.003 | 5.50E-10 |
| rs12432026 | 75282116 | 14 | G | T | 0.540 | 0.010 | 0.002 | 1.20E-10 |
| rs12441543 | 31689543 | 15 | A | G | 0.287 | 0.011 | 0.002 | 2.50E-11 |
| rs12459965 | 18452195 | 19 | T | C | 0.268 | -0.013 | 0.002 | 4.20E-13 |
| rs12462975 | 30272202 | 19 | A | G | 0.330 | 0.012 | 0.002 | 8.20E-14 |
| rs12475388 | 27039792 | 2 | A | G | 0.486 | -0.010 | 0.002 | 1.60E-10 |
| rs12538435 | 71437104 | 7 | G | A | 0.262 | -0.011 | 0.002 | 3.70E-10 |
| rs12619178 | 100838157 | 2 | T | C | 0.401 | -0.014 | 0.002 | 3.00E-20 |
| rs12628603 | 38119213 | 22 | A | G | 0.617 | -0.010 | 0.002 | 1.60E-09 |
| rs12639116 | 62434127 | 3 | T | C | 0.097 | -0.016 | 0.003 | 7.80E-10 |
| rs12658319 | 153551892 | 5 | T | C | 0.299 | 0.011 | 0.002 | 2.00E-10 |
| rs12670456 | 122102781 | 7 | G | A | 0.302 | 0.009 | 0.002 | 2.10E-08 |
| rs12724928 | 113128931 | 1 | C | T | 0.205 | -0.015 | 0.002 | 1.30E-14 |
| rs1284373 | 33355923 | 1 | T | C | 0.199 | -0.011 | 0.002 | 1.60E-08 |
| rs12890931 | 69753369 | 14 | G | T | 0.362 | 0.012 | 0.002 | 7.40E-14 |
| rs12926311 | 406427 | 16 | C | G | 0.354 | -0.009 | 0.002 | 1.40E-08 |
| rs13026103 | 16611534 | 2 | A | G | 0.741 | 0.010 | 0.002 | 4.30E-08 |
| rs13064797 | 173656059 | 3 | A | G | 0.205 | -0.011 | 0.002 | 1.30E-08 |
| rs13107325 | 103188709 | 4 | T | C | 0.075 | 0.032 | 0.003 | 2.10E-28 |
| rs13174863 | 139080745 | 5 | G | A | 0.148 | 0.013 | 0.002 | 3.70E-09 |
| rs1318408 | 11925781 | 1 | G | A | 0.120 | 0.016 | 0.002 | 3.20E-11 |
| rs1322842 | 20488897 | 6 | G | A | 0.609 | -0.011 | 0.002 | 4.60E-13 |
| rs13249935 | 143611456 | 8 | C | T | 0.367 | 0.010 | 0.002 | 3.40E-09 |
| rs13292699 | 15910044 | 9 | C | A | 0.434 | -0.020 | 0.002 | 1.00E-37 |
| rs13389219 | 165528876 | 2 | T | C | 0.392 | 0.017 | 0.002 | 1.70E-27 |
| rs13408397 | 69582028 | 2 | T | C | 0.411 | -0.013 | 0.002 | 5.70E-17 |
| rs13436840 | 157926656 | 5 | T | C | 0.265 | 0.014 | 0.002 | 6.30E-15 |
| rs1350429 | 41819131 | 12 | G | A | 0.480 | 0.010 | 0.002 | 3.00E-10 |
| rs1377184 | 215246432 | 1 | T | A | 0.749 | 0.013 | 0.002 | 8.40E-13 |
| rs1421334 | 30865733 | 8 | C | A | 0.549 | -0.011 | 0.002 | 4.00E-13 |
| rs1436348 | 104612668 | 3 | G | A | 0.583 | 0.011 | 0.002 | 4.70E-12 |
| rs1441264 | 79580919 | 13 | A | G | 0.594 | 0.012 | 0.002 | 7.80E-14 |
| rs1453055 | 181329491 | 2 | A | G | 0.273 | 0.013 | 0.002 | 2.90E-13 |
| rs1456014 | 46899742 | 2 | G | A | 0.206 | -0.015 | 0.002 | 2.10E-15 |
| rs1469084 | 33868013 | 19 | G | A | 0.546 | 0.009 | 0.002 | 1.40E-08 |
| rs149380583 | 80993786 | 15 | C | G | 0.129 | -0.013 | 0.002 | 3.30E-08 |
| rs1503526 | 63020706 | 5 | C | T | 0.480 | 0.011 | 0.002 | 1.60E-13 |
| rs1559900 | 14207321 | 8 | T | C | 0.286 | 0.010 | 0.002 | 7.10E-09 |
| rs1568488 | 153657951 | 3 | C | G | 0.595 | 0.012 | 0.002 | 1.20E-13 |
| rs1624064 | 26378681 | 6 | C | T | 0.420 | -0.011 | 0.002 | 1.00E-11 |
| rs16916303 | 30823761 | 9 | G | A | 0.120 | -0.014 | 0.002 | 2.10E-09 |
| rs16934748 | 33970319 | 10 | C | T | 0.150 | 0.013 | 0.002 | 2.30E-09 |
| rs16996657 | 15816236 | 20 | C | T | 0.128 | 0.014 | 0.002 | 1.40E-09 |
| rs17016133 | 25313167 | 3 | C | T | 0.130 | -0.014 | 0.002 | 2.00E-09 |
| rs17024393 | 110154688 | 1 | C | T | 0.026 | 0.047 | 0.005 | 1.60E-22 |
| rs17055384 | 59285126 | 13 | T | C | 0.183 | -0.012 | 0.002 | 7.30E-10 |
| rs17172722 | 46620312 | 7 | T | C | 0.419 | -0.011 | 0.002 | 5.80E-12 |
| rs17193211 | 38885506 | 21 | T | C | 0.067 | -0.018 | 0.003 | 9.60E-09 |
| rs1724557 | 137094048 | 4 | A | C | 0.587 | -0.011 | 0.002 | 2.10E-12 |
| rs17522122 | 33302882 | 14 | T | G | 0.471 | 0.013 | 0.002 | 4.40E-16 |
| rs17639996 | 61269668 | 3 | A | G | 0.150 | -0.014 | 0.002 | 3.60E-11 |
| rs17681686 | 40368860 | 6 | C | G | 0.305 | 0.014 | 0.002 | 1.80E-16 |
| rs17704028 | 95140031 | 7 | T | C | 0.147 | -0.015 | 0.002 | 1.70E-11 |
| rs17770336 | 28414625 | 9 | T | C | 0.322 | 0.015 | 0.002 | 5.80E-21 |
| rs17820010 | 50384662 | 5 | G | T | 0.296 | 0.012 | 0.002 | 1.70E-13 |
| rs1782508 | 30243795 | 11 | G | C | 0.655 | -0.009 | 0.002 | 1.70E-08 |
| rs1787013 | 13072979 | 18 | C | T | 0.451 | 0.010 | 0.002 | 5.60E-10 |
| rs1799923 | 42306294 | 3 | G | A | 0.887 | 0.014 | 0.002 | 6.30E-09 |
| rs1801282 | 12393125 | 3 | G | C | 0.120 | 0.030 | 0.002 | 4.60E-37 |
| rs1808629 | 73435964 | 8 | A | G | 0.685 | -0.016 | 0.002 | 1.20E-22 |
| rs1813039 | 76295984 | 8 | A | G | 0.710 | 0.010 | 0.002 | 1.40E-09 |
| rs1861410 | 58933591 | 2 | T | C | 0.555 | -0.014 | 0.002 | 2.10E-18 |
| rs1881505 | 1483177 | 11 | C | T | 0.943 | -0.019 | 0.003 | 1.00E-08 |
| rs1906252 | 98550289 | 6 | A | C | 0.484 | -0.016 | 0.002 | 1.40E-25 |
| rs1945160 | 22164216 | 18 | A | G | 0.376 | -0.010 | 0.002 | 1.80E-09 |
| rs1964675 | 136114945 | 3 | T | C | 0.711 | 0.010 | 0.002 | 1.30E-08 |
| rs1991002 | 52848593 | 5 | G | T | 0.495 | -0.010 | 0.002 | 7.20E-11 |
| rs2002023 | 76848524 | 10 | T | C | 0.409 | 0.010 | 0.002 | 6.80E-11 |
| rs2008018 | 61954471 | 17 | A | G | 0.322 | 0.011 | 0.002 | 2.40E-11 |
| rs2034946 | 73863589 | 17 | G | T | 0.209 | 0.012 | 0.002 | 3.30E-10 |
| rs2108635 | 2159556 | 12 | G | A | 0.339 | 0.010 | 0.002 | 1.80E-09 |
| rs2111281 | 108165360 | 12 | C | A | 0.367 | 0.012 | 0.002 | 5.30E-13 |
| rs215669 | 32378979 | 7 | A | G | 0.612 | -0.012 | 0.002 | 2.60E-14 |
| rs2165991 | 53101700 | 15 | G | A | 0.254 | 0.012 | 0.002 | 3.60E-11 |
| rs2172131 | 133978962 | 10 | C | T | 0.579 | -0.011 | 0.002 | 1.30E-12 |
| rs217672 | 62361021 | 14 | C | A | 0.272 | 0.013 | 0.002 | 1.00E-13 |
| rs2178899 | 31606756 | 6 | T | A | 0.129 | -0.019 | 0.002 | 8.50E-17 |
| rs2190788 | 144484261 | 5 | T | G | 0.320 | 0.011 | 0.002 | 2.90E-11 |
| rs2192527 | 18329824 | 4 | G | A | 0.466 | 0.014 | 0.002 | 6.60E-20 |
| rs2192649 | 78126279 | 7 | G | T | 0.501 | 0.009 | 0.002 | 3.80E-08 |
| rs2243928 | 111981707 | 13 | G | C | 0.649 | -0.012 | 0.002 | 1.90E-13 |
| rs2276936 | 89726283 | 4 | C | A | 0.470 | -0.012 | 0.002 | 1.20E-15 |
| rs2291127 | 138122477 | 3 | T | C | 0.156 | -0.014 | 0.002 | 7.70E-11 |
| rs2307111 | 75003678 | 5 | C | T | 0.395 | -0.017 | 0.002 | 1.50E-27 |
| rs2371767 | 64718258 | 3 | C | G | 0.278 | 0.012 | 0.002 | 6.20E-12 |
| rs240999 | 111578490 | 6 | T | G | 0.840 | -0.012 | 0.002 | 1.20E-08 |
| rs2415142 | 73431600 | 15 | G | T | 0.549 | 0.013 | 0.002 | 7.70E-18 |
| rs2481899 | 56460686 | 14 | G | A | 0.551 | 0.009 | 0.002 | 2.50E-09 |
| rs2499468 | 51809081 | 6 | A | C | 0.651 | 0.010 | 0.002 | 5.70E-10 |
| rs2508782 | 94269813 | 11 | G | A | 0.570 | 0.009 | 0.002 | 1.70E-09 |
| rs252749 | 77389973 | 5 | A | G | 0.246 | -0.010 | 0.002 | 9.40E-09 |
| rs256904 | 55810305 | 5 | T | A | 0.746 | -0.011 | 0.002 | 2.50E-10 |
| rs262953 | 183467986 | 3 | A | G | 0.626 | -0.010 | 0.002 | 1.20E-10 |
| rs2640465 | 29569645 | 1 | G | A | 0.902 | 0.016 | 0.003 | 9.40E-10 |
| rs2660241 | 4940023 | 16 | C | T | 0.365 | 0.011 | 0.002 | 1.20E-11 |
| rs2678204 | 201800511 | 1 | G | T | 0.340 | 0.014 | 0.002 | 4.70E-17 |
| rs2692741 | 76185292 | 2 | C | G | 0.367 | 0.009 | 0.002 | 1.60E-08 |
| rs2702123 | 24562699 | 2 | C | T | 0.086 | -0.015 | 0.003 | 2.60E-08 |
| rs2785988 | 219744138 | 1 | A | C | 0.297 | 0.021 | 0.002 | 4.90E-36 |
| rs2802774 | 203527812 | 1 | A | C | 0.547 | 0.011 | 0.002 | 1.50E-11 |
| rs2814993 | 34618893 | 6 | A | G | 0.140 | 0.026 | 0.002 | 1.50E-31 |
| rs2855818 | 42290015 | 17 | A | G | 0.242 | 0.015 | 0.002 | 3.70E-17 |
| rs28651380 | 34773227 | 4 | G | A | 0.281 | 0.010 | 0.002 | 1.20E-09 |
| rs28672845 | 88270627 | 3 | C | A | 0.840 | 0.012 | 0.002 | 4.10E-08 |
| rs28714450 | 9777555 | 8 | T | C | 0.675 | -0.010 | 0.002 | 8.70E-10 |
| rs28742003 | 68127769 | 15 | T | C | 0.205 | -0.021 | 0.002 | 1.90E-28 |
| rs2888778 | 41608611 | 2 | G | T | 0.652 | 0.010 | 0.002 | 6.30E-10 |
| rs2943653 | 227047771 | 2 | T | C | 0.673 | -0.016 | 0.002 | 2.90E-21 |
| rs2954033 | 126493746 | 8 | G | A | 0.695 | 0.012 | 0.002 | 1.20E-13 |
| rs2957678 | 10407955 | 11 | C | T | 0.498 | 0.012 | 0.002 | 4.40E-14 |
| rs2960420 | 12314512 | 3 | G | C | 0.351 | -0.009 | 0.002 | 1.80E-08 |
| rs2966859 | 85324544 | 16 | G | A | 0.789 | -0.011 | 0.002 | 4.20E-09 |
| rs2984618 | 47690438 | 1 | T | G | 0.416 | 0.012 | 0.002 | 5.70E-14 |
| rs3113509 | 52932825 | 4 | T | C | 0.732 | -0.011 | 0.002 | 7.10E-10 |
| rs319775 | 31495076 | 17 | C | T | 0.609 | 0.009 | 0.002 | 2.20E-08 |
| rs33503 | 42427957 | 3 | A | G | 0.806 | -0.013 | 0.002 | 3.90E-12 |
| rs33836 | 34008600 | 19 | T | C | 0.464 | -0.016 | 0.002 | 1.50E-25 |
| rs34338229 | 15370313 | 6 | A | G | 0.332 | 0.009 | 0.002 | 4.60E-08 |
| rs34483452 | 87986314 | 5 | A | C | 0.136 | 0.025 | 0.002 | 2.50E-27 |
| rs34580448 | 82810884 | 5 | C | T | 0.041 | -0.031 | 0.004 | 2.10E-15 |
| rs34656389 | 96153214 | 4 | G | A | 0.367 | 0.010 | 0.002 | 1.10E-10 |
| rs35154152 | 155172725 | 1 | C | T | 0.107 | -0.018 | 0.002 | 1.00E-12 |
| rs3743861 | 89818340 | 16 | C | G | 0.415 | -0.010 | 0.002 | 3.70E-11 |
| rs3754963 | 166185707 | 2 | T | A | 0.255 | -0.011 | 0.002 | 7.60E-11 |
| rs3764002 | 108618630 | 12 | T | C | 0.262 | -0.019 | 0.002 | 2.00E-26 |
| rs3765971 | 8445360 | 1 | T | C | 0.658 | -0.010 | 0.002 | 3.00E-10 |
| rs3766823 | 32197257 | 1 | A | G | 0.172 | 0.014 | 0.002 | 6.30E-12 |
| rs3791709 | 212286934 | 2 | T | A | 0.282 | 0.010 | 0.002 | 1.60E-09 |
| rs3803286 | 103246470 | 14 | G | A | 0.667 | -0.012 | 0.002 | 1.40E-12 |
| rs3817428 | 89415247 | 15 | G | C | 0.265 | -0.015 | 0.002 | 2.50E-18 |
| rs3826408 | 7101292 | 17 | T | C | 0.457 | 0.009 | 0.002 | 3.40E-09 |
| rs3911063 | 85906928 | 3 | C | T | 0.322 | -0.012 | 0.002 | 1.60E-13 |
| rs3923501 | 103065884 | 9 | T | C | 0.476 | 0.010 | 0.002 | 3.40E-11 |
| rs394608 | 46581798 | 21 | C | T | 0.538 | 0.012 | 0.002 | 1.60E-15 |
| rs396354 | 86850022 | 2 | C | T | 0.716 | -0.010 | 0.002 | 8.00E-09 |
| rs40071 | 107496102 | 5 | C | T | 0.179 | -0.015 | 0.002 | 1.40E-13 |
| rs41307479 | 116082647 | 9 | G | C | 0.221 | 0.012 | 0.002 | 2.60E-11 |
| rs41310284 | 102447647 | 10 | A | C | 0.101 | -0.020 | 0.003 | 5.70E-15 |
| rs429343 | 147903382 | 2 | G | A | 0.577 | -0.011 | 0.002 | 9.60E-13 |
| rs429358 | 45411941 | 19 | C | T | 0.154 | -0.022 | 0.002 | 9.20E-24 |
| rs4320040 | 157884946 | 3 | G | T | 0.560 | -0.010 | 0.002 | 4.70E-11 |
| rs4398538 | 130724740 | 4 | C | T | 0.643 | -0.010 | 0.002 | 5.20E-10 |
| rs441792 | 105404221 | 2 | G | A | 0.487 | 0.012 | 0.002 | 1.00E-14 |
| rs4466418 | 126323787 | 8 | A | G | 0.562 | 0.010 | 0.002 | 1.40E-10 |
| rs4482463 | 205375909 | 2 | A | C | 0.923 | -0.021 | 0.003 | 3.00E-13 |
| rs4500770 | 74658430 | 16 | T | A | 0.363 | -0.010 | 0.002 | 1.20E-09 |
| rs4547574 | 48541003 | 2 | T | A | 0.228 | -0.011 | 0.002 | 7.80E-09 |
| rs4690324 | 960081 | 4 | A | G | 0.823 | -0.013 | 0.002 | 3.90E-10 |
| rs4709745 | 164105984 | 6 | C | T | 0.307 | 0.009 | 0.002 | 2.30E-08 |
| rs4718964 | 70038969 | 7 | T | G | 0.413 | 0.011 | 0.002 | 1.50E-12 |
| rs4722398 | 3125220 | 7 | T | C | 0.136 | 0.014 | 0.002 | 1.60E-10 |
| rs4759318 | 54420098 | 12 | T | C | 0.362 | 0.010 | 0.002 | 8.10E-11 |
| rs4762951 | 20543307 | 12 | G | A | 0.780 | -0.011 | 0.002 | 1.10E-09 |
| rs4776337 | 67359738 | 15 | A | G | 0.468 | 0.010 | 0.002 | 1.10E-10 |
| rs479018 | 66060546 | 11 | A | G | 0.332 | -0.014 | 0.002 | 3.30E-18 |
| rs4790841 | 1835482 | 17 | T | C | 0.155 | -0.021 | 0.002 | 2.50E-22 |
| rs4820323 | 38599767 | 22 | G | C | 0.581 | -0.017 | 0.002 | 3.50E-27 |
| rs482787 | 99767024 | 10 | C | T | 0.326 | 0.009 | 0.002 | 1.90E-08 |
| rs4876611 | 116671848 | 8 | G | A | 0.720 | 0.017 | 0.002 | 4.30E-24 |
| rs4894808 | 171833266 | 3 | C | G | 0.400 | -0.010 | 0.002 | 3.30E-10 |
| rs4908676 | 7737099 | 1 | G | A | 0.458 | 0.010 | 0.002 | 4.40E-10 |
| rs4959613 | 1835403 | 6 | A | C | 0.594 | 0.010 | 0.002 | 3.50E-09 |
| rs529200 | 173114305 | 3 | G | A | 0.528 | 0.011 | 0.002 | 4.20E-12 |
| rs543874 | 177889480 | 1 | G | A | 0.205 | 0.030 | 0.002 | 1.70E-56 |
| rs55707359 | 46159333 | 11 | G | T | 0.015 | 0.038 | 0.006 | 3.10E-09 |
| rs55810445 | 2103739 | 7 | T | C | 0.151 | -0.014 | 0.002 | 7.90E-11 |
| rs55924785 | 136929586 | 9 | T | C | 0.165 | -0.012 | 0.002 | 2.90E-09 |
| rs56094641 | 53806453 | 16 | G | A | 0.405 | 0.041 | 0.002 | 2.50E-150 |
| rs56218501 | 46365636 | 20 | T | C | 0.212 | -0.013 | 0.002 | 8.60E-12 |
| rs56328878 | 159459040 | 2 | A | C | 0.267 | -0.010 | 0.002 | 2.00E-08 |
| rs56399737 | 33381721 | 13 | T | C | 0.449 | -0.012 | 0.002 | 2.60E-14 |
| rs57636386 | 58048295 | 18 | C | T | 0.084 | -0.025 | 0.003 | 1.90E-19 |
| rs57800857 | 140863365 | 4 | C | A | 0.365 | -0.013 | 0.002 | 1.20E-16 |
| rs58120873 | 47313562 | 2 | A | G | 0.087 | -0.016 | 0.003 | 2.80E-09 |
| rs58862095 | 75081418 | 7 | T | C | 0.419 | -0.013 | 0.002 | 5.30E-17 |
| rs59227842 | 43692423 | 11 | G | A | 0.311 | 0.017 | 0.002 | 3.40E-24 |
| rs59499656 | 40768309 | 18 | T | A | 0.343 | -0.014 | 0.002 | 2.80E-17 |
| rs6021948 | 51100420 | 20 | A | T | 0.322 | -0.013 | 0.002 | 2.80E-15 |
| rs6064113 | 53444487 | 20 | C | T | 0.765 | -0.011 | 0.002 | 1.50E-08 |
| rs6103254 | 41990761 | 20 | C | T | 0.127 | -0.015 | 0.002 | 2.20E-10 |
| rs61754230 | 72179446 | 12 | T | C | 0.020 | 0.031 | 0.006 | 1.70E-08 |
| rs61903695 | 89922417 | 11 | G | A | 0.255 | 0.012 | 0.002 | 2.60E-11 |
| rs61910767 | 134515899 | 11 | T | C | 0.164 | -0.014 | 0.002 | 6.80E-12 |
| rs61969510 | 86484025 | 13 | C | T | 0.279 | 0.013 | 0.002 | 2.10E-13 |
| rs61975147 | 59416558 | 14 | C | T | 0.167 | -0.014 | 0.002 | 7.30E-12 |
| rs61986205 | 41271485 | 14 | G | A | 0.082 | 0.019 | 0.003 | 2.30E-11 |
| rs62107261 | 422144 | 2 | C | T | 0.048 | -0.048 | 0.004 | 1.70E-40 |
| rs62190394 | 230624929 | 2 | T | C | 0.317 | 0.015 | 0.002 | 4.80E-20 |
| rs62218301 | 36741262 | 21 | G | A | 0.166 | -0.013 | 0.002 | 8.70E-10 |
| rs62413414 | 80310375 | 6 | T | C | 0.152 | 0.013 | 0.002 | 3.30E-09 |
| rs62443626 | 39056649 | 7 | A | G | 0.464 | -0.011 | 0.002 | 8.30E-12 |
| rs62621197 | 8670147 | 19 | T | C | 0.037 | -0.025 | 0.004 | 3.80E-09 |
| rs6480350 | 70396822 | 10 | C | T | 0.575 | -0.010 | 0.002 | 1.10E-09 |
| rs6491427 | 99113166 | 13 | G | A | 0.289 | -0.014 | 0.002 | 2.00E-16 |
| rs6500594 | 4316498 | 16 | G | T | 0.247 | 0.011 | 0.002 | 6.70E-10 |
| rs6561937 | 58257667 | 13 | A | T | 0.754 | -0.012 | 0.002 | 6.90E-11 |
| rs6567160 | 57829135 | 18 | C | T | 0.233 | 0.025 | 0.002 | 3.70E-44 |
| rs6575340 | 94023972 | 14 | A | G | 0.636 | 0.013 | 0.002 | 7.80E-17 |
| rs6602997 | 84521398 | 15 | T | C | 0.712 | 0.023 | 0.002 | 4.80E-40 |
| rs6688826 | 80812329 | 1 | C | T | 0.298 | 0.011 | 0.002 | 2.80E-10 |
| rs6693294 | 49879122 | 1 | G | A | 0.689 | -0.014 | 0.002 | 1.70E-16 |
| rs6744646 | 628504 | 2 | G | A | 0.828 | 0.028 | 0.002 | 2.30E-43 |
| rs6749911 | 105962339 | 2 | A | G | 0.127 | 0.013 | 0.002 | 3.80E-08 |
| rs6750646 | 193872415 | 2 | T | C | 0.201 | -0.012 | 0.002 | 1.00E-09 |
| rs6752378 | 25150116 | 2 | A | C | 0.486 | 0.022 | 0.002 | 4.80E-48 |
| rs6754292 | 217614584 | 2 | T | C | 0.645 | -0.009 | 0.002 | 8.40E-09 |
| rs67609008 | 126640936 | 10 | C | T | 0.284 | 0.011 | 0.002 | 3.90E-11 |
| rs6840236 | 56289785 | 4 | C | T | 0.465 | 0.012 | 0.002 | 4.30E-15 |
| rs6847975 | 80812960 | 4 | A | G | 0.356 | 0.011 | 0.002 | 2.10E-12 |
| rs685149 | 57657413 | 11 | G | A | 0.645 | -0.010 | 0.002 | 3.40E-09 |
| rs6875585 | 95593462 | 5 | C | A | 0.670 | 0.011 | 0.002 | 9.60E-12 |
| rs6927268 | 108865663 | 6 | G | T | 0.206 | -0.014 | 0.002 | 3.20E-13 |
| rs6948959 | 50697051 | 7 | A | G | 0.744 | -0.011 | 0.002 | 2.60E-09 |
| rs6973656 | 77422583 | 7 | G | A | 0.397 | 0.009 | 0.002 | 3.70E-09 |
| rs6977416 | 150542711 | 7 | A | G | 0.334 | -0.012 | 0.002 | 7.60E-13 |
| rs7020 | 25278600 | 20 | A | G | 0.437 | 0.013 | 0.002 | 4.50E-17 |
| rs7027304 | 129408290 | 9 | T | C | 0.653 | 0.012 | 0.002 | 1.10E-12 |
| rs704061 | 89771903 | 12 | C | T | 0.455 | 0.014 | 0.002 | 9.10E-19 |
| rs7046679 | 124638934 | 9 | G | C | 0.679 | -0.009 | 0.002 | 3.20E-08 |
| rs7124681 | 47529947 | 11 | A | C | 0.408 | 0.022 | 0.002 | 6.60E-46 |
| rs7132908 | 50263148 | 12 | A | G | 0.384 | 0.020 | 0.002 | 1.50E-35 |
| rs7133378 | 124409502 | 12 | A | G | 0.319 | 0.019 | 0.002 | 3.30E-29 |
| rs71658797 | 77967507 | 1 | A | T | 0.121 | 0.024 | 0.002 | 1.70E-23 |
| rs719802 | 113234679 | 11 | C | T | 0.614 | -0.012 | 0.002 | 3.10E-13 |
| rs7206608 | 82872628 | 16 | G | C | 0.322 | 0.010 | 0.002 | 7.60E-10 |
| rs7216121 | 21281663 | 17 | G | A | 0.626 | -0.011 | 0.002 | 1.30E-11 |
| rs7218014 | 65832016 | 17 | C | T | 0.197 | 0.021 | 0.002 | 1.70E-27 |
| rs72634814 | 1538046 | 1 | A | G | 0.345 | -0.013 | 0.002 | 2.50E-14 |
| rs72681698 | 51207741 | 14 | C | T | 0.011 | -0.053 | 0.007 | 2.30E-12 |
| rs72697297 | 93069989 | 14 | C | T | 0.180 | -0.015 | 0.002 | 4.50E-14 |
| rs72703757 | 144505785 | 8 | C | G | 0.199 | 0.012 | 0.002 | 3.10E-09 |
| rs72755233 | 100692953 | 15 | A | G | 0.111 | -0.016 | 0.002 | 2.70E-11 |
| rs72767957 | 99240793 | 15 | G | A | 0.200 | -0.013 | 0.002 | 5.30E-11 |
| rs72803260 | 51806701 | 16 | G | T | 0.085 | -0.016 | 0.003 | 1.80E-08 |
| rs72892910 | 50816887 | 6 | T | G | 0.172 | 0.022 | 0.002 | 2.30E-27 |
| rs72917533 | 175238924 | 2 | C | T | 0.185 | -0.014 | 0.002 | 2.70E-12 |
| rs72976986 | 4050424 | 19 | A | G | 0.190 | -0.017 | 0.002 | 6.70E-18 |
| rs72995085 | 143193971 | 6 | C | T | 0.177 | -0.012 | 0.002 | 6.00E-09 |
| rs7321331 | 31015138 | 13 | A | G | 0.742 | 0.011 | 0.002 | 1.40E-09 |
| rs73213501 | 28514830 | 4 | C | A | 0.172 | -0.015 | 0.002 | 8.20E-14 |
| rs7357754 | 92207308 | 9 | G | A | 0.500 | 0.012 | 0.002 | 5.90E-14 |
| rs74288880 | 59275800 | 5 | T | C | 0.115 | -0.014 | 0.002 | 2.00E-08 |
| rs7442885 | 87682877 | 5 | G | C | 0.214 | -0.013 | 0.002 | 9.50E-12 |
| rs74576293 | 3351169 | 12 | C | T | 0.090 | -0.020 | 0.003 | 2.70E-14 |
| rs74618095 | 40683196 | 21 | C | T | 0.158 | 0.013 | 0.002 | 4.90E-10 |
| rs7463186 | 87517201 | 8 | G | A | 0.516 | 0.009 | 0.002 | 1.10E-08 |
| rs7498665 | 28883241 | 16 | G | A | 0.400 | 0.023 | 0.002 | 4.50E-49 |
| rs75135487 | 185805294 | 3 | G | C | 0.137 | -0.014 | 0.002 | 1.10E-10 |
| rs7535438 | 42635359 | 1 | A | C | 0.291 | -0.010 | 0.002 | 2.10E-08 |
| rs75412871 | 121709430 | 12 | T | C | 0.052 | -0.022 | 0.003 | 5.00E-10 |
| rs7575523 | 59335104 | 2 | G | T | 0.603 | -0.015 | 0.002 | 1.70E-22 |
| rs7598246 | 172866359 | 2 | C | T | 0.584 | 0.009 | 0.002 | 4.40E-08 |
| rs76115890 | 91993627 | 13 | C | T | 0.118 | -0.014 | 0.002 | 8.60E-09 |
| rs7630228 | 71681487 | 3 | C | T | 0.434 | -0.011 | 0.002 | 3.70E-12 |
| rs76345589 | 84185140 | 3 | G | C | 0.067 | -0.020 | 0.003 | 1.10E-10 |
| rs7680610 | 24118165 | 4 | G | A | 0.652 | 0.009 | 0.002 | 3.70E-08 |
| rs76856798 | 243922215 | 1 | T | C | 0.018 | 0.032 | 0.006 | 1.90E-08 |
| rs7762794 | 153380228 | 6 | G | A | 0.286 | 0.009 | 0.002 | 4.80E-08 |
| rs7773916 | 70339118 | 6 | T | C | 0.213 | -0.011 | 0.002 | 6.20E-09 |
| rs7789056 | 112908269 | 7 | A | G | 0.563 | -0.013 | 0.002 | 2.90E-16 |
| rs7796825 | 14317417 | 7 | A | G | 0.826 | 0.011 | 0.002 | 2.80E-08 |
| rs78296744 | 122479308 | 12 | A | G | 0.272 | -0.013 | 0.002 | 1.80E-14 |
| rs7843109 | 112360702 | 8 | T | C | 0.689 | 0.010 | 0.002 | 5.20E-10 |
| rs78744936 | 7461343 | 17 | A | G | 0.266 | 0.011 | 0.002 | 1.10E-10 |
| rs7893571 | 16750129 | 10 | T | G | 0.666 | 0.010 | 0.002 | 9.20E-10 |
| rs7925725 | 131449365 | 11 | C | A | 0.411 | 0.011 | 0.002 | 1.10E-12 |
| rs7942368 | 76465362 | 11 | T | C | 0.218 | -0.011 | 0.002 | 2.60E-08 |
| rs79518326 | 174946883 | 1 | A | C | 0.029 | -0.032 | 0.005 | 5.00E-12 |
| rs7960609 | 18161569 | 12 | G | A | 0.327 | 0.009 | 0.002 | 2.50E-08 |
| rs7966251 | 103731395 | 12 | A | G | 0.255 | -0.010 | 0.002 | 3.00E-08 |
| rs7972728 | 123076778 | 12 | A | C | 0.738 | 0.017 | 0.002 | 1.20E-21 |
| rs7975187 | 60964108 | 12 | G | A | 0.214 | 0.012 | 0.002 | 8.80E-10 |
| rs798549 | 2760750 | 7 | A | C | 0.731 | -0.010 | 0.002 | 1.30E-08 |
| rs7987928 | 54372377 | 13 | A | G | 0.800 | -0.014 | 0.002 | 8.50E-13 |
| rs8096564 | 39910592 | 18 | T | G | 0.298 | 0.011 | 0.002 | 2.40E-10 |
| rs811054 | 72251132 | 16 | T | C | 0.537 | 0.010 | 0.002 | 8.70E-10 |
| rs812949 | 170506141 | 5 | C | T | 0.729 | 0.013 | 0.002 | 1.30E-14 |
| rs815163 | 190294726 | 1 | C | T | 0.563 | -0.009 | 0.002 | 4.40E-09 |
| rs843901 | 32904636 | 20 | G | T | 0.936 | 0.019 | 0.003 | 7.50E-10 |
| rs853961 | 127003464 | 6 | T | G | 0.506 | 0.012 | 0.002 | 2.30E-15 |
| rs879620 | 4015729 | 16 | T | C | 0.613 | 0.015 | 0.002 | 1.90E-20 |
| rs881929 | 31079371 | 16 | T | G | 0.375 | -0.015 | 0.002 | 2.10E-20 |
| rs885114 | 8678756 | 11 | A | G | 0.276 | -0.015 | 0.002 | 2.50E-17 |
| rs9289630 | 141178670 | 3 | C | G | 0.389 | 0.012 | 0.002 | 5.60E-15 |
| rs9304665 | 47602577 | 19 | A | T | 0.764 | 0.012 | 0.002 | 6.90E-11 |
| rs9321191 | 130165691 | 6 | C | T | 0.199 | -0.011 | 0.002 | 1.70E-08 |
| rs9358912 | 26211146 | 6 | T | G | 0.273 | -0.023 | 0.002 | 2.70E-41 |
| rs9372414 | 97764299 | 6 | T | C | 0.348 | -0.009 | 0.002 | 8.70E-09 |
| rs9389857 | 141372966 | 6 | T | C | 0.052 | 0.020 | 0.003 | 1.10E-08 |
| rs9568867 | 54107352 | 13 | A | G | 0.129 | 0.019 | 0.002 | 7.30E-17 |
| rs957919 | 131629716 | 3 | T | C | 0.278 | 0.014 | 0.002 | 6.40E-16 |
| rs9645335 | 194969572 | 1 | T | C | 0.368 | -0.010 | 0.002 | 2.00E-09 |
| rs972283 | 130466854 | 7 | G | A | 0.512 | -0.014 | 0.002 | 1.50E-20 |
| rs9788550 | 29681138 | 14 | C | G | 0.248 | -0.016 | 0.002 | 1.10E-18 |
| rs9814758 | 123062657 | 3 | G | T | 0.356 | -0.014 | 0.002 | 3.80E-17 |
| rs9843653 | 49920571 | 3 | C | T | 0.512 | 0.017 | 0.002 | 1.30E-27 |
| rs9865173 | 44016658 | 3 | A | T | 0.703 | -0.012 | 0.002 | 1.10E-12 |
| rs9892466 | 3969527 | 17 | A | T | 0.333 | 0.010 | 0.002 | 8.00E-10 |
| rs9955276 | 1839339 | 18 | T | C | 0.144 | 0.013 | 0.002 | 7.40E-09 |
| rs998584 | 43757896 | 6 | A | C | 0.483 | -0.009 | 0.002 | 1.20E-09 |

EA, effect allele; OA, other allele; SNP, single nucleotide polymorphism.
