# Supplementary material for: Causal association between adiposity and hemorrhoids: a Mendelian randomization study
Source: Front Med (Lausanne). 2023 Oct 6;10:1229925. doi: 10.3389/fmed.2023.1229925 (PMC10587414; doi:10.3389/fmed.2023.1229925)
Supplement: Supplementary file 4 [file Table_4.docx]

Supplementary Table 4 Instrumental genetic variants for waist circumference.

| rsID of SNP | Position | Chr | Allele | | Effect allele frequency | Association with waist circumference | | |
| --- | --- | --- | --- | --- | --- | --- | --- | --- |
|  |  |  | EA | OA |  | Effect size (beta) | Standard error of beta | *P*-value |
| rs1013402 | 27712381 | 11 | G | A | 0.318 | 0.025 | 0.002 | 3.90E-39 |
| rs10150482 | 79891882 | 14 | A | G | 0.220 | 0.022 | 0.002 | 4.50E-24 |
| rs10184230 | 171602134 | 2 | T | C | 0.648 | -0.012 | 0.002 | 8.90E-11 |
| rs10185199 | 40282202 | 2 | A | G | 0.281 | -0.011 | 0.002 | 2.00E-08 |
| rs1019240 | 122957315 | 9 | T | A | 0.643 | 0.012 | 0.002 | 3.70E-11 |
| rs10236214 | 150668070 | 7 | T | C | 0.642 | 0.014 | 0.002 | 1.90E-13 |
| rs10248298 | 121963813 | 7 | A | C | 0.366 | 0.013 | 0.002 | 8.80E-13 |
| rs1025065 | 82451159 | 16 | G | T | 0.639 | -0.010 | 0.002 | 3.60E-08 |
| rs10257197 | 76569775 | 7 | G | A | 0.842 | -0.015 | 0.002 | 3.00E-10 |
| rs10269774 | 92253972 | 7 | A | G | 0.326 | 0.012 | 0.002 | 3.60E-10 |
| rs1037702 | 180168024 | 4 | A | G | 0.622 | -0.010 | 0.002 | 3.60E-08 |
| rs10423928 | 46182304 | 19 | A | T | 0.194 | -0.027 | 0.002 | 6.80E-32 |
| rs10471636 | 63979856 | 5 | A | G | 0.509 | -0.010 | 0.002 | 3.00E-08 |
| rs10490869 | 35635145 | 3 | T | A | 0.210 | 0.016 | 0.002 | 1.20E-13 |
| rs10499014 | 97947755 | 6 | G | C | 0.269 | -0.013 | 0.002 | 5.90E-11 |
| rs10505836 | 19288508 | 12 | C | A | 0.860 | 0.015 | 0.003 | 4.10E-09 |
| rs1051613 | 951179 | 4 | A | G | 0.545 | -0.010 | 0.002 | 3.10E-08 |
| rs1056441 | 62370349 | 20 | C | T | 0.675 | 0.013 | 0.002 | 3.10E-11 |
| rs10787738 | 118777371 | 10 | T | C | 0.255 | 0.015 | 0.002 | 2.10E-13 |
| rs10803762 | 161105876 | 2 | A | G | 0.677 | 0.012 | 0.002 | 4.40E-10 |
| rs10824211 | 76363107 | 10 | T | C | 0.140 | 0.015 | 0.003 | 6.30E-09 |
| rs10827380 | 34822258 | 10 | T | C | 0.314 | 0.012 | 0.002 | 1.80E-09 |
| rs10835676 | 30531316 | 11 | G | C | 0.240 | 0.012 | 0.002 | 4.00E-09 |
| rs10938398 | 45186139 | 4 | A | G | 0.434 | 0.022 | 0.002 | 1.00E-34 |
| rs10947793 | 12142817 | 6 | G | A | 0.372 | -0.013 | 0.002 | 8.10E-12 |
| rs10957087 | 60220423 | 8 | A | T | 0.160 | 0.014 | 0.002 | 1.20E-08 |
| rs10992854 | 96437565 | 9 | C | T | 0.682 | -0.011 | 0.002 | 5.40E-09 |
| rs11012732 | 21830104 | 10 | G | A | 0.332 | 0.019 | 0.002 | 2.40E-24 |
| rs11058233 | 122940075 | 12 | G | A | 0.751 | 0.016 | 0.002 | 1.20E-14 |
| rs1108548 | 218634787 | 1 | G | A | 0.277 | 0.012 | 0.002 | 1.20E-09 |
| rs11099020 | 130724902 | 4 | T | C | 0.641 | -0.011 | 0.002 | 1.70E-09 |
| rs1111817 | 23226898 | 9 | G | C | 0.365 | -0.011 | 0.002 | 2.00E-08 |
| rs111258054 | 134611390 | 11 | T | C | 0.184 | 0.016 | 0.002 | 9.50E-12 |
| rs11150745 | 78757626 | 17 | G | A | 0.318 | -0.016 | 0.002 | 6.80E-17 |
| rs11160600 | 101186626 | 14 | G | A | 0.092 | 0.018 | 0.003 | 7.10E-09 |
| rs11162968 | 80784642 | 1 | C | T | 0.316 | 0.012 | 0.002 | 2.10E-10 |
| rs11165493 | 96274928 | 1 | A | G | 0.343 | 0.011 | 0.002 | 1.30E-08 |
| rs1117619 | 173747092 | 3 | G | C | 0.250 | -0.012 | 0.002 | 5.00E-09 |
| rs11196657 | 115976570 | 10 | C | T | 0.237 | 0.012 | 0.002 | 3.40E-09 |
| rs11215381 | 115022233 | 11 | C | T | 0.526 | 0.011 | 0.002 | 4.60E-10 |
| rs11218510 | 121922587 | 11 | A | G | 0.400 | -0.012 | 0.002 | 2.90E-10 |
| rs11223204 | 132652554 | 11 | G | A | 0.434 | 0.012 | 0.002 | 1.50E-11 |
| rs113132247 | 131026108 | 9 | A | G | 0.153 | 0.015 | 0.002 | 8.40E-10 |
| rs113866544 | 46270606 | 17 | C | T | 0.068 | 0.030 | 0.004 | 1.40E-17 |
| rs114964326 | 146015229 | 2 | A | G | 0.029 | -0.030 | 0.005 | 2.10E-08 |
| rs11603984 | 85238368 | 11 | T | G | 0.135 | -0.017 | 0.003 | 8.90E-11 |
| rs11636611 | 36391965 | 15 | T | C | 0.503 | 0.011 | 0.002 | 2.30E-09 |
| rs11639596 | 9370690 | 16 | C | A | 0.251 | -0.012 | 0.002 | 1.10E-08 |
| rs11653367 | 44191483 | 17 | G | A | 0.328 | -0.015 | 0.002 | 2.50E-15 |
| rs11675464 | 204053742 | 2 | G | A | 0.563 | 0.011 | 0.002 | 3.70E-10 |
| rs11704728 | 18130209 | 22 | T | C | 0.196 | 0.013 | 0.002 | 3.90E-09 |
| rs11757278 | 13180454 | 6 | C | T | 0.304 | -0.013 | 0.002 | 4.90E-11 |
| rs11767811 | 71620988 | 7 | A | G | 0.181 | -0.015 | 0.002 | 8.90E-11 |
| rs11773362 | 147668180 | 7 | T | C | 0.336 | -0.010 | 0.002 | 3.20E-08 |
| rs11787216 | 142615222 | 8 | T | C | 0.369 | 0.012 | 0.002 | 1.20E-09 |
| rs1182199 | 2862542 | 7 | A | C | 0.304 | -0.014 | 0.002 | 3.60E-12 |
| rs11824092 | 13346294 | 11 | C | T | 0.636 | 0.012 | 0.002 | 2.40E-11 |
| rs1183668 | 112191837 | 13 | G | C | 0.370 | -0.012 | 0.002 | 9.30E-11 |
| rs11842871 | 31042452 | 13 | T | G | 0.260 | -0.013 | 0.002 | 4.00E-10 |
| rs1188209 | 56462082 | 14 | G | A | 0.552 | 0.010 | 0.002 | 1.60E-08 |
| rs11898037 | 36558986 | 2 | C | T | 0.368 | 0.011 | 0.002 | 1.20E-08 |
| rs1191600 | 30101641 | 14 | A | C | 0.594 | -0.011 | 0.002 | 3.80E-09 |
| rs12001437 | 34074476 | 9 | C | T | 0.368 | 0.011 | 0.002 | 3.80E-09 |
| rs12042959 | 243533273 | 1 | G | A | 0.144 | -0.015 | 0.003 | 5.20E-09 |
| rs12072739 | 98315893 | 1 | G | A | 0.224 | 0.016 | 0.002 | 3.90E-14 |
| rs12103006 | 24726237 | 16 | G | A | 0.569 | 0.012 | 0.002 | 5.10E-12 |
| rs12107172 | 147134039 | 3 | G | A | 0.136 | 0.016 | 0.003 | 1.80E-09 |
| rs12140153 | 62579891 | 1 | T | G | 0.094 | -0.027 | 0.003 | 2.10E-17 |
| rs1218824 | 28010734 | 13 | A | G | 0.661 | 0.012 | 0.002 | 4.20E-11 |
| rs12225345 | 65647105 | 11 | G | A | 0.166 | 0.015 | 0.002 | 1.30E-09 |
| rs12245654 | 87562928 | 10 | C | A | 0.073 | 0.023 | 0.003 | 3.20E-11 |
| rs12273545 | 116911012 | 11 | T | C | 0.056 | 0.022 | 0.004 | 9.40E-09 |
| rs12287076 | 47606865 | 11 | C | G | 0.707 | 0.021 | 0.002 | 1.30E-26 |
| rs1229984 | 100239319 | 4 | C | T | 0.973 | 0.030 | 0.005 | 2.80E-08 |
| rs12375196 | 103416541 | 7 | A | C | 0.424 | 0.013 | 0.002 | 3.80E-13 |
| rs12462975 | 30272202 | 19 | A | G | 0.330 | 0.018 | 0.002 | 5.90E-20 |
| rs12463617 | 629244 | 2 | C | A | 0.828 | 0.043 | 0.002 | 1.80E-74 |
| rs12478299 | 193811641 | 2 | C | T | 0.252 | -0.012 | 0.002 | 1.90E-08 |
| rs12549000 | 118881908 | 8 | A | T | 0.106 | 0.016 | 0.003 | 1.90E-08 |
| rs12877270 | 97047020 | 13 | A | G | 0.442 | 0.012 | 0.002 | 1.10E-10 |
| rs12880641 | 103251452 | 14 | G | T | 0.662 | -0.014 | 0.002 | 2.40E-13 |
| rs12926311 | 406427 | 16 | C | G | 0.354 | -0.012 | 0.002 | 3.20E-11 |
| rs1296328 | 137083193 | 4 | C | A | 0.559 | -0.013 | 0.002 | 3.10E-13 |
| rs12983532 | 18467322 | 19 | T | C | 0.251 | -0.015 | 0.002 | 7.60E-13 |
| rs13033310 | 133523605 | 2 | A | G | 0.253 | 0.013 | 0.002 | 1.30E-09 |
| rs13047416 | 40309436 | 21 | G | C | 0.377 | -0.014 | 0.002 | 6.60E-14 |
| rs13163306 | 136571959 | 5 | A | G | 0.466 | -0.010 | 0.002 | 3.50E-08 |
| rs1320903 | 131758077 | 3 | A | G | 0.320 | 0.017 | 0.002 | 5.20E-19 |
| rs1327259 | 51177811 | 6 | G | A | 0.388 | -0.012 | 0.002 | 3.10E-10 |
| rs13288841 | 28418511 | 9 | A | G | 0.322 | 0.019 | 0.002 | 2.20E-23 |
| rs13322435 | 156795468 | 3 | G | A | 0.404 | -0.017 | 0.002 | 1.90E-20 |
| rs13333747 | 2175373 | 16 | C | T | 0.183 | -0.023 | 0.002 | 1.90E-22 |
| rs1336486 | 40784814 | 13 | G | T | 0.329 | 0.012 | 0.002 | 6.00E-11 |
| rs13410783 | 36789166 | 2 | G | A | 0.370 | 0.014 | 0.002 | 2.40E-14 |
| rs13420048 | 50751414 | 2 | A | C | 0.365 | -0.013 | 0.002 | 7.00E-13 |
| rs13427822 | 213414265 | 2 | G | A | 0.271 | -0.014 | 0.002 | 3.20E-12 |
| rs1346841 | 65651730 | 4 | A | G | 0.405 | -0.011 | 0.002 | 6.40E-09 |
| rs1357079 | 153691949 | 3 | C | T | 0.570 | 0.011 | 0.002 | 6.20E-10 |
| rs1360201 | 73796450 | 9 | T | C | 0.482 | 0.010 | 0.002 | 4.40E-08 |
| rs1411432 | 16728532 | 9 | C | A | 0.186 | 0.015 | 0.002 | 7.70E-11 |
| rs1436348 | 104612668 | 3 | G | A | 0.583 | 0.012 | 0.002 | 5.40E-12 |
| rs1441264 | 79580919 | 13 | A | G | 0.594 | 0.015 | 0.002 | 7.60E-16 |
| rs145350287 | 120907309 | 12 | A | T | 0.040 | -0.032 | 0.005 | 2.00E-12 |
| rs1458156 | 41887940 | 12 | T | C | 0.488 | 0.015 | 0.002 | 3.50E-16 |
| rs1502317 | 8682018 | 11 | T | C | 0.277 | -0.017 | 0.002 | 4.30E-18 |
| rs1559900 | 14207321 | 8 | T | C | 0.286 | 0.013 | 0.002 | 1.10E-10 |
| rs1570298 | 70218298 | 6 | T | A | 0.743 | 0.012 | 0.002 | 3.80E-09 |
| rs1582931 | 122657199 | 5 | A | G | 0.473 | -0.014 | 0.002 | 1.70E-14 |
| rs1609010 | 77227464 | 8 | G | A | 0.566 | 0.015 | 0.002 | 1.30E-16 |
| rs1609303 | 181567556 | 2 | A | T | 0.631 | 0.015 | 0.002 | 2.10E-16 |
| rs1619442 | 81348300 | 9 | C | T | 0.859 | -0.016 | 0.003 | 2.40E-10 |
| rs1625623 | 112301096 | 5 | T | C | 0.372 | 0.011 | 0.002 | 9.80E-09 |
| rs1657930 | 57120989 | 15 | A | G | 0.803 | -0.014 | 0.002 | 2.30E-10 |
| rs1711171 | 136020541 | 3 | C | T | 0.750 | 0.018 | 0.002 | 1.80E-17 |
| rs17296856 | 84557450 | 15 | C | A | 0.281 | -0.016 | 0.002 | 5.00E-15 |
| rs1731246 | 26938216 | 2 | T | G | 0.757 | -0.012 | 0.002 | 1.30E-08 |
| rs17446091 | 27167942 | 8 | C | T | 0.202 | 0.015 | 0.002 | 2.30E-11 |
| rs1752169 | 126586563 | 9 | A | C | 0.251 | 0.014 | 0.002 | 4.50E-12 |
| rs17681738 | 9793240 | 17 | T | C | 0.329 | 0.011 | 0.002 | 3.30E-08 |
| rs1788808 | 21090023 | 18 | G | A | 0.495 | -0.021 | 0.002 | 3.60E-31 |
| rs1799923 | 42306294 | 3 | G | A | 0.887 | 0.017 | 0.003 | 1.20E-09 |
| rs1834144 | 40744790 | 18 | A | C | 0.373 | -0.015 | 0.002 | 3.40E-15 |
| rs1861410 | 58933591 | 2 | T | C | 0.555 | -0.016 | 0.002 | 6.40E-19 |
| rs1902066 | 81346033 | 6 | C | T | 0.562 | 0.011 | 0.002 | 1.40E-09 |
| rs1942826 | 63345799 | 18 | A | G | 0.126 | 0.017 | 0.003 | 4.90E-10 |
| rs2020942 | 28546914 | 17 | T | C | 0.395 | 0.011 | 0.002 | 3.40E-09 |
| rs2074881 | 1970021 | 19 | T | C | 0.168 | -0.015 | 0.002 | 8.00E-10 |
| rs2133561 | 139086651 | 5 | T | A | 0.611 | -0.012 | 0.002 | 3.90E-11 |
| rs215669 | 32378979 | 7 | A | G | 0.612 | -0.013 | 0.002 | 1.10E-11 |
| rs2161097 | 103945178 | 5 | T | C | 0.438 | 0.014 | 0.002 | 2.20E-15 |
| rs2172131 | 133978962 | 10 | C | T | 0.579 | -0.012 | 0.002 | 1.90E-11 |
| rs217672 | 62361021 | 14 | C | A | 0.272 | 0.013 | 0.002 | 2.80E-10 |
| rs2180454 | 29690513 | 14 | C | T | 0.772 | 0.018 | 0.002 | 3.70E-17 |
| rs2183947 | 26159356 | 6 | A | G | 0.225 | -0.022 | 0.002 | 4.90E-25 |
| rs2225909 | 30329744 | 11 | C | T | 0.774 | 0.016 | 0.002 | 1.40E-13 |
| rs2253310 | 108888593 | 6 | G | C | 0.626 | 0.018 | 0.002 | 6.50E-23 |
| rs2302209 | 18324329 | 19 | T | C | 0.289 | 0.020 | 0.002 | 1.10E-23 |
| rs2306593 | 34866546 | 17 | T | C | 0.488 | -0.015 | 0.002 | 1.60E-17 |
| rs2307111 | 75003678 | 5 | C | T | 0.395 | -0.024 | 0.002 | 3.30E-39 |
| rs2376885 | 89826901 | 16 | A | G | 0.324 | -0.011 | 0.002 | 2.80E-08 |
| rs2439823 | 99778226 | 10 | G | A | 0.546 | 0.016 | 0.002 | 6.80E-18 |
| rs245767 | 170525393 | 5 | G | A | 0.730 | 0.015 | 0.002 | 4.50E-13 |
| rs2470549 | 15737698 | 3 | C | T | 0.598 | -0.012 | 0.002 | 5.20E-11 |
| rs2470946 | 104587323 | 7 | T | G | 0.401 | 0.012 | 0.002 | 1.50E-10 |
| rs2482704 | 94182363 | 9 | T | G | 0.427 | -0.012 | 0.002 | 1.50E-10 |
| rs2568958 | 72765116 | 1 | A | G | 0.604 | 0.017 | 0.002 | 1.60E-20 |
| rs2584205 | 2702259 | 18 | A | G | 0.733 | 0.011 | 0.002 | 3.70E-08 |
| rs2618039 | 112324111 | 1 | T | A | 0.381 | 0.012 | 0.002 | 5.20E-11 |
| rs2678204 | 201800511 | 1 | G | T | 0.340 | 0.016 | 0.002 | 7.90E-17 |
| rs2696309 | 36227101 | 10 | C | T | 0.720 | 0.011 | 0.002 | 1.60E-08 |
| rs2725371 | 30854033 | 8 | G | A | 0.696 | -0.015 | 0.002 | 2.60E-15 |
| rs2744938 | 34552110 | 6 | G | A | 0.148 | 0.032 | 0.003 | 5.40E-37 |
| rs28350 | 42418446 | 3 | G | A | 0.821 | -0.014 | 0.002 | 2.50E-09 |
| rs28366156 | 31671498 | 6 | C | T | 0.131 | -0.019 | 0.003 | 1.50E-12 |
| rs28375268 | 4949528 | 16 | T | G | 0.645 | -0.013 | 0.002 | 3.30E-12 |
| rs28489620 | 41804716 | 22 | A | G | 0.290 | -0.012 | 0.002 | 4.40E-10 |
| rs2861692 | 67838719 | 2 | C | T | 0.275 | -0.017 | 0.002 | 7.70E-17 |
| rs2903738 | 32946043 | 19 | T | A | 0.221 | -0.013 | 0.002 | 6.80E-10 |
| rs3087523 | 105977776 | 2 | A | G | 0.125 | 0.017 | 0.003 | 1.00E-09 |
| rs308911 | 86855977 | 2 | G | A | 0.714 | -0.011 | 0.002 | 6.90E-09 |
| rs3113509 | 52932825 | 4 | T | C | 0.732 | -0.012 | 0.002 | 1.00E-09 |
| rs319775 | 31495076 | 17 | C | T | 0.609 | 0.010 | 0.002 | 3.00E-08 |
| rs3212038 | 104178186 | 14 | G | A | 0.329 | 0.013 | 0.002 | 3.50E-11 |
| rs34045288 | 40369081 | 6 | T | C | 0.334 | 0.020 | 0.002 | 5.80E-27 |
| rs34140906 | 58480400 | 13 | C | T | 0.170 | -0.018 | 0.002 | 5.40E-14 |
| rs34234296 | 175166636 | 2 | A | G | 0.392 | -0.013 | 0.002 | 1.30E-12 |
| rs34483452 | 87986314 | 5 | A | C | 0.136 | 0.027 | 0.003 | 7.30E-25 |
| rs34517439 | 78450517 | 1 | A | C | 0.122 | 0.031 | 0.003 | 1.90E-28 |
| rs34882821 | 14783314 | 9 | T | G | 0.339 | 0.011 | 0.002 | 2.10E-08 |
| rs34994596 | 80991447 | 15 | C | T | 0.297 | -0.015 | 0.002 | 1.10E-13 |
| rs35023999 | 113266411 | 11 | C | A | 0.508 | -0.011 | 0.002 | 2.40E-10 |
| rs35216639 | 174572982 | 1 | A | C | 0.111 | 0.017 | 0.003 | 3.30E-09 |
| rs35243581 | 43707364 | 11 | T | C | 0.317 | 0.017 | 0.002 | 1.70E-19 |
| rs35681682 | 69691602 | 2 | C | T | 0.408 | -0.010 | 0.002 | 1.00E-08 |
| rs35882248 | 230627955 | 2 | T | C | 0.317 | 0.016 | 0.002 | 2.80E-16 |
| rs36007635 | 163009335 | 6 | A | G | 0.138 | -0.017 | 0.003 | 1.70E-10 |
| rs36061954 | 38329650 | 8 | T | C | 0.399 | 0.011 | 0.002 | 3.60E-10 |
| rs36140 | 66164160 | 5 | C | A | 0.635 | 0.011 | 0.002 | 2.10E-09 |
| rs36165342 | 122530256 | 12 | C | T | 0.479 | 0.011 | 0.002 | 2.10E-09 |
| rs3764002 | 108618630 | 12 | T | C | 0.262 | -0.016 | 0.002 | 2.80E-15 |
| rs3768321 | 40035928 | 1 | T | G | 0.197 | 0.018 | 0.002 | 3.80E-15 |
| rs3784692 | 67988133 | 15 | T | C | 0.602 | 0.019 | 0.002 | 2.60E-24 |
| rs3806114 | 20482335 | 6 | A | G | 0.668 | -0.011 | 0.002 | 1.80E-08 |
| rs3807566 | 50564204 | 7 | T | G | 0.438 | -0.012 | 0.002 | 1.70E-11 |
| rs3814883 | 29994922 | 16 | T | C | 0.482 | 0.024 | 0.002 | 1.10E-40 |
| rs3826408 | 7101292 | 17 | T | C | 0.457 | 0.011 | 0.002 | 4.10E-10 |
| rs3845344 | 75001480 | 1 | T | C | 0.391 | 0.011 | 0.002 | 4.30E-09 |
| rs3866805 | 6657424 | 1 | A | C | 0.356 | 0.010 | 0.002 | 2.50E-08 |
| rs3935190 | 79084367 | 17 | A | G | 0.537 | -0.013 | 0.002 | 2.80E-12 |
| rs3936510 | 55860866 | 5 | T | G | 0.201 | 0.014 | 0.002 | 7.30E-10 |
| rs400031 | 741999 | 5 | G | A | 0.756 | 0.012 | 0.002 | 2.10E-08 |
| rs40067 | 107439012 | 5 | A | G | 0.170 | -0.016 | 0.002 | 6.10E-11 |
| rs4072917 | 143300279 | 8 | A | G | 0.474 | 0.012 | 0.002 | 7.40E-11 |
| rs4075353 | 102415609 | 10 | A | G | 0.344 | -0.011 | 0.002 | 2.20E-08 |
| rs41279738 | 110082551 | 1 | G | T | 0.026 | 0.052 | 0.006 | 1.70E-20 |
| rs4148155 | 89054667 | 4 | G | A | 0.113 | -0.019 | 0.003 | 1.50E-11 |
| rs4290163 | 104610926 | 10 | T | G | 0.393 | 0.011 | 0.002 | 5.40E-10 |
| rs429343 | 147903382 | 2 | G | A | 0.577 | -0.012 | 0.002 | 5.90E-12 |
| rs429358 | 45411941 | 19 | C | T | 0.154 | -0.027 | 0.002 | 7.50E-28 |
| rs4344019 | 114588540 | 7 | G | A | 0.917 | 0.020 | 0.003 | 5.70E-10 |
| rs4419475 | 96150044 | 4 | T | A | 0.407 | 0.011 | 0.002 | 6.00E-10 |
| rs4456769 | 25190777 | 20 | T | C | 0.333 | 0.013 | 0.002 | 1.80E-12 |
| rs4469245 | 153210953 | 5 | T | A | 0.663 | -0.012 | 0.002 | 1.10E-09 |
| rs4482463 | 205375909 | 2 | A | C | 0.923 | -0.026 | 0.003 | 1.30E-14 |
| rs4525978 | 102133144 | 4 | T | C | 0.735 | -0.011 | 0.002 | 2.10E-08 |
| rs4527444 | 30842780 | 4 | G | A | 0.541 | 0.011 | 0.002 | 4.50E-09 |
| rs4552632 | 164574688 | 5 | A | G | 0.617 | -0.010 | 0.002 | 3.30E-08 |
| rs4689465 | 6490789 | 4 | C | T | 0.525 | -0.011 | 0.002 | 1.30E-09 |
| rs4706004 | 130391918 | 5 | G | A | 0.217 | -0.013 | 0.002 | 5.40E-10 |
| rs4718964 | 70038969 | 7 | T | G | 0.413 | 0.012 | 0.002 | 1.80E-11 |
| rs4722398 | 3125220 | 7 | T | C | 0.136 | 0.017 | 0.003 | 1.00E-10 |
| rs4742782 | 103089143 | 9 | G | C | 0.316 | 0.012 | 0.002 | 8.00E-11 |
| rs4790841 | 1835482 | 17 | T | C | 0.154 | -0.021 | 0.002 | 1.30E-17 |
| rs484455 | 109835686 | 13 | A | G | 0.481 | -0.012 | 0.002 | 1.40E-10 |
| rs4844809 | 209519772 | 1 | C | G | 0.132 | 0.016 | 0.003 | 1.90E-09 |
| rs4851283 | 100894802 | 2 | G | C | 0.685 | -0.017 | 0.002 | 2.50E-19 |
| rs4876611 | 116671848 | 8 | G | A | 0.720 | 0.015 | 0.002 | 5.30E-14 |
| rs4900715 | 47306418 | 14 | A | G | 0.507 | -0.011 | 0.002 | 1.90E-10 |
| rs4908672 | 7725414 | 1 | T | C | 0.393 | 0.011 | 0.002 | 4.70E-10 |
| rs520478 | 124897878 | 6 | T | G | 0.701 | -0.013 | 0.002 | 2.30E-10 |
| rs539515 | 177889025 | 1 | C | A | 0.205 | 0.038 | 0.002 | 1.50E-65 |
| rs55726687 | 991306 | 12 | A | G | 0.210 | 0.020 | 0.002 | 1.10E-19 |
| rs55794894 | 133516821 | 7 | A | G | 0.135 | -0.015 | 0.003 | 2.80E-08 |
| rs557951 | 62713263 | 3 | G | T | 0.313 | 0.012 | 0.002 | 4.00E-10 |
| rs559231 | 39644247 | 18 | T | G | 0.393 | 0.011 | 0.002 | 5.20E-09 |
| rs56094641 | 53806453 | 16 | G | A | 0.405 | 0.058 | 0.002 | 1.00E-200 |
| rs56803094 | 99222509 | 15 | G | A | 0.227 | -0.013 | 0.002 | 2.60E-09 |
| rs57636386 | 58048295 | 18 | C | T | 0.084 | -0.031 | 0.003 | 1.50E-21 |
| rs587271 | 54743111 | 1 | T | C | 0.687 | 0.012 | 0.002 | 4.10E-09 |
| rs58862095 | 75081418 | 7 | T | C | 0.419 | -0.017 | 0.002 | 7.10E-20 |
| rs588660 | 96886604 | 1 | A | G | 0.584 | 0.016 | 0.002 | 1.00E-17 |
| rs59068084 | 113256737 | 4 | T | G | 0.410 | 0.010 | 0.002 | 2.50E-08 |
| rs59104534 | 25666169 | 8 | T | C | 0.299 | 0.011 | 0.002 | 4.70E-08 |
| rs6001877 | 40712637 | 22 | A | G | 0.340 | -0.011 | 0.002 | 2.40E-08 |
| rs6030803 | 41986507 | 20 | C | T | 0.127 | -0.018 | 0.003 | 6.10E-11 |
| rs6069037 | 53503198 | 20 | A | C | 0.731 | -0.011 | 0.002 | 3.60E-08 |
| rs61223906 | 39447726 | 7 | A | G | 0.339 | -0.011 | 0.002 | 8.30E-09 |
| rs61813324 | 156049877 | 1 | T | C | 0.136 | 0.022 | 0.003 | 9.50E-17 |
| rs61903695 | 89922417 | 11 | G | A | 0.255 | 0.013 | 0.002 | 5.90E-11 |
| rs61969511 | 86484042 | 13 | A | G | 0.279 | 0.012 | 0.002 | 3.80E-09 |
| rs61992671 | 101531854 | 14 | G | A | 0.492 | -0.013 | 0.002 | 3.80E-12 |
| rs62072003 | 52936314 | 17 | T | C | 0.143 | 0.014 | 0.003 | 1.80E-08 |
| rs62243489 | 62482927 | 3 | G | T | 0.259 | -0.016 | 0.002 | 3.00E-14 |
| rs62246311 | 9498143 | 3 | A | G | 0.102 | 0.020 | 0.003 | 3.50E-12 |
| rs62261725 | 85898626 | 3 | G | A | 0.326 | -0.015 | 0.002 | 6.70E-15 |
| rs6493498 | 51754451 | 15 | C | T | 0.545 | -0.013 | 0.002 | 4.20E-13 |
| rs649458 | 23712528 | 1 | A | T | 0.860 | -0.018 | 0.003 | 1.50E-12 |
| rs6536575 | 162091639 | 4 | C | T | 0.519 | 0.011 | 0.002 | 1.40E-09 |
| rs6551304 | 88306596 | 3 | G | A | 0.832 | 0.017 | 0.002 | 2.10E-12 |
| rs6567160 | 57829135 | 18 | C | T | 0.233 | 0.045 | 0.002 | 7.19E-101 |
| rs6575340 | 94023972 | 14 | A | G | 0.636 | 0.016 | 0.002 | 4.60E-18 |
| rs6669341 | 47678458 | 1 | G | A | 0.583 | -0.013 | 0.002 | 4.50E-12 |
| rs6682438 | 33784146 | 1 | C | T | 0.673 | 0.012 | 0.002 | 5.40E-11 |
| rs6693294 | 49879122 | 1 | G | A | 0.689 | -0.017 | 0.002 | 1.10E-18 |
| rs6739755 | 59330227 | 2 | G | A | 0.603 | -0.016 | 0.002 | 3.10E-18 |
| rs67609008 | 126640936 | 10 | C | T | 0.284 | 0.011 | 0.002 | 2.50E-08 |
| rs67632512 | 92251840 | 5 | A | C | 0.117 | 0.017 | 0.003 | 3.80E-09 |
| rs6791983 | 25111890 | 3 | A | C | 0.750 | 0.012 | 0.002 | 2.30E-09 |
| rs6846041 | 18477030 | 4 | G | C | 0.321 | 0.012 | 0.002 | 1.30E-10 |
| rs6849518 | 20120274 | 4 | T | C | 0.124 | 0.022 | 0.003 | 7.10E-16 |
| rs6938973 | 98421721 | 6 | C | T | 0.601 | 0.012 | 0.002 | 4.90E-11 |
| rs7034554 | 37081301 | 9 | G | A | 0.374 | -0.011 | 0.002 | 1.10E-09 |
| rs704061 | 89771903 | 12 | C | T | 0.455 | 0.015 | 0.002 | 4.00E-16 |
| rs7070670 | 61842645 | 10 | T | C | 0.328 | -0.012 | 0.002 | 3.50E-10 |
| rs7115013 | 43934592 | 11 | T | C | 0.443 | -0.011 | 0.002 | 3.70E-09 |
| rs7132908 | 50263148 | 12 | A | G | 0.384 | 0.022 | 0.002 | 1.40E-31 |
| rs71495038 | 33971383 | 10 | A | G | 0.077 | 0.022 | 0.003 | 2.60E-11 |
| rs7169847 | 95272920 | 15 | T | G | 0.636 | -0.010 | 0.002 | 3.80E-08 |
| rs7171864 | 73227249 | 15 | A | G | 0.660 | 0.013 | 0.002 | 1.10E-11 |
| rs7206608 | 82872628 | 16 | G | C | 0.322 | 0.012 | 0.002 | 3.80E-10 |
| rs7218014 | 65832016 | 17 | C | T | 0.197 | 0.022 | 0.002 | 2.70E-23 |
| rs7259070 | 47562509 | 19 | C | T | 0.596 | 0.015 | 0.002 | 6.60E-17 |
| rs72617140 | 229019109 | 2 | C | A | 0.214 | 0.015 | 0.002 | 3.00E-12 |
| rs72618637 | 48953979 | 2 | A | T | 0.189 | -0.013 | 0.002 | 2.10E-08 |
| rs72634826 | 1601052 | 1 | A | G | 0.260 | -0.015 | 0.002 | 5.40E-13 |
| rs72892910 | 50816887 | 6 | T | G | 0.172 | 0.030 | 0.002 | 5.20E-37 |
| rs72959041 | 127454893 | 6 | A | G | 0.049 | 0.030 | 0.004 | 5.70E-13 |
| rs72976986 | 4050424 | 19 | A | G | 0.190 | -0.016 | 0.002 | 7.80E-12 |
| rs73052033 | 185828465 | 3 | C | T | 0.185 | -0.021 | 0.002 | 7.90E-20 |
| rs73068448 | 26385763 | 7 | T | C | 0.171 | -0.015 | 0.002 | 2.00E-10 |
| rs73142879 | 51195932 | 20 | T | C | 0.192 | -0.024 | 0.002 | 2.80E-26 |
| rs7324067 | 65484906 | 13 | C | T | 0.761 | 0.012 | 0.002 | 7.70E-09 |
| rs735033 | 97550852 | 12 | G | A | 0.605 | -0.010 | 0.002 | 2.50E-08 |
| rs7372674 | 196149165 | 3 | A | C | 0.357 | 0.012 | 0.002 | 1.70E-10 |
| rs7377083 | 102708997 | 4 | A | C | 0.431 | 0.014 | 0.002 | 3.20E-15 |
| rs73985439 | 212299249 | 2 | C | A | 0.307 | 0.012 | 0.002 | 1.60E-10 |
| rs74395133 | 61163455 | 3 | C | T | 0.134 | -0.016 | 0.003 | 6.60E-10 |
| rs7442885 | 87682877 | 5 | G | C | 0.214 | -0.020 | 0.002 | 6.80E-21 |
| rs7498044 | 92573639 | 15 | A | G | 0.217 | -0.015 | 0.002 | 4.90E-12 |
| rs7498665 | 28883241 | 16 | G | A | 0.400 | 0.027 | 0.002 | 4.90E-48 |
| rs75035127 | 174973726 | 1 | G | A | 0.030 | -0.033 | 0.005 | 1.50E-10 |
| rs7519259 | 66434743 | 1 | A | G | 0.528 | 0.013 | 0.002 | 1.70E-12 |
| rs7537581 | 2725475 | 1 | A | C | 0.532 | 0.011 | 0.002 | 2.60E-09 |
| rs756717 | 72996162 | 16 | A | G | 0.399 | -0.011 | 0.002 | 6.80E-09 |
| rs76040172 | 46488959 | 21 | A | G | 0.054 | -0.029 | 0.004 | 1.50E-13 |
| rs76286777 | 25195577 | 2 | C | T | 0.218 | 0.023 | 0.002 | 1.80E-27 |
| rs7630382 | 85111108 | 3 | T | C | 0.532 | 0.013 | 0.002 | 9.30E-14 |
| rs765876 | 143185891 | 6 | G | A | 0.489 | -0.010 | 0.002 | 4.10E-08 |
| rs76895963 | 4384844 | 12 | G | T | 0.021 | 0.048 | 0.007 | 2.80E-12 |
| rs7708584 | 153543466 | 5 | G | A | 0.572 | -0.012 | 0.002 | 1.40E-11 |
| rs77165542 | 430975 | 2 | T | C | 0.035 | -0.070 | 0.005 | 9.90E-47 |
| rs7752202 | 33389603 | 6 | T | C | 0.145 | 0.018 | 0.003 | 2.70E-12 |
| rs7755574 | 153364643 | 6 | T | G | 0.283 | 0.011 | 0.002 | 3.90E-08 |
| rs784257 | 53397199 | 18 | C | T | 0.813 | 0.016 | 0.002 | 2.30E-12 |
| rs7845090 | 73449940 | 8 | A | G | 0.709 | -0.020 | 0.002 | 6.30E-23 |
| rs7925100 | 118941596 | 11 | A | G | 0.396 | 0.014 | 0.002 | 1.70E-14 |
| rs7933085 | 130796248 | 11 | G | A | 0.508 | 0.011 | 0.002 | 1.40E-09 |
| rs7952436 | 67024534 | 11 | T | C | 0.082 | -0.029 | 0.003 | 5.90E-19 |
| rs7966251 | 103731395 | 12 | A | G | 0.255 | -0.012 | 0.002 | 1.70E-08 |
| rs8013377 | 25934244 | 14 | C | A | 0.270 | -0.017 | 0.002 | 1.90E-16 |
| rs8024137 | 35837297 | 15 | T | A | 0.848 | 0.014 | 0.003 | 4.60E-08 |
| rs80243702 | 53163603 | 15 | A | G | 0.161 | 0.015 | 0.002 | 6.70E-10 |
| rs8078135 | 21268583 | 17 | T | C | 0.490 | -0.010 | 0.002 | 1.60E-08 |
| rs8097672 | 1839601 | 18 | T | A | 0.145 | 0.017 | 0.003 | 5.00E-11 |
| rs8112818 | 18812785 | 19 | G | A | 0.400 | -0.016 | 0.002 | 3.80E-18 |
| rs815163 | 190294726 | 1 | C | T | 0.563 | -0.013 | 0.002 | 2.70E-13 |
| rs8192675 | 170724883 | 3 | C | T | 0.289 | 0.016 | 0.002 | 4.50E-16 |
| rs852042 | 17091233 | 20 | G | A | 0.759 | -0.012 | 0.002 | 3.00E-08 |
| rs852983 | 142712064 | 5 | A | G | 0.460 | -0.010 | 0.002 | 4.20E-08 |
| rs862227 | 73602926 | 16 | G | A | 0.458 | -0.011 | 0.002 | 8.80E-10 |
| rs862320 | 69651866 | 16 | T | C | 0.410 | -0.018 | 0.002 | 4.40E-23 |
| rs876605 | 77801359 | 5 | G | A | 0.740 | -0.011 | 0.002 | 4.20E-08 |
| rs879620 | 4015729 | 16 | T | C | 0.613 | 0.019 | 0.002 | 4.90E-26 |
| rs883403 | 99047978 | 7 | C | T | 0.154 | -0.018 | 0.002 | 9.50E-13 |
| rs894736 | 54418166 | 12 | G | A | 0.363 | 0.016 | 0.002 | 3.60E-17 |
| rs923994 | 67802992 | 4 | G | A | 0.783 | -0.013 | 0.002 | 2.20E-09 |
| rs9289630 | 141178670 | 3 | C | G | 0.389 | 0.014 | 0.002 | 3.50E-15 |
| rs9294260 | 83433228 | 6 | A | G | 0.477 | 0.013 | 0.002 | 2.40E-13 |
| rs9308964 | 35436262 | 2 | T | C | 0.565 | -0.010 | 0.002 | 3.50E-08 |
| rs9316661 | 54354615 | 13 | C | T | 0.801 | -0.016 | 0.002 | 3.80E-12 |
| rs9370243 | 53789830 | 6 | T | G | 0.082 | 0.020 | 0.003 | 1.90E-09 |
| rs9378676 | 2064264 | 6 | C | A | 0.234 | 0.013 | 0.002 | 7.00E-10 |
| rs945211 | 32191798 | 1 | C | G | 0.616 | 0.010 | 0.002 | 4.10E-08 |
| rs9478496 | 154333183 | 6 | C | T | 0.164 | 0.015 | 0.002 | 1.90E-09 |
| rs9568867 | 54107352 | 13 | A | G | 0.129 | 0.023 | 0.003 | 4.20E-17 |
| rs9584870 | 99245866 | 13 | C | T | 0.366 | -0.011 | 0.002 | 1.10E-08 |
| rs9654453 | 299621 | 5 | C | T | 0.129 | 0.017 | 0.003 | 1.70E-10 |
| rs9673839 | 76895693 | 16 | G | A | 0.491 | 0.011 | 0.002 | 1.30E-09 |
| rs9814758 | 123062657 | 3 | G | T | 0.356 | -0.011 | 0.002 | 2.70E-09 |
| rs9843653 | 49920571 | 3 | C | T | 0.512 | 0.020 | 0.002 | 6.70E-28 |
| rs9888533 | 107854612 | 13 | T | C | 0.538 | 0.011 | 0.002 | 3.00E-09 |
| rs9902846 | 4040774 | 17 | T | C | 0.316 | 0.013 | 0.002 | 4.20E-12 |
| rs9916444 | 42313512 | 17 | G | C | 0.342 | 0.011 | 0.002 | 1.30E-09 |
| rs9926784 | 19941968 | 16 | C | T | 0.185 | -0.013 | 0.002 | 8.70E-09 |

EA, effect allele; OA, other allele; SNP, single nucleotide polymorphism.
