# Supplementary material for: Causal association between adiposity and hemorrhoids: a Mendelian randomization study
Source: Front Med (Lausanne). 2023 Oct 6;10:1229925. doi: 10.3389/fmed.2023.1229925 (PMC10587414; doi:10.3389/fmed.2023.1229925)
Supplement: Supplementary file 5 [file Table_5.docx]

Supplementary Table 5 Instrumental genetic variants for waist-to-hip ratio.

| rsID of SNP | Position | Chr | Allele | | Effect allele frequency | Association with waist-to-hip ratio | | |
| --- | --- | --- | --- | --- | --- | --- | --- | --- |
|  |  |  | EA | OA |  | Effect size (beta) | Standard error of beta | *P*-value |
| rs1011731 | 172346548 | 1 | A | G | 0.542 | -0.019 | 0.542 | 1.10E-08 |
| rs10245353 | 25858614 | 7 | A | C | 0.183 | 0.027 | 0.183 | 1.60E-10 |
| rs10783615 | 54349773 | 12 | A | G | 0.867 | -0.035 | 0.867 | 7.00E-13 |
| rs11048470 | 26487283 | 12 | T | G | 0.233 | 0.025 | 0.233 | 6.30E-12 |
| rs1121980 | 53809247 | 16 | A | G | 0.475 | 0.043 | 0.475 | 1.30E-38 |
| rs1128249 | 165528624 | 2 | T | G | 0.442 | -0.021 | 0.442 | 1.60E-09 |
| rs11663816 | 57876227 | 18 | C | T | 0.317 | 0.025 | 0.317 | 2.70E-11 |
| rs12549058 | 72492238 | 8 | G | T | 0.058 | 0.040 | 0.058 | 3.20E-10 |
| rs1294421 | 6743149 | 6 | G | T | 0.600 | 0.025 | 0.600 | 6.90E-14 |
| rs1316952 | 124399550 | 12 | C | T | 0.117 | -0.028 | 0.117 | 7.30E-09 |
| rs1440372 | 67033151 | 15 | C | T | 0.742 | 0.021 | 0.742 | 7.60E-09 |
| rs1563355 | 219653101 | 1 | C | T | 0.686 | 0.031 | 0.686 | 1.70E-12 |
| rs1569135 | 188115398 | 2 | G | A | 0.467 | -0.024 | 0.467 | 1.00E-12 |
| rs16996700 | 50981945 | 20 | C | T | 0.300 | -0.021 | 0.300 | 1.60E-08 |
| rs17109256 | 79939993 | 14 | A | G | 0.275 | 0.023 | 0.275 | 3.00E-08 |
| rs17451107 | 156797609 | 3 | C | T | 0.375 | -0.023 | 0.375 | 3.50E-11 |
| rs2179129 | 29450923 | 22 | G | A | 0.450 | -0.021 | 0.450 | 1.20E-09 |
| rs2287019 | 46202172 | 19 | T | C | 0.150 | -0.026 | 0.150 | 4.30E-09 |
| rs2765539 | 119549418 | 1 | T | C | 0.708 | 0.027 | 0.708 | 1.10E-12 |
| rs2972164 | 12334416 | 3 | C | T | 0.500 | 0.019 | 0.500 | 2.40E-08 |
| rs3786897 | 33893008 | 19 | G | A | 0.408 | 0.022 | 0.408 | 4.00E-11 |
| rs459193 | 55806751 | 5 | G | A | 0.783 | -0.026 | 0.783 | 6.00E-12 |
| rs4640244 | 21284223 | 17 | G | A | 0.375 | 0.021 | 0.375 | 3.10E-08 |
| rs4715213 | 50911091 | 6 | T | C | 0.100 | 0.024 | 0.100 | 4.00E-08 |
| rs4929927 | 8658485 | 11 | G | A | 0.725 | 0.020 | 0.725 | 7.60E-09 |
| rs929641 | 58792377 | 2 | G | A | 0.383 | -0.020 | 0.383 | 4.20E-09 |
| rs9860730 | 64701146 | 3 | G | A | 0.233 | -0.023 | 0.233 | 2.80E-10 |
| rs998584 | 43757896 | 6 | A | C | 0.475 | 0.029 | 0.475 | 5.00E-15 |

EA, effect allele; OA, other allele; SNP, single nucleotide polymorphism.
