# Supplementary material for: Causal association between adiposity and hemorrhoids: a Mendelian randomization study
Source: Front Med (Lausanne). 2023 Oct 6;10:1229925. doi: 10.3389/fmed.2023.1229925 (PMC10587414; doi:10.3389/fmed.2023.1229925)
Supplement: Supplementary file 6 [file Table_6.docx]

Supplementary Table 6 Leave-one-out sensitivity analysis for the effect of BMI on haemorrhoids.

| Instrumental genetic variant | OR | 95% lower confidence interval | 95% upper confidence interval |
| --- | --- | --- | --- |
| All | 1.005 | 1.003 | 1.008 |
| rs10063055 | 1.005 | 1.003 | 1.008 |
| rs10099330 | 1.005 | 1.003 | 1.008 |
| rs10160769 | 1.005 | 1.003 | 1.008 |
| rs10169594 | 1.005 | 1.003 | 1.008 |
| rs10182416 | 1.005 | 1.003 | 1.008 |
| rs10423928 | 1.005 | 1.003 | 1.008 |
| rs10505836 | 1.005 | 1.003 | 1.008 |
| rs10510025 | 1.005 | 1.003 | 1.008 |
| rs1064213 | 1.005 | 1.003 | 1.008 |
| rs10742752 | 1.005 | 1.003 | 1.008 |
| rs10756714 | 1.005 | 1.002 | 1.008 |
| rs10756792 | 1.005 | 1.002 | 1.008 |
| rs10760277 | 1.005 | 1.003 | 1.008 |
| rs10780248 | 1.005 | 1.003 | 1.008 |
| rs1078141 | 1.005 | 1.003 | 1.008 |
| rs10799778 | 1.005 | 1.003 | 1.008 |
| rs10809621 | 1.005 | 1.003 | 1.008 |
| rs10824211 | 1.005 | 1.003 | 1.008 |
| rs10832778 | 1.005 | 1.003 | 1.008 |
| rs10927006 | 1.005 | 1.003 | 1.008 |
| rs10965698 | 1.005 | 1.003 | 1.008 |
| rs10989067 | 1.005 | 1.003 | 1.008 |
| rs11001963 | 1.005 | 1.003 | 1.008 |
| rs11009685 | 1.005 | 1.003 | 1.008 |
| rs11012732 | 1.005 | 1.002 | 1.008 |
| rs11079849 | 1.005 | 1.003 | 1.008 |
| rs11099020 | 1.005 | 1.003 | 1.008 |
| rs11115160 | 1.005 | 1.003 | 1.008 |
| rs11122450 | 1.005 | 1.003 | 1.008 |
| rs11134679 | 1.005 | 1.003 | 1.008 |
| rs11150745 | 1.005 | 1.003 | 1.008 |
| rs111598585 | 1.005 | 1.003 | 1.008 |
| rs11165643 | 1.005 | 1.003 | 1.008 |
| rs111689389 | 1.005 | 1.003 | 1.008 |
| rs11218510 | 1.005 | 1.003 | 1.008 |
| rs1126930 | 1.005 | 1.003 | 1.008 |
| rs113079574 | 1.005 | 1.003 | 1.008 |
| rs113603865 | 1.005 | 1.003 | 1.008 |
| rs113624107 | 1.005 | 1.003 | 1.008 |
| rs11525873 | 1.005 | 1.003 | 1.008 |
| rs11607476 | 1.005 | 1.003 | 1.008 |
| rs11610621 | 1.005 | 1.003 | 1.008 |
| rs11630647 | 1.005 | 1.003 | 1.008 |
| rs116374395 | 1.005 | 1.003 | 1.008 |
| rs11642090 | 1.005 | 1.003 | 1.008 |
| rs11656076 | 1.005 | 1.003 | 1.008 |
| rs1167311 | 1.005 | 1.003 | 1.008 |
| rs11675464 | 1.005 | 1.003 | 1.008 |
| rs11691869 | 1.005 | 1.003 | 1.008 |
| rs11699828 | 1.005 | 1.003 | 1.008 |
| rs11709402 | 1.005 | 1.002 | 1.008 |
| rs117118217 | 1.005 | 1.003 | 1.008 |
| rs117342986 | 1.005 | 1.003 | 1.008 |
| rs11757278 | 1.005 | 1.003 | 1.008 |
| rs11778219 | 1.005 | 1.003 | 1.008 |
| rs118136827 | 1.005 | 1.003 | 1.008 |
| rs11919665 | 1.005 | 1.003 | 1.008 |
| rs12001437 | 1.005 | 1.003 | 1.008 |
| rs12072739 | 1.005 | 1.003 | 1.008 |
| rs12088284 | 1.005 | 1.003 | 1.008 |
| rs12089815 | 1.005 | 1.003 | 1.008 |
| rs12140153 | 1.005 | 1.003 | 1.008 |
| rs12149660 | 1.005 | 1.003 | 1.008 |
| rs12259464 | 1.005 | 1.003 | 1.008 |
| rs12273545 | 1.005 | 1.003 | 1.008 |
| rs1229984 | 1.005 | 1.003 | 1.008 |
| rs12364470 | 1.005 | 1.003 | 1.008 |
| rs12440603 | 1.005 | 1.003 | 1.008 |
| rs12459368 | 1.005 | 1.003 | 1.008 |
| rs12462975 | 1.005 | 1.003 | 1.008 |
| rs12541408 | 1.005 | 1.003 | 1.008 |
| rs1266874 | 1.005 | 1.003 | 1.008 |
| rs12681792 | 1.005 | 1.003 | 1.008 |
| rs12692596 | 1.005 | 1.003 | 1.008 |
| rs12696039 | 1.005 | 1.003 | 1.008 |
| rs1286058 | 1.005 | 1.003 | 1.008 |
| rs12881629 | 1.005 | 1.003 | 1.008 |
| rs12921986 | 1.005 | 1.003 | 1.008 |
| rs12937411 | 1.005 | 1.003 | 1.008 |
| rs1296328 | 1.005 | 1.003 | 1.008 |
| rs12974458 | 1.005 | 1.003 | 1.008 |
| rs13012070 | 1.005 | 1.003 | 1.008 |
| rs13033310 | 1.005 | 1.003 | 1.008 |
| rs13097918 | 1.005 | 1.003 | 1.008 |
| rs13107325 | 1.005 | 1.002 | 1.008 |
| rs13176429 | 1.005 | 1.003 | 1.008 |
| rs1320251 | 1.005 | 1.003 | 1.008 |
| rs13218383 | 1.005 | 1.003 | 1.008 |
| rs1322842 | 1.005 | 1.003 | 1.008 |
| rs13248187 | 1.005 | 1.003 | 1.008 |
| rs1327259 | 1.005 | 1.003 | 1.008 |
| rs13291723 | 1.005 | 1.003 | 1.008 |
| rs1330199 | 1.005 | 1.003 | 1.008 |
| rs13420048 | 1.005 | 1.003 | 1.008 |
| rs13427822 | 1.005 | 1.003 | 1.008 |
| rs1346841 | 1.005 | 1.003 | 1.008 |
| rs1360201 | 1.005 | 1.003 | 1.008 |
| rs13642 | 1.005 | 1.002 | 1.008 |
| rs140159717 | 1.005 | 1.003 | 1.008 |
| rs1438945 | 1.005 | 1.003 | 1.008 |
| rs1441264 | 1.005 | 1.003 | 1.008 |
| rs1451963 | 1.005 | 1.003 | 1.008 |
| rs1458156 | 1.005 | 1.003 | 1.008 |
| rs145981104 | 1.005 | 1.003 | 1.008 |
| rs146569428 | 1.005 | 1.003 | 1.008 |
| rs1471093 | 1.005 | 1.003 | 1.008 |
| rs1471740 | 1.005 | 1.003 | 1.008 |
| rs147568678 | 1.005 | 1.003 | 1.008 |
| rs1477290 | 1.005 | 1.003 | 1.008 |
| rs147730268 | 1.005 | 1.003 | 1.008 |
| rs1503526 | 1.005 | 1.003 | 1.008 |
| rs156201 | 1.005 | 1.003 | 1.008 |
| rs156914 | 1.005 | 1.003 | 1.008 |
| rs1582931 | 1.005 | 1.003 | 1.008 |
| rs1608113 | 1.005 | 1.003 | 1.008 |
| rs1609010 | 1.005 | 1.003 | 1.008 |
| rs16916303 | 1.005 | 1.003 | 1.008 |
| rs17056301 | 1.005 | 1.003 | 1.008 |
| rs17132130 | 1.005 | 1.003 | 1.008 |
| rs17149254 | 1.005 | 1.003 | 1.008 |
| rs17289010 | 1.005 | 1.003 | 1.008 |
| rs17399739 | 1.005 | 1.003 | 1.008 |
| rs17446299 | 1.005 | 1.003 | 1.008 |
| rs17544384 | 1.005 | 1.003 | 1.008 |
| rs17668356 | 1.005 | 1.003 | 1.008 |
| rs17770336 | 1.005 | 1.003 | 1.008 |
| rs1778830 | 1.005 | 1.003 | 1.008 |
| rs1788808 | 1.005 | 1.003 | 1.008 |
| rs1793636 | 1.005 | 1.003 | 1.008 |
| rs1805123 | 1.005 | 1.003 | 1.008 |
| rs1834144 | 1.005 | 1.003 | 1.008 |
| rs1861410 | 1.005 | 1.002 | 1.008 |
| rs1884897 | 1.005 | 1.003 | 1.008 |
| rs1919243 | 1.005 | 1.003 | 1.008 |
| rs1967772 | 1.005 | 1.003 | 1.008 |
| rs2035936 | 1.005 | 1.003 | 1.008 |
| rs2051559 | 1.005 | 1.003 | 1.008 |
| rs2075466 | 1.005 | 1.003 | 1.008 |
| rs2102278 | 1.005 | 1.003 | 1.008 |
| rs2133561 | 1.005 | 1.003 | 1.008 |
| rs213518 | 1.005 | 1.003 | 1.008 |
| rs2153740 | 1.005 | 1.003 | 1.008 |
| rs215634 | 1.005 | 1.003 | 1.008 |
| rs2172131 | 1.005 | 1.003 | 1.008 |
| rs217672 | 1.005 | 1.003 | 1.008 |
| rs2192158 | 1.005 | 1.003 | 1.008 |
| rs2216931 | 1.005 | 1.003 | 1.008 |
| rs2234458 | 1.005 | 1.003 | 1.008 |
| rs2248551 | 1.005 | 1.003 | 1.008 |
| rs2253310 | 1.005 | 1.003 | 1.008 |
| rs2271189 | 1.005 | 1.003 | 1.008 |
| rs2289379 | 1.005 | 1.003 | 1.008 |
| rs2307111 | 1.005 | 1.003 | 1.008 |
| rs2342892 | 1.005 | 1.003 | 1.008 |
| rs2381404 | 1.005 | 1.003 | 1.008 |
| rs2383377 | 1.005 | 1.003 | 1.008 |
| rs2398861 | 1.005 | 1.002 | 1.008 |
| rs2425816 | 1.005 | 1.003 | 1.008 |
| rs2433733 | 1.005 | 1.003 | 1.008 |
| rs2439823 | 1.005 | 1.003 | 1.008 |
| rs2482356 | 1.005 | 1.003 | 1.008 |
| rs2512892 | 1.005 | 1.003 | 1.008 |
| rs252761 | 1.005 | 1.003 | 1.008 |
| rs2568958 | 1.005 | 1.003 | 1.008 |
| rs2569993 | 1.005 | 1.003 | 1.008 |
| rs2606228 | 1.005 | 1.003 | 1.008 |
| rs2616143 | 1.005 | 1.003 | 1.008 |
| rs2618039 | 1.005 | 1.003 | 1.008 |
| rs2678204 | 1.005 | 1.003 | 1.008 |
| rs2725371 | 1.005 | 1.003 | 1.008 |
| rs2791643 | 1.005 | 1.003 | 1.008 |
| rs28350 | 1.005 | 1.003 | 1.008 |
| rs28366156 | 1.005 | 1.003 | 1.008 |
| rs2837996 | 1.005 | 1.003 | 1.008 |
| rs28404639 | 1.005 | 1.003 | 1.008 |
| rs28489620 | 1.005 | 1.003 | 1.008 |
| rs28568418 | 1.005 | 1.003 | 1.008 |
| rs2861685 | 1.005 | 1.003 | 1.008 |
| rs28670671 | 1.005 | 1.003 | 1.008 |
| rs2870111 | 1.005 | 1.003 | 1.008 |
| rs2875762 | 1.005 | 1.003 | 1.008 |
| rs2899644 | 1.005 | 1.003 | 1.008 |
| rs2920503 | 1.005 | 1.003 | 1.008 |
| rs2962334 | 1.005 | 1.003 | 1.008 |
| rs317656 | 1.005 | 1.003 | 1.008 |
| rs3213943 | 1.005 | 1.003 | 1.008 |
| rs32421 | 1.005 | 1.003 | 1.008 |
| rs329118 | 1.005 | 1.003 | 1.008 |
| rs329651 | 1.005 | 1.003 | 1.008 |
| rs34045288 | 1.005 | 1.002 | 1.008 |
| rs34153025 | 1.005 | 1.003 | 1.008 |
| rs34234296 | 1.005 | 1.003 | 1.008 |
| rs34481751 | 1.005 | 1.003 | 1.008 |
| rs34517439 | 1.005 | 1.003 | 1.008 |
| rs34696181 | 1.005 | 1.003 | 1.008 |
| rs34811474 | 1.005 | 1.002 | 1.008 |
| rs349071 | 1.005 | 1.003 | 1.008 |
| rs35154326 | 1.005 | 1.003 | 1.008 |
| rs35364449 | 1.005 | 1.003 | 1.008 |
| rs355777 | 1.005 | 1.003 | 1.008 |
| rs35697587 | 1.005 | 1.003 | 1.008 |
| rs35697691 | 1.005 | 1.003 | 1.008 |
| rs35809007 | 1.005 | 1.003 | 1.008 |
| rs35957544 | 1.005 | 1.003 | 1.008 |
| rs36007635 | 1.005 | 1.003 | 1.008 |
| rs36061954 | 1.005 | 1.003 | 1.008 |
| rs3764625 | 1.005 | 1.003 | 1.008 |
| rs3784710 | 1.005 | 1.003 | 1.008 |
| rs3803286 | 1.005 | 1.003 | 1.008 |
| rs3807566 | 1.005 | 1.003 | 1.008 |
| rs3814883 | 1.005 | 1.003 | 1.008 |
| rs3845344 | 1.005 | 1.003 | 1.008 |
| rs3851998 | 1.005 | 1.003 | 1.008 |
| rs3866805 | 1.005 | 1.003 | 1.008 |
| rs3897102 | 1.005 | 1.003 | 1.008 |
| rs3901286 | 1.005 | 1.003 | 1.008 |
| rs3902951 | 1.005 | 1.003 | 1.008 |
| rs3935190 | 1.005 | 1.003 | 1.008 |
| rs394608 | 1.005 | 1.003 | 1.008 |
| rs40071 | 1.005 | 1.003 | 1.008 |
| rs4017425 | 1.005 | 1.003 | 1.008 |
| rs4055791 | 1.005 | 1.003 | 1.008 |
| rs406388 | 1.005 | 1.003 | 1.008 |
| rs41279738 | 1.005 | 1.003 | 1.008 |
| rs4148155 | 1.005 | 1.003 | 1.008 |
| rs4261944 | 1.005 | 1.003 | 1.008 |
| rs4267103 | 1.005 | 1.003 | 1.008 |
| rs4284600 | 1.005 | 1.003 | 1.008 |
| rs429343 | 1.005 | 1.002 | 1.008 |
| rs429358 | 1.005 | 1.003 | 1.008 |
| rs4307239 | 1.005 | 1.003 | 1.008 |
| rs4419475 | 1.005 | 1.003 | 1.008 |
| rs4444317 | 1.005 | 1.003 | 1.008 |
| rs4456769 | 1.005 | 1.003 | 1.008 |
| rs4477562 | 1.005 | 1.003 | 1.008 |
| rs4482463 | 1.005 | 1.003 | 1.008 |
| rs45486197 | 1.005 | 1.003 | 1.008 |
| rs4605363 | 1.005 | 1.003 | 1.008 |
| rs4648450 | 1.005 | 1.003 | 1.008 |
| rs4658403 | 1.005 | 1.003 | 1.008 |
| rs4672338 | 1.005 | 1.003 | 1.008 |
| rs4722398 | 1.005 | 1.003 | 1.008 |
| rs4764949 | 1.005 | 1.003 | 1.008 |
| rs4790292 | 1.005 | 1.003 | 1.008 |
| rs4820410 | 1.005 | 1.003 | 1.008 |
| rs4832298 | 1.005 | 1.003 | 1.008 |
| rs4858940 | 1.005 | 1.003 | 1.008 |
| rs4876611 | 1.005 | 1.003 | 1.008 |
| rs4929923 | 1.005 | 1.003 | 1.008 |
| rs5011579 | 1.005 | 1.003 | 1.008 |
| rs512121 | 1.005 | 1.003 | 1.008 |
| rs529200 | 1.005 | 1.003 | 1.008 |
| rs539515 | 1.005 | 1.003 | 1.008 |
| rs55707359 | 1.005 | 1.003 | 1.008 |
| rs55714539 | 1.005 | 1.003 | 1.008 |
| rs55726687 | 1.005 | 1.003 | 1.008 |
| rs55769038 | 1.005 | 1.003 | 1.008 |
| rs558887 | 1.005 | 1.003 | 1.008 |
| rs559231 | 1.005 | 1.003 | 1.008 |
| rs56038322 | 1.005 | 1.003 | 1.008 |
| rs56094641 | 1.005 | 1.003 | 1.008 |
| rs56133507 | 1.005 | 1.003 | 1.008 |
| rs56143236 | 1.005 | 1.003 | 1.008 |
| rs56161855 | 1.005 | 1.003 | 1.008 |
| rs56203622 | 1.005 | 1.003 | 1.008 |
| rs56352336 | 1.005 | 1.003 | 1.008 |
| rs56399737 | 1.005 | 1.003 | 1.008 |
| rs56858768 | 1.005 | 1.003 | 1.008 |
| rs56893062 | 1.005 | 1.003 | 1.008 |
| rs56930105 | 1.005 | 1.003 | 1.008 |
| rs57636386 | 1.005 | 1.003 | 1.008 |
| rs57989773 | 1.005 | 1.003 | 1.008 |
| rs58862095 | 1.005 | 1.003 | 1.008 |
| rs59068084 | 1.005 | 1.003 | 1.008 |
| rs59227842 | 1.005 | 1.003 | 1.008 |
| rs594024 | 1.005 | 1.003 | 1.008 |
| rs6023655 | 1.005 | 1.003 | 1.008 |
| rs60764613 | 1.005 | 1.003 | 1.008 |
| rs61740466 | 1.005 | 1.003 | 1.008 |
| rs61813324 | 1.005 | 1.002 | 1.008 |
| rs61828641 | 1.005 | 1.003 | 1.008 |
| rs61871615 | 1.005 | 1.003 | 1.008 |
| rs61903695 | 1.005 | 1.003 | 1.008 |
| rs61992671 | 1.005 | 1.003 | 1.008 |
| rs62007782 | 1.005 | 1.003 | 1.008 |
| rs62020775 | 1.005 | 1.003 | 1.008 |
| rs62072006 | 1.005 | 1.003 | 1.008 |
| rs62107261 | 1.006 | 1.003 | 1.008 |
| rs62176243 | 1.005 | 1.003 | 1.008 |
| rs62190049 | 1.005 | 1.003 | 1.008 |
| rs62241847 | 1.005 | 1.003 | 1.008 |
| rs62246311 | 1.005 | 1.003 | 1.008 |
| rs62379271 | 1.005 | 1.003 | 1.008 |
| rs62407562 | 1.005 | 1.003 | 1.008 |
| rs6265 | 1.005 | 1.003 | 1.008 |
| rs6430068 | 1.005 | 1.003 | 1.008 |
| rs6444950 | 1.005 | 1.003 | 1.008 |
| rs6545714 | 1.005 | 1.003 | 1.008 |
| rs6560906 | 1.005 | 1.003 | 1.008 |
| rs6561937 | 1.005 | 1.003 | 1.008 |
| rs6567160 | 1.005 | 1.003 | 1.008 |
| rs6575340 | 1.005 | 1.003 | 1.008 |
| rs66679256 | 1.005 | 1.003 | 1.008 |
| rs6669341 | 1.005 | 1.003 | 1.008 |
| rs6682438 | 1.005 | 1.003 | 1.008 |
| rs6705567 | 1.005 | 1.003 | 1.008 |
| rs6707827 | 1.005 | 1.003 | 1.008 |
| rs6710091 | 1.005 | 1.003 | 1.008 |
| rs6713781 | 1.005 | 1.003 | 1.008 |
| rs6725931 | 1.005 | 1.003 | 1.008 |
| rs6744646 | 1.005 | 1.002 | 1.008 |
| rs6752979 | 1.005 | 1.003 | 1.008 |
| rs67609008 | 1.005 | 1.003 | 1.008 |
| rs6769617 | 1.005 | 1.003 | 1.008 |
| rs6774894 | 1.005 | 1.003 | 1.008 |
| rs6777784 | 1.005 | 1.003 | 1.008 |
| rs6831088 | 1.005 | 1.003 | 1.008 |
| rs6843852 | 1.005 | 1.003 | 1.008 |
| rs6909685 | 1.005 | 1.003 | 1.008 |
| rs6922607 | 1.005 | 1.003 | 1.008 |
| rs6938973 | 1.005 | 1.003 | 1.008 |
| rs6950388 | 1.005 | 1.003 | 1.008 |
| rs6962980 | 1.005 | 1.003 | 1.008 |
| rs698147 | 1.005 | 1.003 | 1.008 |
| rs7024334 | 1.005 | 1.003 | 1.008 |
| rs7027304 | 1.005 | 1.003 | 1.008 |
| rs7034554 | 1.005 | 1.003 | 1.008 |
| rs7038943 | 1.005 | 1.003 | 1.008 |
| rs704061 | 1.005 | 1.003 | 1.008 |
| rs7070670 | 1.005 | 1.003 | 1.008 |
| rs7081254 | 1.005 | 1.003 | 1.008 |
| rs7124681 | 1.005 | 1.002 | 1.008 |
| rs7132908 | 1.005 | 1.003 | 1.008 |
| rs71495038 | 1.005 | 1.003 | 1.008 |
| rs7201895 | 1.005 | 1.003 | 1.008 |
| rs7206608 | 1.005 | 1.003 | 1.008 |
| rs7218014 | 1.005 | 1.003 | 1.008 |
| rs7232171 | 1.005 | 1.003 | 1.008 |
| rs723672 | 1.005 | 1.003 | 1.008 |
| rs7250833 | 1.005 | 1.003 | 1.008 |
| rs7259070 | 1.005 | 1.003 | 1.008 |
| rs72634826 | 1.005 | 1.003 | 1.008 |
| rs72649373 | 1.005 | 1.003 | 1.008 |
| rs72673947 | 1.005 | 1.003 | 1.008 |
| rs72892910 | 1.005 | 1.003 | 1.008 |
| rs72976986 | 1.005 | 1.003 | 1.008 |
| rs73026725 | 1.005 | 1.003 | 1.008 |
| rs73052033 | 1.005 | 1.003 | 1.008 |
| rs7306534 | 1.005 | 1.003 | 1.008 |
| rs73124396 | 1.005 | 1.003 | 1.008 |
| rs73142879 | 1.005 | 1.003 | 1.008 |
| rs73193736 | 1.005 | 1.002 | 1.008 |
| rs73213484 | 1.005 | 1.003 | 1.008 |
| rs7331420 | 1.005 | 1.003 | 1.008 |
| rs7357754 | 1.005 | 1.003 | 1.008 |
| rs73601548 | 1.005 | 1.003 | 1.008 |
| rs73985439 | 1.005 | 1.003 | 1.008 |
| rs7442885 | 1.005 | 1.003 | 1.008 |
| rs745249 | 1.005 | 1.003 | 1.008 |
| rs74750282 | 1.005 | 1.003 | 1.008 |
| rs7498665 | 1.005 | 1.002 | 1.008 |
| rs7516554 | 1.005 | 1.003 | 1.008 |
| rs7519259 | 1.005 | 1.003 | 1.008 |
| rs754635 | 1.005 | 1.003 | 1.008 |
| rs75499503 | 1.005 | 1.003 | 1.008 |
| rs7571496 | 1.005 | 1.003 | 1.008 |
| rs76183894 | 1.005 | 1.003 | 1.008 |
| rs7619139 | 1.005 | 1.003 | 1.008 |
| rs76702514 | 1.005 | 1.003 | 1.008 |
| rs7683836 | 1.005 | 1.003 | 1.008 |
| rs7708584 | 1.005 | 1.003 | 1.008 |
| rs7761673 | 1.005 | 1.003 | 1.008 |
| rs7762794 | 1.005 | 1.003 | 1.008 |
| rs7774 | 1.005 | 1.003 | 1.008 |
| rs7776021 | 1.005 | 1.003 | 1.008 |
| rs7802342 | 1.005 | 1.003 | 1.008 |
| rs7805441 | 1.005 | 1.003 | 1.008 |
| rs78086698 | 1.005 | 1.003 | 1.008 |
| rs784257 | 1.005 | 1.003 | 1.008 |
| rs78605811 | 1.005 | 1.003 | 1.008 |
| rs7893571 | 1.005 | 1.003 | 1.008 |
| rs7924036 | 1.005 | 1.003 | 1.008 |
| rs7925100 | 1.005 | 1.003 | 1.008 |
| rs7944782 | 1.005 | 1.003 | 1.008 |
| rs7947143 | 1.005 | 1.003 | 1.008 |
| rs79780963 | 1.005 | 1.003 | 1.008 |
| rs7996639 | 1.005 | 1.003 | 1.008 |
| rs80135274 | 1.005 | 1.003 | 1.008 |
| rs8015400 | 1.005 | 1.003 | 1.008 |
| rs8020365 | 1.005 | 1.003 | 1.008 |
| rs8024137 | 1.005 | 1.003 | 1.008 |
| rs8025516 | 1.005 | 1.003 | 1.008 |
| rs8076669 | 1.005 | 1.003 | 1.008 |
| rs8089514 | 1.005 | 1.003 | 1.008 |
| rs8112818 | 1.005 | 1.003 | 1.008 |
| rs8132491 | 1.005 | 1.003 | 1.008 |
| rs815163 | 1.005 | 1.003 | 1.008 |
| rs852042 | 1.005 | 1.003 | 1.008 |
| rs862320 | 1.005 | 1.003 | 1.008 |
| rs879620 | 1.005 | 1.003 | 1.008 |
| rs909892 | 1.005 | 1.003 | 1.008 |
| rs923994 | 1.005 | 1.003 | 1.008 |
| rs9267671 | 1.005 | 1.003 | 1.008 |
| rs9291822 | 1.005 | 1.002 | 1.008 |
| rs9294260 | 1.005 | 1.003 | 1.008 |
| rs9349235 | 1.005 | 1.003 | 1.008 |
| rs935166 | 1.005 | 1.003 | 1.008 |
| rs9463175 | 1.005 | 1.003 | 1.008 |
| rs9478496 | 1.005 | 1.003 | 1.008 |
| rs9515446 | 1.005 | 1.003 | 1.008 |
| rs9522180 | 1.005 | 1.003 | 1.008 |
| rs9571687 | 1.005 | 1.003 | 1.008 |
| rs9638713 | 1.005 | 1.003 | 1.008 |
| rs9673839 | 1.005 | 1.003 | 1.008 |
| rs9830592 | 1.005 | 1.003 | 1.008 |
| rs9839081 | 1.005 | 1.003 | 1.008 |
| rs9843653 | 1.005 | 1.003 | 1.008 |
| rs9876664 | 1.005 | 1.003 | 1.008 |
| rs9888533 | 1.005 | 1.003 | 1.008 |
| rs9926784 | 1.005 | 1.002 | 1.008 |
| rs9951619 | 1.005 | 1.003 | 1.008 |

BMI, body mass index; OR, odds ratio.
