# Supplementary material for: Causal association between adiposity and hemorrhoids: a Mendelian randomization study
Source: Front Med (Lausanne). 2023 Oct 6;10:1229925. doi: 10.3389/fmed.2023.1229925 (PMC10587414; doi:10.3389/fmed.2023.1229925)
Supplement: Supplementary file 7 [file Table_7.docx]

Supplementary Table 7 Leave-one-out sensitivity analysis for the effect of body fat percentage on haemorrhoids.

| Instrumental genetic variant | OR | 95% lower confidence interval | 95% upper confidence interval |
| --- | --- | --- | --- |
| All | 1.005 | 1.001 | 1.008 |
| rs10050620 | 1.005 | 1.001 | 1.009 |
| rs10100245 | 1.005 | 1.001 | 1.008 |
| rs10116857 | 1.005 | 1.001 | 1.008 |
| rs1013293 | 1.005 | 1.001 | 1.008 |
| rs10146997 | 1.005 | 1.001 | 1.008 |
| rs10175266 | 1.005 | 1.001 | 1.009 |
| rs10187101 | 1.005 | 1.001 | 1.008 |
| rs10209821 | 1.005 | 1.001 | 1.009 |
| rs10245306 | 1.005 | 1.001 | 1.008 |
| rs10259620 | 1.005 | 1.001 | 1.008 |
| rs1038088 | 1.005 | 1.001 | 1.008 |
| rs10423928 | 1.005 | 1.001 | 1.008 |
| rs10496731 | 1.005 | 1.001 | 1.008 |
| rs10505836 | 1.005 | 1.001 | 1.008 |
| rs10510025 | 1.005 | 1.001 | 1.008 |
| rs10513935 | 1.005 | 1.001 | 1.008 |
| rs1056441 | 1.005 | 1.001 | 1.008 |
| rs10756798 | 1.005 | 1.001 | 1.008 |
| rs10799778 | 1.005 | 1.001 | 1.008 |
| rs10854853 | 1.005 | 1.001 | 1.008 |
| rs1086103 | 1.005 | 1.001 | 1.008 |
| rs10867315 | 1.005 | 1.001 | 1.008 |
| rs10896012 | 1.005 | 1.001 | 1.008 |
| rs10938397 | 1.005 | 1.001 | 1.008 |
| rs10959841 | 1.005 | 1.001 | 1.008 |
| rs10999460 | 1.005 | 1.001 | 1.009 |
| rs11012732 | 1.004 | 1.001 | 1.008 |
| rs11022718 | 1.005 | 1.001 | 1.008 |
| rs11030016 | 1.005 | 1.001 | 1.008 |
| rs11030108 | 1.005 | 1.001 | 1.008 |
| rs11062595 | 1.005 | 1.001 | 1.008 |
| rs11079849 | 1.005 | 1.001 | 1.008 |
| rs11105842 | 1.005 | 1.001 | 1.008 |
| rs11122450 | 1.005 | 1.001 | 1.008 |
| rs11129660 | 1.005 | 1.001 | 1.008 |
| rs11150745 | 1.005 | 1.001 | 1.008 |
| rs11165643 | 1.005 | 1.001 | 1.008 |
| rs11205303 | 1.005 | 1.002 | 1.009 |
| rs11222371 | 1.005 | 1.001 | 1.008 |
| rs11245344 | 1.005 | 1.001 | 1.008 |
| rs112710809 | 1.005 | 1.001 | 1.008 |
| rs112852122 | 1.005 | 1.001 | 1.008 |
| rs11343 | 1.005 | 1.001 | 1.008 |
| rs113503736 | 1.005 | 1.001 | 1.009 |
| rs113941571 | 1.005 | 1.001 | 1.008 |
| rs114295766 | 1.005 | 1.001 | 1.008 |
| rs11538 | 1.005 | 1.001 | 1.009 |
| rs11619393 | 1.005 | 1.001 | 1.008 |
| rs11619722 | 1.005 | 1.001 | 1.008 |
| rs11664106 | 1.005 | 1.001 | 1.008 |
| rs11664848 | 1.005 | 1.001 | 1.008 |
| rs11666808 | 1.005 | 1.001 | 1.008 |
| rs117176448 | 1.005 | 1.001 | 1.008 |
| rs11782074 | 1.005 | 1.001 | 1.008 |
| rs11786089 | 1.005 | 1.001 | 1.008 |
| rs11852419 | 1.005 | 1.001 | 1.008 |
| rs11855853 | 1.005 | 1.001 | 1.008 |
| rs11866219 | 1.005 | 1.001 | 1.008 |
| rs12042959 | 1.005 | 1.001 | 1.008 |
| rs12053559 | 1.005 | 1.001 | 1.008 |
| rs12072739 | 1.005 | 1.001 | 1.008 |
| rs12103006 | 1.005 | 1.001 | 1.008 |
| rs1229984 | 1.005 | 1.001 | 1.008 |
| rs12375196 | 1.005 | 1.001 | 1.008 |
| rs12376870 | 1.005 | 1.001 | 1.009 |
| rs12402939 | 1.005 | 1.001 | 1.008 |
| rs12419272 | 1.005 | 1.001 | 1.008 |
| rs12432026 | 1.005 | 1.001 | 1.008 |
| rs12441543 | 1.005 | 1.001 | 1.009 |
| rs12459965 | 1.005 | 1.001 | 1.008 |
| rs12462975 | 1.005 | 1.001 | 1.008 |
| rs12475388 | 1.005 | 1.001 | 1.008 |
| rs12538435 | 1.005 | 1.001 | 1.008 |
| rs12619178 | 1.005 | 1.001 | 1.008 |
| rs12628603 | 1.005 | 1.001 | 1.009 |
| rs12639116 | 1.005 | 1.001 | 1.008 |
| rs12658319 | 1.005 | 1.001 | 1.008 |
| rs12670456 | 1.005 | 1.001 | 1.008 |
| rs12724928 | 1.005 | 1.001 | 1.008 |
| rs1284373 | 1.005 | 1.001 | 1.008 |
| rs12890931 | 1.005 | 1.001 | 1.008 |
| rs12926311 | 1.005 | 1.001 | 1.008 |
| rs13026103 | 1.005 | 1.001 | 1.008 |
| rs13064797 | 1.005 | 1.001 | 1.008 |
| rs13107325 | 1.005 | 1.001 | 1.008 |
| rs13174863 | 1.005 | 1.001 | 1.008 |
| rs1318408 | 1.005 | 1.001 | 1.008 |
| rs1322842 | 1.005 | 1.001 | 1.008 |
| rs13249935 | 1.005 | 1.001 | 1.008 |
| rs13292699 | 1.005 | 1.001 | 1.008 |
| rs13389219 | 1.005 | 1.001 | 1.009 |
| rs13408397 | 1.005 | 1.001 | 1.008 |
| rs13436840 | 1.005 | 1.001 | 1.009 |
| rs1350429 | 1.005 | 1.001 | 1.008 |
| rs1377184 | 1.005 | 1.001 | 1.008 |
| rs1421334 | 1.005 | 1.001 | 1.008 |
| rs1436348 | 1.005 | 1.001 | 1.008 |
| rs1441264 | 1.005 | 1.001 | 1.008 |
| rs1453055 | 1.005 | 1.001 | 1.008 |
| rs1456014 | 1.005 | 1.001 | 1.009 |
| rs1469084 | 1.005 | 1.001 | 1.008 |
| rs149380583 | 1.005 | 1.001 | 1.008 |
| rs1503526 | 1.005 | 1.001 | 1.008 |
| rs1559900 | 1.005 | 1.001 | 1.008 |
| rs1568488 | 1.005 | 1.001 | 1.008 |
| rs1624064 | 1.005 | 1.001 | 1.008 |
| rs16916303 | 1.005 | 1.001 | 1.008 |
| rs16934748 | 1.005 | 1.001 | 1.009 |
| rs16996657 | 1.005 | 1.001 | 1.008 |
| rs17016133 | 1.005 | 1.001 | 1.008 |
| rs17024393 | 1.005 | 1.001 | 1.009 |
| rs17055384 | 1.005 | 1.001 | 1.008 |
| rs17172722 | 1.005 | 1.001 | 1.008 |
| rs17193211 | 1.005 | 1.001 | 1.008 |
| rs1724557 | 1.005 | 1.001 | 1.008 |
| rs17522122 | 1.005 | 1.001 | 1.008 |
| rs17639996 | 1.005 | 1.001 | 1.008 |
| rs17681686 | 1.005 | 1.001 | 1.008 |
| rs17704028 | 1.005 | 1.001 | 1.008 |
| rs17770336 | 1.005 | 1.001 | 1.008 |
| rs17820010 | 1.005 | 1.001 | 1.008 |
| rs1782508 | 1.005 | 1.001 | 1.008 |
| rs1787013 | 1.005 | 1.001 | 1.008 |
| rs1799923 | 1.005 | 1.001 | 1.008 |
| rs1801282 | 1.005 | 1.001 | 1.008 |
| rs1808629 | 1.005 | 1.001 | 1.008 |
| rs1813039 | 1.005 | 1.001 | 1.008 |
| rs1861410 | 1.005 | 1.001 | 1.008 |
| rs1881505 | 1.005 | 1.001 | 1.008 |
| rs1906252 | 1.005 | 1.001 | 1.008 |
| rs1945160 | 1.005 | 1.001 | 1.008 |
| rs1964675 | 1.005 | 1.001 | 1.008 |
| rs1991002 | 1.005 | 1.001 | 1.008 |
| rs2002023 | 1.005 | 1.001 | 1.008 |
| rs2008018 | 1.005 | 1.001 | 1.008 |
| rs2034946 | 1.005 | 1.001 | 1.008 |
| rs2108635 | 1.005 | 1.001 | 1.008 |
| rs2111281 | 1.005 | 1.001 | 1.008 |
| rs215669 | 1.005 | 1.001 | 1.008 |
| rs2165991 | 1.005 | 1.001 | 1.008 |
| rs2172131 | 1.005 | 1.001 | 1.008 |
| rs217672 | 1.005 | 1.001 | 1.008 |
| rs2178899 | 1.005 | 1.001 | 1.008 |
| rs2190788 | 1.005 | 1.001 | 1.008 |
| rs2192527 | 1.005 | 1.001 | 1.008 |
| rs2192649 | 1.005 | 1.001 | 1.008 |
| rs2243928 | 1.005 | 1.001 | 1.008 |
| rs2276936 | 1.005 | 1.001 | 1.008 |
| rs2291127 | 1.005 | 1.001 | 1.008 |
| rs2307111 | 1.005 | 1.001 | 1.009 |
| rs2371767 | 1.005 | 1.001 | 1.008 |
| rs240999 | 1.005 | 1.001 | 1.008 |
| rs2415142 | 1.005 | 1.001 | 1.008 |
| rs2481899 | 1.005 | 1.001 | 1.009 |
| rs2499468 | 1.005 | 1.001 | 1.008 |
| rs2508782 | 1.005 | 1.001 | 1.008 |
| rs252749 | 1.005 | 1.001 | 1.008 |
| rs256904 | 1.005 | 1.001 | 1.008 |
| rs262953 | 1.005 | 1.001 | 1.008 |
| rs2640465 | 1.005 | 1.001 | 1.008 |
| rs2660241 | 1.005 | 1.001 | 1.009 |
| rs2678204 | 1.005 | 1.001 | 1.008 |
| rs2692741 | 1.005 | 1.001 | 1.008 |
| rs2702123 | 1.005 | 1.001 | 1.008 |
| rs2785988 | 1.005 | 1.001 | 1.008 |
| rs2802774 | 1.005 | 1.001 | 1.008 |
| rs2814993 | 1.005 | 1.001 | 1.008 |
| rs2855818 | 1.005 | 1.001 | 1.008 |
| rs28651380 | 1.005 | 1.001 | 1.008 |
| rs28672845 | 1.005 | 1.001 | 1.008 |
| rs28714450 | 1.005 | 1.001 | 1.008 |
| rs28742003 | 1.005 | 1.001 | 1.008 |
| rs2888778 | 1.005 | 1.001 | 1.008 |
| rs2943653 | 1.005 | 1.001 | 1.008 |
| rs2954033 | 1.005 | 1.001 | 1.008 |
| rs2957678 | 1.005 | 1.001 | 1.008 |
| rs2960420 | 1.005 | 1.001 | 1.008 |
| rs2966859 | 1.005 | 1.001 | 1.008 |
| rs2984618 | 1.005 | 1.001 | 1.008 |
| rs3113509 | 1.005 | 1.001 | 1.008 |
| rs319775 | 1.005 | 1.001 | 1.008 |
| rs33503 | 1.005 | 1.001 | 1.008 |
| rs33836 | 1.005 | 1.001 | 1.008 |
| rs34338229 | 1.005 | 1.001 | 1.008 |
| rs34483452 | 1.005 | 1.001 | 1.008 |
| rs34580448 | 1.005 | 1.001 | 1.008 |
| rs34656389 | 1.005 | 1.001 | 1.008 |
| rs35154152 | 1.005 | 1.001 | 1.008 |
| rs3743861 | 1.005 | 1.001 | 1.008 |
| rs3754963 | 1.005 | 1.001 | 1.008 |
| rs3764002 | 1.005 | 1.001 | 1.008 |
| rs3765971 | 1.005 | 1.001 | 1.008 |
| rs3766823 | 1.005 | 1.001 | 1.008 |
| rs3791709 | 1.005 | 1.001 | 1.008 |
| rs3803286 | 1.005 | 1.001 | 1.008 |
| rs3817428 | 1.005 | 1.001 | 1.008 |
| rs3826408 | 1.005 | 1.001 | 1.008 |
| rs3911063 | 1.005 | 1.001 | 1.008 |
| rs3923501 | 1.005 | 1.001 | 1.009 |
| rs394608 | 1.005 | 1.001 | 1.008 |
| rs396354 | 1.005 | 1.001 | 1.008 |
| rs40071 | 1.005 | 1.001 | 1.008 |
| rs41307479 | 1.005 | 1.001 | 1.008 |
| rs41310284 | 1.005 | 1.002 | 1.009 |
| rs429343 | 1.005 | 1.001 | 1.008 |
| rs429358 | 1.005 | 1.001 | 1.008 |
| rs4320040 | 1.005 | 1.001 | 1.008 |
| rs4398538 | 1.005 | 1.001 | 1.008 |
| rs441792 | 1.005 | 1.001 | 1.008 |
| rs4466418 | 1.005 | 1.001 | 1.008 |
| rs4482463 | 1.005 | 1.001 | 1.008 |
| rs4500770 | 1.005 | 1.001 | 1.008 |
| rs4547574 | 1.005 | 1.001 | 1.008 |
| rs4690324 | 1.005 | 1.001 | 1.008 |
| rs4709745 | 1.005 | 1.001 | 1.008 |
| rs4718964 | 1.005 | 1.001 | 1.008 |
| rs4722398 | 1.005 | 1.001 | 1.008 |
| rs4759318 | 1.005 | 1.001 | 1.008 |
| rs4762951 | 1.005 | 1.001 | 1.008 |
| rs4776337 | 1.005 | 1.001 | 1.008 |
| rs479018 | 1.005 | 1.001 | 1.008 |
| rs4790841 | 1.005 | 1.001 | 1.008 |
| rs4820323 | 1.005 | 1.001 | 1.009 |
| rs482787 | 1.005 | 1.001 | 1.008 |
| rs4876611 | 1.005 | 1.001 | 1.008 |
| rs4894808 | 1.005 | 1.001 | 1.008 |
| rs4908676 | 1.005 | 1.001 | 1.008 |
| rs4959613 | 1.005 | 1.001 | 1.008 |
| rs529200 | 1.005 | 1.001 | 1.008 |
| rs543874 | 1.005 | 1.001 | 1.009 |
| rs55707359 | 1.005 | 1.001 | 1.008 |
| rs55810445 | 1.005 | 1.001 | 1.008 |
| rs55924785 | 1.005 | 1.001 | 1.008 |
| rs56094641 | 1.005 | 1.001 | 1.008 |
| rs56218501 | 1.005 | 1.001 | 1.008 |
| rs56328878 | 1.005 | 1.001 | 1.008 |
| rs56399737 | 1.005 | 1.001 | 1.008 |
| rs57636386 | 1.005 | 1.001 | 1.008 |
| rs57800857 | 1.005 | 1.001 | 1.008 |
| rs58120873 | 1.005 | 1.001 | 1.008 |
| rs58862095 | 1.005 | 1.001 | 1.009 |
| rs59227842 | 1.005 | 1.001 | 1.009 |
| rs59499656 | 1.005 | 1.001 | 1.008 |
| rs6021948 | 1.005 | 1.001 | 1.008 |
| rs6064113 | 1.005 | 1.001 | 1.008 |
| rs6103254 | 1.005 | 1.001 | 1.008 |
| rs61754230 | 1.005 | 1.001 | 1.008 |
| rs61903695 | 1.005 | 1.001 | 1.008 |
| rs61910767 | 1.005 | 1.001 | 1.008 |
| rs61969510 | 1.005 | 1.001 | 1.008 |
| rs61975147 | 1.005 | 1.001 | 1.008 |
| rs61986205 | 1.005 | 1.001 | 1.008 |
| rs62107261 | 1.005 | 1.002 | 1.009 |
| rs62190394 | 1.005 | 1.001 | 1.008 |
| rs62218301 | 1.005 | 1.001 | 1.008 |
| rs62413414 | 1.005 | 1.001 | 1.008 |
| rs62443626 | 1.005 | 1.001 | 1.008 |
| rs62621197 | 1.005 | 1.001 | 1.008 |
| rs6480350 | 1.005 | 1.001 | 1.008 |
| rs6491427 | 1.005 | 1.001 | 1.008 |
| rs6500594 | 1.005 | 1.001 | 1.009 |
| rs6561937 | 1.005 | 1.001 | 1.008 |
| rs6567160 | 1.005 | 1.001 | 1.008 |
| rs6575340 | 1.005 | 1.001 | 1.009 |
| rs6602997 | 1.005 | 1.001 | 1.008 |
| rs6688826 | 1.005 | 1.001 | 1.008 |
| rs6693294 | 1.005 | 1.001 | 1.008 |
| rs6744646 | 1.005 | 1.001 | 1.008 |
| rs6749911 | 1.005 | 1.001 | 1.008 |
| rs6750646 | 1.005 | 1.001 | 1.008 |
| rs6752378 | 1.005 | 1.001 | 1.008 |
| rs6754292 | 1.005 | 1.001 | 1.008 |
| rs67609008 | 1.005 | 1.001 | 1.008 |
| rs6840236 | 1.005 | 1.001 | 1.008 |
| rs6847975 | 1.005 | 1.001 | 1.009 |
| rs685149 | 1.005 | 1.001 | 1.008 |
| rs6875585 | 1.005 | 1.001 | 1.008 |
| rs6927268 | 1.005 | 1.001 | 1.009 |
| rs6948959 | 1.005 | 1.001 | 1.008 |
| rs6973656 | 1.005 | 1.001 | 1.008 |
| rs6977416 | 1.005 | 1.001 | 1.008 |
| rs7020 | 1.005 | 1.001 | 1.008 |
| rs7027304 | 1.005 | 1.001 | 1.008 |
| rs704061 | 1.005 | 1.001 | 1.008 |
| rs7046679 | 1.005 | 1.001 | 1.008 |
| rs7124681 | 1.005 | 1.001 | 1.008 |
| rs7132908 | 1.005 | 1.001 | 1.008 |
| rs7133378 | 1.005 | 1.002 | 1.009 |
| rs71658797 | 1.005 | 1.001 | 1.008 |
| rs719802 | 1.005 | 1.001 | 1.008 |
| rs7206608 | 1.005 | 1.001 | 1.008 |
| rs7216121 | 1.005 | 1.001 | 1.008 |
| rs7218014 | 1.005 | 1.001 | 1.008 |
| rs72634814 | 1.005 | 1.001 | 1.008 |
| rs72681698 | 1.005 | 1.001 | 1.009 |
| rs72697297 | 1.005 | 1.001 | 1.009 |
| rs72703757 | 1.005 | 1.001 | 1.008 |
| rs72755233 | 1.005 | 1.001 | 1.008 |
| rs72767957 | 1.005 | 1.001 | 1.008 |
| rs72803260 | 1.005 | 1.001 | 1.008 |
| rs72892910 | 1.005 | 1.001 | 1.008 |
| rs72917533 | 1.005 | 1.001 | 1.008 |
| rs72976986 | 1.005 | 1.001 | 1.008 |
| rs72995085 | 1.005 | 1.001 | 1.009 |
| rs7321331 | 1.005 | 1.001 | 1.008 |
| rs73213501 | 1.005 | 1.001 | 1.008 |
| rs7357754 | 1.005 | 1.002 | 1.009 |
| rs74288880 | 1.005 | 1.001 | 1.008 |
| rs7442885 | 1.005 | 1.001 | 1.008 |
| rs74576293 | 1.005 | 1.001 | 1.008 |
| rs74618095 | 1.005 | 1.001 | 1.008 |
| rs7463186 | 1.005 | 1.001 | 1.008 |
| rs7498665 | 1.005 | 1.001 | 1.008 |
| rs75135487 | 1.005 | 1.001 | 1.008 |
| rs7535438 | 1.005 | 1.001 | 1.008 |
| rs75412871 | 1.005 | 1.001 | 1.008 |
| rs7575523 | 1.005 | 1.001 | 1.009 |
| rs7598246 | 1.005 | 1.001 | 1.008 |
| rs76115890 | 1.005 | 1.001 | 1.008 |
| rs7630228 | 1.005 | 1.001 | 1.008 |
| rs76345589 | 1.005 | 1.001 | 1.009 |
| rs7680610 | 1.005 | 1.001 | 1.008 |
| rs76856798 | 1.005 | 1.001 | 1.008 |
| rs7762794 | 1.005 | 1.001 | 1.008 |
| rs7773916 | 1.005 | 1.001 | 1.008 |
| rs7789056 | 1.005 | 1.001 | 1.008 |
| rs7796825 | 1.005 | 1.001 | 1.008 |
| rs78296744 | 1.005 | 1.001 | 1.008 |
| rs7843109 | 1.005 | 1.001 | 1.008 |
| rs78744936 | 1.005 | 1.001 | 1.008 |
| rs7893571 | 1.005 | 1.001 | 1.009 |
| rs7925725 | 1.005 | 1.001 | 1.008 |
| rs7942368 | 1.005 | 1.001 | 1.008 |
| rs79518326 | 1.005 | 1.001 | 1.008 |
| rs7960609 | 1.005 | 1.001 | 1.008 |
| rs7966251 | 1.005 | 1.001 | 1.008 |
| rs7972728 | 1.005 | 1.001 | 1.009 |
| rs7975187 | 1.005 | 1.001 | 1.008 |
| rs798549 | 1.005 | 1.001 | 1.008 |
| rs7987928 | 1.005 | 1.001 | 1.008 |
| rs8096564 | 1.005 | 1.001 | 1.008 |
| rs811054 | 1.005 | 1.001 | 1.008 |
| rs812949 | 1.005 | 1.001 | 1.008 |
| rs815163 | 1.005 | 1.001 | 1.009 |
| rs843901 | 1.005 | 1.001 | 1.008 |
| rs853961 | 1.005 | 1.001 | 1.008 |
| rs879620 | 1.005 | 1.001 | 1.008 |
| rs881929 | 1.005 | 1.001 | 1.009 |
| rs885114 | 1.005 | 1.001 | 1.008 |
| rs9289630 | 1.005 | 1.001 | 1.008 |
| rs9304665 | 1.005 | 1.001 | 1.008 |
| rs9321191 | 1.005 | 1.001 | 1.008 |
| rs9358912 | 1.005 | 1.001 | 1.008 |
| rs9372414 | 1.005 | 1.001 | 1.008 |
| rs9389857 | 1.005 | 1.001 | 1.008 |
| rs9568867 | 1.005 | 1.001 | 1.008 |
| rs957919 | 1.005 | 1.001 | 1.008 |
| rs9645335 | 1.005 | 1.001 | 1.008 |
| rs972283 | 1.005 | 1.001 | 1.008 |
| rs9788550 | 1.005 | 1.001 | 1.008 |
| rs9814758 | 1.005 | 1.001 | 1.008 |
| rs9843653 | 1.005 | 1.001 | 1.009 |
| rs9865173 | 1.005 | 1.001 | 1.008 |
| rs9892466 | 1.005 | 1.001 | 1.008 |
| rs9955276 | 1.005 | 1.001 | 1.008 |
| rs998584 | 1.005 | 1.001 | 1.009 |

OR, odds ratio.
