# Supplementary material for: Causal association between adiposity and hemorrhoids: a Mendelian randomization study
Source: Front Med (Lausanne). 2023 Oct 6;10:1229925. doi: 10.3389/fmed.2023.1229925 (PMC10587414; doi:10.3389/fmed.2023.1229925)
Supplement: Supplementary file 8 [file Table_8.docx]

Supplementary Table 8 Leave-one-out sensitivity analysis for the effect of waist circumference on haemorrhoids.

| Instrumental genetic variant | OR | 95% lower confidence interval | 95% upper confidence interval |
| --- | --- | --- | --- |
| All | 1.008 | 1.005 | 1.011 |
| rs1013402 | 1.008 | 1.005 | 1.011 |
| rs10150482 | 1.008 | 1.005 | 1.011 |
| rs10184230 | 1.008 | 1.005 | 1.011 |
| rs10185199 | 1.008 | 1.005 | 1.011 |
| rs1019240 | 1.008 | 1.005 | 1.011 |
| rs10236214 | 1.008 | 1.005 | 1.011 |
| rs10248298 | 1.008 | 1.005 | 1.011 |
| rs1025065 | 1.008 | 1.005 | 1.011 |
| rs10257197 | 1.008 | 1.005 | 1.011 |
| rs10269774 | 1.008 | 1.005 | 1.011 |
| rs1037702 | 1.008 | 1.005 | 1.011 |
| rs10423928 | 1.008 | 1.005 | 1.011 |
| rs10471636 | 1.008 | 1.005 | 1.011 |
| rs10490869 | 1.008 | 1.005 | 1.011 |
| rs10499014 | 1.008 | 1.005 | 1.011 |
| rs10505836 | 1.008 | 1.005 | 1.011 |
| rs1051613 | 1.008 | 1.005 | 1.011 |
| rs1056441 | 1.008 | 1.005 | 1.011 |
| rs10787738 | 1.008 | 1.005 | 1.011 |
| rs10803762 | 1.008 | 1.005 | 1.011 |
| rs10824211 | 1.008 | 1.005 | 1.011 |
| rs10827380 | 1.008 | 1.005 | 1.011 |
| rs10835676 | 1.008 | 1.005 | 1.011 |
| rs10938398 | 1.008 | 1.005 | 1.011 |
| rs10947793 | 1.008 | 1.005 | 1.011 |
| rs10957087 | 1.008 | 1.005 | 1.011 |
| rs10992854 | 1.008 | 1.005 | 1.011 |
| rs11012732 | 1.008 | 1.005 | 1.011 |
| rs11058233 | 1.008 | 1.005 | 1.011 |
| rs1108548 | 1.008 | 1.005 | 1.011 |
| rs11099020 | 1.008 | 1.005 | 1.011 |
| rs1111817 | 1.008 | 1.005 | 1.011 |
| rs111258054 | 1.008 | 1.005 | 1.011 |
| rs11150745 | 1.008 | 1.005 | 1.011 |
| rs11160600 | 1.008 | 1.005 | 1.011 |
| rs11162968 | 1.008 | 1.005 | 1.011 |
| rs11165493 | 1.008 | 1.005 | 1.011 |
| rs1117619 | 1.008 | 1.005 | 1.011 |
| rs11196657 | 1.008 | 1.005 | 1.011 |
| rs11215381 | 1.008 | 1.005 | 1.011 |
| rs11218510 | 1.008 | 1.005 | 1.011 |
| rs11223204 | 1.008 | 1.005 | 1.011 |
| rs113132247 | 1.008 | 1.005 | 1.011 |
| rs113866544 | 1.008 | 1.005 | 1.011 |
| rs114964326 | 1.008 | 1.005 | 1.011 |
| rs11603984 | 1.008 | 1.005 | 1.011 |
| rs11636611 | 1.008 | 1.005 | 1.011 |
| rs11639596 | 1.008 | 1.005 | 1.011 |
| rs11653367 | 1.008 | 1.005 | 1.011 |
| rs11675464 | 1.008 | 1.005 | 1.011 |
| rs11704728 | 1.008 | 1.005 | 1.011 |
| rs11757278 | 1.008 | 1.005 | 1.011 |
| rs11767811 | 1.008 | 1.005 | 1.011 |
| rs11773362 | 1.008 | 1.005 | 1.011 |
| rs11787216 | 1.008 | 1.005 | 1.011 |
| rs1182199 | 1.008 | 1.005 | 1.011 |
| rs11824092 | 1.008 | 1.005 | 1.011 |
| rs1183668 | 1.008 | 1.005 | 1.011 |
| rs11842871 | 1.008 | 1.005 | 1.011 |
| rs1188209 | 1.008 | 1.005 | 1.011 |
| rs11898037 | 1.008 | 1.005 | 1.011 |
| rs1191600 | 1.008 | 1.005 | 1.011 |
| rs12001437 | 1.008 | 1.005 | 1.011 |
| rs12042959 | 1.008 | 1.005 | 1.011 |
| rs12072739 | 1.008 | 1.005 | 1.011 |
| rs12103006 | 1.008 | 1.005 | 1.011 |
| rs12107172 | 1.008 | 1.005 | 1.011 |
| rs12140153 | 1.008 | 1.005 | 1.011 |
| rs1218824 | 1.008 | 1.005 | 1.011 |
| rs12225345 | 1.008 | 1.005 | 1.011 |
| rs12245654 | 1.008 | 1.005 | 1.011 |
| rs12273545 | 1.008 | 1.005 | 1.011 |
| rs12287076 | 1.008 | 1.005 | 1.011 |
| rs1229984 | 1.008 | 1.005 | 1.011 |
| rs12375196 | 1.008 | 1.005 | 1.011 |
| rs12462975 | 1.008 | 1.005 | 1.011 |
| rs12463617 | 1.008 | 1.005 | 1.011 |
| rs12478299 | 1.008 | 1.005 | 1.011 |
| rs12549000 | 1.008 | 1.005 | 1.011 |
| rs12877270 | 1.008 | 1.005 | 1.011 |
| rs12880641 | 1.008 | 1.005 | 1.011 |
| rs12926311 | 1.008 | 1.005 | 1.011 |
| rs1296328 | 1.008 | 1.005 | 1.011 |
| rs12983532 | 1.008 | 1.005 | 1.011 |
| rs13033310 | 1.008 | 1.005 | 1.011 |
| rs13047416 | 1.008 | 1.005 | 1.011 |
| rs13163306 | 1.008 | 1.005 | 1.011 |
| rs1320903 | 1.008 | 1.005 | 1.011 |
| rs1327259 | 1.008 | 1.005 | 1.011 |
| rs13288841 | 1.008 | 1.005 | 1.011 |
| rs13322435 | 1.008 | 1.005 | 1.011 |
| rs13333747 | 1.008 | 1.005 | 1.011 |
| rs1336486 | 1.008 | 1.005 | 1.011 |
| rs13410783 | 1.008 | 1.005 | 1.011 |
| rs13420048 | 1.008 | 1.005 | 1.011 |
| rs13427822 | 1.008 | 1.005 | 1.011 |
| rs1346841 | 1.008 | 1.005 | 1.011 |
| rs1357079 | 1.008 | 1.005 | 1.011 |
| rs1360201 | 1.008 | 1.005 | 1.011 |
| rs1411432 | 1.008 | 1.005 | 1.011 |
| rs1436348 | 1.008 | 1.005 | 1.011 |
| rs1441264 | 1.008 | 1.005 | 1.011 |
| rs145350287 | 1.008 | 1.005 | 1.011 |
| rs1458156 | 1.008 | 1.005 | 1.011 |
| rs1502317 | 1.008 | 1.005 | 1.011 |
| rs1559900 | 1.008 | 1.005 | 1.011 |
| rs1570298 | 1.008 | 1.005 | 1.011 |
| rs1582931 | 1.008 | 1.005 | 1.011 |
| rs1609010 | 1.008 | 1.005 | 1.011 |
| rs1609303 | 1.008 | 1.005 | 1.011 |
| rs1619442 | 1.008 | 1.005 | 1.011 |
| rs1625623 | 1.008 | 1.005 | 1.011 |
| rs1657930 | 1.008 | 1.005 | 1.011 |
| rs1711171 | 1.008 | 1.005 | 1.011 |
| rs17296856 | 1.008 | 1.005 | 1.011 |
| rs1731246 | 1.008 | 1.005 | 1.011 |
| rs17446091 | 1.008 | 1.005 | 1.011 |
| rs1752169 | 1.008 | 1.005 | 1.011 |
| rs17681738 | 1.008 | 1.005 | 1.011 |
| rs1788808 | 1.008 | 1.005 | 1.011 |
| rs1799923 | 1.008 | 1.005 | 1.011 |
| rs1834144 | 1.008 | 1.005 | 1.011 |
| rs1861410 | 1.008 | 1.005 | 1.011 |
| rs1902066 | 1.008 | 1.005 | 1.011 |
| rs1942826 | 1.008 | 1.005 | 1.011 |
| rs2020942 | 1.008 | 1.005 | 1.011 |
| rs2074881 | 1.008 | 1.005 | 1.011 |
| rs2133561 | 1.008 | 1.005 | 1.011 |
| rs215669 | 1.008 | 1.005 | 1.011 |
| rs2161097 | 1.008 | 1.005 | 1.011 |
| rs2172131 | 1.008 | 1.005 | 1.011 |
| rs217672 | 1.008 | 1.005 | 1.011 |
| rs2180454 | 1.008 | 1.005 | 1.011 |
| rs2183947 | 1.008 | 1.005 | 1.011 |
| rs2225909 | 1.008 | 1.005 | 1.011 |
| rs2253310 | 1.008 | 1.005 | 1.011 |
| rs2302209 | 1.008 | 1.005 | 1.011 |
| rs2306593 | 1.008 | 1.005 | 1.011 |
| rs2307111 | 1.008 | 1.005 | 1.011 |
| rs2376885 | 1.008 | 1.005 | 1.011 |
| rs2439823 | 1.008 | 1.005 | 1.011 |
| rs245767 | 1.008 | 1.005 | 1.011 |
| rs2470549 | 1.008 | 1.005 | 1.011 |
| rs2470946 | 1.008 | 1.005 | 1.011 |
| rs2482704 | 1.008 | 1.005 | 1.011 |
| rs2568958 | 1.008 | 1.005 | 1.011 |
| rs2584205 | 1.008 | 1.005 | 1.011 |
| rs2618039 | 1.008 | 1.005 | 1.011 |
| rs2678204 | 1.008 | 1.005 | 1.011 |
| rs2696309 | 1.008 | 1.005 | 1.011 |
| rs2725371 | 1.008 | 1.005 | 1.011 |
| rs2744938 | 1.008 | 1.005 | 1.011 |
| rs28350 | 1.008 | 1.005 | 1.011 |
| rs28366156 | 1.008 | 1.005 | 1.011 |
| rs28375268 | 1.008 | 1.005 | 1.011 |
| rs28489620 | 1.008 | 1.005 | 1.011 |
| rs2861692 | 1.008 | 1.005 | 1.011 |
| rs2903738 | 1.008 | 1.005 | 1.011 |
| rs3087523 | 1.008 | 1.005 | 1.011 |
| rs308911 | 1.008 | 1.005 | 1.011 |
| rs3113509 | 1.008 | 1.005 | 1.011 |
| rs319775 | 1.008 | 1.005 | 1.011 |
| rs3212038 | 1.008 | 1.005 | 1.011 |
| rs34045288 | 1.008 | 1.005 | 1.011 |
| rs34140906 | 1.008 | 1.005 | 1.011 |
| rs34234296 | 1.008 | 1.005 | 1.011 |
| rs34483452 | 1.008 | 1.005 | 1.011 |
| rs34517439 | 1.008 | 1.005 | 1.011 |
| rs34882821 | 1.008 | 1.005 | 1.011 |
| rs34994596 | 1.008 | 1.005 | 1.011 |
| rs35023999 | 1.008 | 1.005 | 1.011 |
| rs35216639 | 1.008 | 1.005 | 1.011 |
| rs35243581 | 1.008 | 1.005 | 1.011 |
| rs35681682 | 1.008 | 1.005 | 1.011 |
| rs35882248 | 1.008 | 1.005 | 1.011 |
| rs36007635 | 1.008 | 1.005 | 1.011 |
| rs36061954 | 1.008 | 1.005 | 1.011 |
| rs36140 | 1.008 | 1.005 | 1.011 |
| rs36165342 | 1.008 | 1.005 | 1.011 |
| rs3764002 | 1.008 | 1.005 | 1.011 |
| rs3768321 | 1.008 | 1.005 | 1.011 |
| rs3784692 | 1.008 | 1.005 | 1.011 |
| rs3806114 | 1.008 | 1.005 | 1.011 |
| rs3807566 | 1.008 | 1.005 | 1.011 |
| rs3814883 | 1.008 | 1.005 | 1.011 |
| rs3826408 | 1.008 | 1.005 | 1.011 |
| rs3845344 | 1.008 | 1.005 | 1.011 |
| rs3866805 | 1.008 | 1.005 | 1.011 |
| rs3935190 | 1.008 | 1.005 | 1.011 |
| rs3936510 | 1.008 | 1.005 | 1.011 |
| rs400031 | 1.008 | 1.005 | 1.011 |
| rs40067 | 1.008 | 1.005 | 1.011 |
| rs4072917 | 1.008 | 1.005 | 1.011 |
| rs4075353 | 1.008 | 1.005 | 1.011 |
| rs41279738 | 1.008 | 1.005 | 1.011 |
| rs4148155 | 1.008 | 1.005 | 1.011 |
| rs4290163 | 1.008 | 1.005 | 1.011 |
| rs429343 | 1.008 | 1.005 | 1.011 |
| rs429358 | 1.008 | 1.005 | 1.011 |
| rs4344019 | 1.008 | 1.005 | 1.011 |
| rs4419475 | 1.008 | 1.005 | 1.011 |
| rs4456769 | 1.008 | 1.005 | 1.011 |
| rs4469245 | 1.008 | 1.005 | 1.011 |
| rs4482463 | 1.008 | 1.005 | 1.011 |
| rs4525978 | 1.008 | 1.005 | 1.011 |
| rs4527444 | 1.008 | 1.005 | 1.011 |
| rs4552632 | 1.008 | 1.005 | 1.011 |
| rs4689465 | 1.008 | 1.005 | 1.011 |
| rs4706004 | 1.008 | 1.005 | 1.011 |
| rs4718964 | 1.008 | 1.005 | 1.011 |
| rs4722398 | 1.008 | 1.005 | 1.011 |
| rs4742782 | 1.008 | 1.005 | 1.011 |
| rs4790841 | 1.008 | 1.005 | 1.011 |
| rs484455 | 1.008 | 1.005 | 1.011 |
| rs4844809 | 1.008 | 1.005 | 1.011 |
| rs4851283 | 1.008 | 1.005 | 1.011 |
| rs4876611 | 1.008 | 1.005 | 1.011 |
| rs4900715 | 1.008 | 1.005 | 1.011 |
| rs4908672 | 1.008 | 1.005 | 1.011 |
| rs520478 | 1.008 | 1.005 | 1.011 |
| rs539515 | 1.008 | 1.005 | 1.011 |
| rs55726687 | 1.008 | 1.005 | 1.011 |
| rs55794894 | 1.008 | 1.005 | 1.011 |
| rs557951 | 1.008 | 1.005 | 1.011 |
| rs559231 | 1.008 | 1.005 | 1.011 |
| rs56094641 | 1.008 | 1.005 | 1.012 |
| rs56803094 | 1.008 | 1.005 | 1.011 |
| rs57636386 | 1.008 | 1.005 | 1.011 |
| rs587271 | 1.008 | 1.005 | 1.011 |
| rs58862095 | 1.008 | 1.005 | 1.011 |
| rs588660 | 1.008 | 1.005 | 1.011 |
| rs59068084 | 1.008 | 1.005 | 1.011 |
| rs59104534 | 1.008 | 1.005 | 1.011 |
| rs6001877 | 1.008 | 1.005 | 1.011 |
| rs6030803 | 1.008 | 1.005 | 1.011 |
| rs6069037 | 1.008 | 1.005 | 1.011 |
| rs61223906 | 1.008 | 1.005 | 1.011 |
| rs61813324 | 1.008 | 1.005 | 1.011 |
| rs61903695 | 1.008 | 1.005 | 1.011 |
| rs61969511 | 1.008 | 1.005 | 1.011 |
| rs61992671 | 1.008 | 1.005 | 1.011 |
| rs62072003 | 1.008 | 1.005 | 1.011 |
| rs62243489 | 1.008 | 1.005 | 1.011 |
| rs62246311 | 1.008 | 1.005 | 1.011 |
| rs62261725 | 1.008 | 1.005 | 1.011 |
| rs6493498 | 1.008 | 1.005 | 1.011 |
| rs649458 | 1.008 | 1.005 | 1.011 |
| rs6536575 | 1.008 | 1.005 | 1.011 |
| rs6551304 | 1.008 | 1.005 | 1.011 |
| rs6567160 | 1.008 | 1.005 | 1.011 |
| rs6575340 | 1.008 | 1.005 | 1.011 |
| rs6669341 | 1.008 | 1.005 | 1.011 |
| rs6682438 | 1.008 | 1.005 | 1.011 |
| rs6693294 | 1.008 | 1.005 | 1.011 |
| rs6739755 | 1.008 | 1.005 | 1.011 |
| rs67609008 | 1.008 | 1.005 | 1.011 |
| rs67632512 | 1.008 | 1.005 | 1.011 |
| rs6791983 | 1.008 | 1.005 | 1.011 |
| rs6846041 | 1.008 | 1.005 | 1.011 |
| rs6849518 | 1.008 | 1.005 | 1.011 |
| rs6938973 | 1.008 | 1.005 | 1.011 |
| rs7034554 | 1.008 | 1.005 | 1.011 |
| rs704061 | 1.008 | 1.005 | 1.011 |
| rs7070670 | 1.008 | 1.005 | 1.011 |
| rs7115013 | 1.008 | 1.005 | 1.011 |
| rs7132908 | 1.008 | 1.005 | 1.011 |
| rs71495038 | 1.008 | 1.005 | 1.011 |
| rs7169847 | 1.008 | 1.005 | 1.011 |
| rs7171864 | 1.008 | 1.005 | 1.011 |
| rs7206608 | 1.008 | 1.005 | 1.011 |
| rs7218014 | 1.008 | 1.005 | 1.011 |
| rs7259070 | 1.008 | 1.005 | 1.011 |
| rs72617140 | 1.008 | 1.005 | 1.011 |
| rs72618637 | 1.008 | 1.005 | 1.011 |
| rs72634826 | 1.008 | 1.005 | 1.011 |
| rs72892910 | 1.008 | 1.005 | 1.011 |
| rs72959041 | 1.008 | 1.005 | 1.011 |
| rs72976986 | 1.008 | 1.005 | 1.011 |
| rs73052033 | 1.008 | 1.005 | 1.011 |
| rs73068448 | 1.008 | 1.005 | 1.011 |
| rs73142879 | 1.008 | 1.005 | 1.011 |
| rs7324067 | 1.008 | 1.005 | 1.011 |
| rs735033 | 1.008 | 1.005 | 1.011 |
| rs7372674 | 1.008 | 1.005 | 1.011 |
| rs7377083 | 1.008 | 1.005 | 1.011 |
| rs73985439 | 1.008 | 1.005 | 1.011 |
| rs74395133 | 1.008 | 1.005 | 1.011 |
| rs7442885 | 1.008 | 1.005 | 1.011 |
| rs7498044 | 1.008 | 1.005 | 1.011 |
| rs7498665 | 1.008 | 1.005 | 1.011 |
| rs75035127 | 1.008 | 1.005 | 1.011 |
| rs7519259 | 1.008 | 1.005 | 1.011 |
| rs7537581 | 1.008 | 1.005 | 1.011 |
| rs756717 | 1.008 | 1.005 | 1.011 |
| rs76040172 | 1.008 | 1.005 | 1.011 |
| rs76286777 | 1.008 | 1.005 | 1.011 |
| rs7630382 | 1.008 | 1.005 | 1.011 |
| rs765876 | 1.008 | 1.005 | 1.011 |
| rs76895963 | 1.008 | 1.005 | 1.011 |
| rs7708584 | 1.008 | 1.005 | 1.011 |
| rs77165542 | 1.008 | 1.005 | 1.012 |
| rs7752202 | 1.008 | 1.005 | 1.011 |
| rs7755574 | 1.008 | 1.005 | 1.011 |
| rs784257 | 1.008 | 1.005 | 1.011 |
| rs7845090 | 1.008 | 1.005 | 1.011 |
| rs7925100 | 1.008 | 1.005 | 1.011 |
| rs7933085 | 1.008 | 1.005 | 1.011 |
| rs7952436 | 1.008 | 1.005 | 1.011 |
| rs7966251 | 1.008 | 1.005 | 1.011 |
| rs8013377 | 1.008 | 1.005 | 1.011 |
| rs8024137 | 1.008 | 1.005 | 1.011 |
| rs80243702 | 1.008 | 1.005 | 1.011 |
| rs8078135 | 1.008 | 1.005 | 1.011 |
| rs8097672 | 1.008 | 1.005 | 1.011 |
| rs8112818 | 1.008 | 1.005 | 1.011 |
| rs815163 | 1.008 | 1.005 | 1.011 |
| rs8192675 | 1.008 | 1.005 | 1.011 |
| rs852042 | 1.008 | 1.005 | 1.011 |
| rs852983 | 1.008 | 1.005 | 1.011 |
| rs862227 | 1.008 | 1.005 | 1.011 |
| rs862320 | 1.008 | 1.005 | 1.011 |
| rs876605 | 1.008 | 1.005 | 1.011 |
| rs879620 | 1.008 | 1.005 | 1.011 |
| rs883403 | 1.008 | 1.005 | 1.011 |
| rs894736 | 1.008 | 1.005 | 1.011 |
| rs923994 | 1.008 | 1.005 | 1.011 |
| rs9289630 | 1.008 | 1.005 | 1.011 |
| rs9294260 | 1.008 | 1.005 | 1.011 |
| rs9308964 | 1.008 | 1.005 | 1.011 |
| rs9316661 | 1.008 | 1.005 | 1.011 |
| rs9370243 | 1.008 | 1.005 | 1.011 |
| rs9378676 | 1.008 | 1.005 | 1.011 |
| rs945211 | 1.008 | 1.005 | 1.011 |
| rs9478496 | 1.008 | 1.005 | 1.011 |
| rs9568867 | 1.008 | 1.005 | 1.011 |
| rs9584870 | 1.008 | 1.005 | 1.011 |
| rs9654453 | 1.008 | 1.005 | 1.011 |
| rs9673839 | 1.008 | 1.005 | 1.011 |
| rs9814758 | 1.008 | 1.005 | 1.011 |
| rs9843653 | 1.008 | 1.005 | 1.011 |
| rs9888533 | 1.008 | 1.005 | 1.011 |
| rs9902846 | 1.008 | 1.005 | 1.011 |
| rs9916444 | 1.008 | 1.005 | 1.011 |
| rs9926784 | 1.008 | 1.005 | 1.011 |

OR, odds ratio.
