# Supplementary material for: Causal association between adiposity and hemorrhoids: a Mendelian randomization study
Source: Front Med (Lausanne). 2023 Oct 6;10:1229925. doi: 10.3389/fmed.2023.1229925 (PMC10587414; doi:10.3389/fmed.2023.1229925)
Supplement: Supplementary file 9 [file Table_9.docx]

Supplementary Table 9 Leave-one-out sensitivity analysis for the effect of waist-to-hip ratio on haemorrhoids.

| Instrumental genetic variant | OR | 95% lower confidence interval | 95% upper confidence interval |
| --- | --- | --- | --- |
| All | 1.010 | 1.003 | 1.017 |
| rs1011731 | 1.010 | 1.003 | 1.017 |
| rs10245353 | 1.011 | 1.004 | 1.018 |
| rs10783615 | 1.011 | 1.004 | 1.018 |
| rs11048470 | 1.011 | 1.004 | 1.018 |
| rs1121980 | 1.011 | 1.004 | 1.018 |
| rs1128249 | 1.010 | 1.003 | 1.017 |
| rs11663816 | 1.010 | 1.003 | 1.017 |
| rs12549058 | 1.011 | 1.004 | 1.018 |
| rs1294421 | 1.010 | 1.003 | 1.017 |
| rs1316952 | 1.009 | 1.003 | 1.016 |
| rs1440372 | 1.010 | 1.003 | 1.017 |
| rs1563355 | 1.011 | 1.004 | 1.018 |
| rs1569135 | 1.010 | 1.003 | 1.017 |
| rs16996700 | 1.010 | 1.003 | 1.017 |
| rs17109256 | 1.010 | 1.004 | 1.017 |
| rs17451107 | 1.009 | 1.002 | 1.015 |
| rs2179129 | 1.010 | 1.003 | 1.017 |
| rs2287019 | 1.010 | 1.004 | 1.017 |
| rs2765539 | 1.009 | 1.002 | 1.015 |
| rs2972164 | 1.010 | 1.003 | 1.017 |
| rs3786897 | 1.011 | 1.004 | 1.018 |
| rs459193 | 1.009 | 1.002 | 1.016 |
| rs4640244 | 1.010 | 1.003 | 1.017 |
| rs4715213 | 1.010 | 1.003 | 1.017 |
| rs4929927 | 1.010 | 1.003 | 1.017 |
| rs929641 | 1.010 | 1.003 | 1.017 |
| rs9860730 | 1.011 | 1.005 | 1.018 |
| rs998584 | 1.009 | 1.002 | 1.016 |

OR, odds ratio.
